# Efficiency of multivariate tests in trials in progressive supranuclear palsy

**DOI:** 10.1038/s41598-024-76668-4

**Published:** 2024-10-26

**Authors:** Elham Yousefi, Mohamed Gewily, Franz König, Günter Höglinger, Franziska Hopfner, Mats O. Karlsson, Robin Ristl, Sonja Zehetmayer, Martin Posch

**Affiliations:** 1 Center for Medical Data Science, Medical University of Vienna, Vienna, Austria; 2 Department of Pharmacy, Uppsala University, Uppsala, Sweden; 3 Department of Neurology, LMU University Hospital, Ludwig-Maximilians-Universität (LMU) München, Munich, Germany; 4 German Center for Neurodegenerative Diseases (DZNE), Munich, Germany; 5 Munich Cluster for Systems Neurology (SyNergy), Munich, Germany; 6 Department of Neurology, Hannover Medical School, Hanover, Germany

**Keywords:** Progressive supranuclear palsy, Clinical trials, Multiple endpoints, Simulation study, Item response theory, Multivariate tests, Clinical trial design, Biostatistics

## Abstract

Measuring disease progression in clinical trials for testing novel treatments for multifaceted diseases as progressive supranuclear palsy (PSP), remains challenging. In this study we assess a range of statistical approaches to compare outcomes as measured by the items of the progressive supranuclear palsy rating scale (PSPRS). We consider several statistical approaches, including sum scores, a modified PSPRS rating scale that had been recommended by FDA in a pre-IND meeting, multivariate tests, and analysis approaches based on multiple comparisons of the individual items. In addition, we propose two novel approaches which measure disease status based on Item Response Theory models. We assess the performance of these tests under various scenarios in an extensive simulation study and illustrate their use with a re-analysis of the ABBV-8E12 clinical trial. Furthermore, we discuss the impact of the FDA-recommended scoring of item scores on the power of the statistical tests. We find that classical approaches as the PSPRS sum score demonstrate moderate to high power when treatment effects are consistent across the individual items. The tests based on Item Response Theory (IRT) models yield the highest power when the simulated data are generated from an IRT model. The multiple testing based approaches have a higher power in settings where the treatment effect is limited to certain domains or items. The study demonstrates that there is no one-size-fits-all testing procedure for evaluating treatment effects using PSPRS items; the optimal method varies based on the specific effect size patterns. The efficiency of the PSPRS sum score, while generally robust and straightforward to apply, varies depending on the specific patterns of effect sizes encountered and more powerful alternatives are available in specific settings. These findings can have important implications for the design of future clinical trials in PSP and similar multifaceted diseases.

## Introduction

In diseases that exhibit multifaceted manifestations, disease progression cannot be characterised with a single measurement. Instead, multiple characteristics have to be assessed to describe the disease status^1^. This poses a challenge to define appropriate endpoints in clinical trials to assess the effect of investigational treatments on the disease progression.

In this paper, we assess a wide range of statistical approaches that have been proposed to define and analyse endpoints for clinical trials in indications where disease progression is measured by several variables. We focus on the setting of clinical trials for progressive supranuclear palsy (PSP), a rare neurodegenerative disorder with complex symptoms that affect balance, vision, body movements, and speech, ultimately leading to death. The most commonly used endpoint is the progressive supranuclear palsy rating scale (PSPRS-28), a 28-item method to measure progression of the disease^2^. The item scores are categorical values which mostly vary from zero to 4. The PSPRS-28 is then defined as a sum score, summing the item scores of all 28-items.

Recently, several double-blind, placebo-controlled, randomised, clinical trials to investigate the efficacy of therapeutic interventions in PSP have been conducted which used the PSPRS-28 as primary endpoint^3–7^. However, so far no specific symptomatic treatments or disease-modifying therapies are approved^8,9^. Tolosa et al.^4^ investigated tideglusib, a glycogen synthase kinase 3 inhibitor, in a phase 2 trial in 146 PSP patients. The primary endpoint was the change from baseline to week 52 on the PSPRS. No statistically significant treatment effect was demonstrated. Boxer et al.^3^ performed a randomised, double-blind, placebo-controlled, phase 2/3 trial of davunetide with 313 participants, using the change in the PSP Rating Scale and Schwab and England Activities of Daily Living scale at up to 52 weeks as primary endpoints also showing no significant treatment effect. The PROSPERA trial^5^ investigated rasagiline, an MAO-B inhibitor, in a 1-year, randomised, double-blind, placebo-controlled study with 44 PSP patients. The combined primary endpoint was the PSPRS-28 and the need for L-dopa rescue medication. Individual PSPRS-28 changes were expressed as Wilcoxon ranks of the areas under the curves of the PSPRS-28 over time. Increase in L-dopa medication was a binary variable. The trial found no significant effect on the primary endpoint. Another trial evaluated gosuranemab^6^, an anti-tau monoclonal antibody, in a 52-week, randomised, double-blind, placebo-controlled study with 486 PSP patients. Primary endpoint was the adjusted mean change in the PSP Rating Scale score at week 52. Also this trial found no significant difference in PSP Rating Scale scores between the treatment groups. While gosuranemab significantly reduced unbound N-terminal tau in cerebrospinal fluid compared to placebo, this reduction did not translate into clinical efficacy. Höglinger et al.^7^ assessed two doses of tilavonemab, a monoclonal antibody against tau protein, in a randomised, placebo-controlled, double-blind, phase 2 trial in 378 patients. The primary endpoint was the change from baseline to week 52 in the PSPRS. No efficacy in the primary endpoint was demonstrated (see section “Case study” for a more detailed description of this trial).

The use of a sum score, such as the PSPRS-28 or its modifications, is a standard approach to combining information from multiple endpoints. By using such an aggregated score, standard statistical tests like an analysis of covariance can be applied to compare the outcome variable, or its change from baseline, between groups.

Another approach to demonstrate efficacy with an overall test involves multivariate testing procedures^10,11^. These procedures aggregate the test statistics of comparisons of individual characteristics into a single univariate test statistic and test the global null hypothesis that the expected value of each item score is larger or equal in the treatment group compared to the control group. O’Brien proposed two global directional tests known as Ordinary Least Squares (OLS) and Generalised Least Squares (GLS) tests to demonstrate an overall treatment effect^10^. Global directional tests are proposed in settings, where treatment benefit corresponds to a change into the same direction in all item scores. In such settings, two-sided tests, such as Hotelling’s $T^2$ test, which do not account for the direction of treatment differences, are not of interest and will therefore not be considered further^12^.

An alternative approach is to consider the individual components of the multivariate endpoint separately. In this case, inference is based on the individual test statistics or the individual p-values for the multiple endpoints and a multiplicity correction is applied to control the familywise error rate in the strong sense. Control of the familywise error rate can be achieved by the Bonferroni procedure or more powerful multiple tests that take the dependence between the endpoints into account.

A further univariate test we consider is based on an Item Response Theory (IRT) model^13^. As endpoint we consider for each patient the value of the latent variable of the IRT model, which describes the disease status and can be predicted based on estimating the IRT model parameters given the data (or the scores). As the estimation of the latent variable from the item scores is complex we propose also to approximate the latent variable estimate using linear models. Specifically, we approximate the latent variable by a weighted sum of the item scores. This linear model-based estimation makes the endpoint more interpretable and yields a similar endpoint as the PSPRS-10, however, using a weighted sum with weights derived from the IRT model (see section “Analysis methods”).

We aim to identify the most powerful statistical tests to assess treatment effects under various scenarios. To compare the power of the different procedures, we perform a comprehensive simulation study. Especially, we consider different strategies to simulate data of the PSPRS item scores, including the discretisation of multivariate normal outcomes as well as resampling from actual clinical trial data from the ABBV-8E12 trial^7^. In addition, we consider a range of alternative hypotheses to cover settings where there is a treatment effect in all item scores or only in a subdomain or even in a single item score only. Additionally, we investigate the impact of the scoring of the items suggested by the FDA, on the power of the considered statistical methods, as well as the case where the original scoring is considered (See section “The progressive supranuclear palsy rating scale (PSPRS)”).

This paper is structured as follows: Section “Case study” discusses the ABBV-8E12 trial, forming the basis of our analysis. Section “The progressive supranuclear palsy rating scale (PSPRS)” describes the PSPRS and its FDA modification. The considered analysis approaches are detailed in section “Analysis methods” , while section “Simulation study” describes the different simulation approaches for the item level data as well as the simulation results. A re-analysis of a clinical trial is provided in section “Reanalysis of the ABBV-8E12 trial”. The paper concludes with a discussion. Technical details on multivariate tests are provided in Section 3 of Supplementary Material A.

## Case study

As a case study to inform the trial designs and simulation of trial data in the simulation study, we considered the ABBV-8E12 trial^7^. The ABBV-8E12 trial was a randomised, double-blind, three-armed parallel group trial comparing a 2000 mg and 4000 mg dose of the investigational compound tilavonemab to placebo.

In the ABBV-8E12 trial, 378 patients were randomised and a total of 377 patients received at least one dose of the investigational treatment or placebo (tilavonemab 2000 mg $n=126$, tilavonemab 4000 mg $n=125$, placebo $n=126$). The original PSPRS-28 items were assessed at baseline and weeks 12, 24, 36 and 52, where the change from baseline to week 52 in the PSPRS-28 total score was the main outcome variable. To assess the efficacy of treatments, the change from baseline to week 52 was analysed using a mixed-effect repeated measure model. The Bonferroni approach was used to account for multiplicity due to multiple comparisons between the two doses and placebo groups. The trial was terminated early due to futility. The change from baseline to week 52 in PSPRS-28 total score was similar between the three treatment groups at all considered visits and no significance difference between treatment groups, in favor of tilavonemab 2000 mg or tilavonemab 4000 mg versus placebo, was observed.

To investigate the different testing approaches, below we considered a simplified study design with only one treatment-control comparison.

## Methods

For the ABBV-8E12 trial^7^, whose data are used in this reanalysis, all participants and their respective study partners were required to provide written informed consent before screening or any study specific procedures. The study received independent ethics committee or institutional review board approval at each study site before initiation. The study adhered to all applicable local regulations, was done in accordance with Good Clinical Practice, as outlined by the International Conference on Harmonisation, and complied with ethical standards described in the Declaration of Helsinki. See^7^ for further details.

The study protocol for this reanalysis was approved by the ethics committee of the Hannover Medical School, Hanover, Germany (No $9400\_BO\_K\_2020$) and all methods were performed in accordance with the relevant guidelines and regulations.

### The progressive supranuclear palsy rating scale (PSPRS)

In randomised, phase 2, placebo-controlled trials of experimental treatments of PSP, the standard method to test for a treatment effect is to use the sum of the 28 item scores (the so called total score) of the progressive supranuclear palsy rating scale (PSPRS) as the primary endpoint variable. Changes in this endpoint are compared between treatment groups, with higher scores indicating more severe disease conditions. The scale is based on the physicians’ assessment and is measured repeatedly to assess disease progression across different visits in the trials. Recently, in a pre-IND meeting with Amylyx Pharmaceuticals, FDA has recommended to use a modified version of the PSPRS, which includes a subset of 10 items (Table 1). In addition, FDA recommended to collapse some of the item levels of the 10-item version of the PSPRS. This re-scoring has been recommended by FDA for all but two items, since it was argued that the original response levels for some items do not reflect clear and clinically meaningful differences of the patients’ conditions and are therefore difficult to interpret. In the remainder of this manuscript, we refer to the original PSPRS with all 28 items as PSPRS-28 and the scale with the FDA-recommended items as PSPRS-10. Furthermore, we will consider the PSPRS-10 scale with the original scoring used in the original PSPRS-28 (which we call *original scoring*) as well as with the rescored item levels, which we call *PSPRS-10 scoring*. The full version of the PSPRS-28 which includes the subset of 10 items suggested by the FDA is provided as a table in the online Supplement B. The table contains the original scoring and the PSPRS-10 scoring, including a description on how to collapse the item levels.

**Table 1:** FDA-recommended PSPRS-10.

Full name	Abbreviation
I. HISTORY (from patient or other informant)	
3. Dysphagia for solids (from patient or other informant)	Dysp.FS
4. Using knife and fork, buttoning clothes, washing hands and face (rate the worst)	Use.KF
5. Falls (average frequency if patient attempted to walk unaided)	Fall
III. BULBAR EXAM	
12. Dysarthria (ignoring palilalia)	Dysa.
13. Dysphagia (for 30-50 cc of water from a cup, if safe)	Dysp.
VI. GAIT/MIDLINE EXAM	
24. Neck rigidity or dystonia	Neck.Ri
25. Arising from chair	Ari.FC
26. Gait	Gait
27. Postural stability (on backward pull)	Pos.St
28. Sitting down (may touch seat or back but not arms of chair)	Sit

The 10 items are divided into 3 domains (History, Bulbar exam, Gait/Midline Exam). The numbering of the items corresponds to the original PSPRS-28^2^. The column “Abbreviation” gives the short name used to label the items in the subsequent graphs.

In this work we focus on the PSPRS-10 proposed by FDA with the original and the PSPRS-10 scoring. The reduction of the scale to 10 items is supported by an IRT analysis by^14^ who showed that these items are the most informative to describe the course of the disease. As in the ABBV-8E12 trial^7^, we assume that the primary endpoint is based on the item scores measured at week 52, corresponding to a 1 year observation period, and that the analysis adjusts for the baseline measurements.

### Analysis methods

All the analyses below were performed based on the PSPRS-10 with the original scoring as well as the PSPRS-10 scoring.

With the exception of the IRT based test (but not the approximate IRT based test), all tests below test the null hypothesis that the expected values of all of the 10 item scores are larger or equal in the treatment group than in the control group. We term this null hypothesis as the strong null hypothesis. The corresponding alternative hypothesis is that the expected values of at least one item score is strictly smaller in the treatment group compared to the control group. Some of the tests in addition are valid tests for broader null hypotheses as indicated below.

#### PSPRS-10 with the original and PSPRS-10 scoring

As benchmark, we used an analysis of covariance (ANCOVA) for the PSPRS-10 scale with the original or the PSPRS-10 scoring at week 52 as dependent variable and the respective PSPRS-10 at baseline as well as the treatment indicator as independent variables. The (t-)test for the coefficient for the treatment variable then tests the null hypothesis of no treatment effect. Note that this adjusted analysis is equivalent to a comparison of the change from baseline between the two groups, when the baseline value is adjusted for as a covariate in an analysis of covariance^15^. Furthermore, this test not only tests the strong null hypothesis, but also the null hypothesis that the mean of the item scores (across items and the patient population) is larger or equal in the treatment compared to the control group.

#### IRT-based test

 The second considered, univariate test is based on estimates of the latent variable based on an IRT model^16^. IRT models represent the state of disease as a latent variable which is defined based on multiple reported measures. In PSP, the latent variable corresponding to the disease severity is measured through the PSPRS item questionnaire as reported by the physician. To perform the IRT-based test, we require an IRT model fitted on an external data set. We used a graded response (GR) model^17^, fitted to the ordered polytomous item scores of the PSPRS-10 from the ABBV-8E12 trial. To this end, we first pooled the data across treatment groups and visits (including the baseline and follow-up visits at weeks 12, 24, 36 and 52) such that the data from each visit became an independent individual (row) in the data set. Based on this data set, we fitted an IRT model. The parameter estimates of the GR model are presented in Supplementary Table 24. This simplified analysis does not account for the dependence of the measurements from a patient at different visits, but accounts for the dependence of a patient’s measurements of the different items within a single visit. This allowed us to estimate the IRT model based on a larger data set with the aim to enhance the precision of the model estimates.

Based on this a-priori estimated model, we then estimate the latent trait variable for the patients in the actual clinical trial based on the observed item scores, separately for the baseline and the 52-weeks data. Especially, we estimate the predicted latent trait variable with the expected a-posteriori (EAP) method^18^, which is implemented, for example, in the R package mirt for the analysis of dichotomous/polytomous response data using latent trait models under the IRT paradigm^19^.

Finally, an ANCOVA model is fitted with the predicted latent trait variable at week 52 as dependent and the latent trait variable at baseline and treatment as independent variables. This procedure tests the null hypothesis that the expected value of the latent variables is larger in the treatment group than the control group.

This procedure tests the null hypothesis that the expected values of the latent variable estimate in the treatment group is larger or equal than in the control group. Especially this null hypothesis holds if the joint distribution of item scores in the two treatment groups is equal. However, given the complex dependence of the latent variable estimates on the item scores, it is not obvious if this null hypothesis is included in the strong null hypothesis defined above.

#### Approximate IRT-based test

 As the estimation of the latent variable with IRT model is complex and the impact of the individual item scores is not immediately understandable without going through complex derivations, we aim to approximate the estimate by a weighted sum of the item scores. This aims to make the endpoint more interpretable. Therefore, we consider as endpoint a weighted sum of the item scores, where the weights are derived from a linear model fitted on the data of an external trial. We used again data of the ABBV-8E12 trial, where, as above, we aggregated the data from all treatment groups and visits, fitted an IRT model and estimated the latent variable for each patient. Then, using the same data set, we fitted a linear model with the latent variable as dependent variable and the 10 FDA recommended items as independent variable. Note that the latent variable was transformed with the standard logistic function to improve model fit. Based on this a-priori estimated model, we then estimate the (approximate) latent trait variable for the patients in the actual clinical trial by computing the weighted sum of the observed item scores and back transforming it (using the standard logit function) to match the original scale. Note that in principle the linear model could give values outside the unit interval such that the standard logit function cannot be applied. If this occurs one can truncate the values accordingly. For our data set this was not an issue as the corresponding fitted linear model only gives values in the unit interval. In addition to the strong null hypothesis defined above, this procedure tests the null hypothesis that the expected value of the resulting weighted sum is larger in the treatment compared to the control group.

#### OLS and GLS tests

 The O’Brien’s OLS and GLS tests^10^ are multivariate tests combining test statistics of separate tests comparing the responses for each of the items between groups to a common test statistics. The OLS test is based on the standardised, unweighted sum of t-statistics of the individual items, see Eq. (1) in Supplementary Material A. The GLS test, in contrast, is based on a weighted sum, determined by the row sums of the inverse of the correlation matrix (see Eq. (2) in Supplementary Material A). The t-statistics for the comparison of the individual item scores are obtained from an ANCOVA model, with the item score at week 52 as the dependent variable and the baseline item score and treatment indicator as independent variables. The correlation of the test statistics, required to standardise the test statistics and, for the GLS test, to compute the weights, is estimated using multiple marginal modeling (using the mmm function^20^ in the multcomp package in R). Endpoints that are highly correlated receive lower weights in the GLS test statistic. However, for small samples^21^ showed (via simulations) that the approximation of the type 1 error rate for the GLS test can be very liberal depending on the true correlation matrix and is not corrected by the different approximations for the degrees of freedom of the corresponding t-distribution. Additionally, it has been noted in the literature that the GLS weights can become negative^22^. This implies that the corresponding test may no longer be a directional test, but can inflate the type I error rate if there is a negative effect in some of the items and no effect in the others. Thus, the GLS test asymptotically controls the type 1 error rate under the strong null hypothesis only if all weights are non-negative. When re-analysing the ABBV-8E12 trial, we observed a negative weight for one item and therefore additionally considered a modified GLS test, where we dropped this item (see section “Reanalysis of the ABBV-8E12 trial”). Following the recommendation of^23^, for the OLS and GLs tests we used a modified approximation of the degrees of freedom (df) setting $df=0.5(2 n-3)(1+1/m^2)$, where *n* is the per-group sample size and *m* the number of items. This has been shown to provide better control of the type I error rate for smaller sample sizes (see Supplementary Material A, Subsection 3.1) compared to the original proposal by O’Brien.

#### Bonferroni test

 An alternative approach to computing cumulative test statistics, based on individual tests for each item as the OLS or GLS test, is to consider the results of each of the individual tests separately and to apply a multiplicity adjustment to control the FWER. Individual t-statistics (and the associated un-adjusted p-values) are obtained from multiple marginal models as described above. Then an adjusted significance level is applied. The overall null hypothesis of no treatment effect in any item, is rejected if the smallest p-value across all items falls below the Bonferroni adjusted significant level (which is $\alpha /10$, as 10 items are considered). An advantage of this approach is that it also provides a test to compare the item scores for each item: One can reject the null hypothesis of no treatment effect for an item, if the corresponding p-value falls below the Bonferroni adjusted significance level. A further improvement for the individual tests can be obtained by applying the Bonferroni-Holm test.

#### Hommel/Simes test

 Similar to the Bonferroni test, also other multiple testing procedures can be applied to test the overall null hypothesis. An example is the Simes^24^ test for the global null hypothesis of no treatment effect in any item (and its closure the Hommel test^25^, to obtain tests for the individual items). This test is, for example, implemented in the R package hommel. For these tests control of the type I error rate has been shown for independent test statistics as well as test statistics with certain positive correlation structures.

#### Omnibus test

 The Omnibus test^26^ is an alternative test for the global null hypothesis of no treatment effect in any item which is based on individual test statistics. This test has shown to have robust power both in settings where for a few or for many null hypotheses the alternative hypothesis holds false. It is based on cumulative sums of (reciprocal) transformed sorted p-values. Note that theoretical type I error rate control for these tests has been shown for independent test statistics only.

#### Omnibus test for domain scores

 The 10 items of the PSPRS-10 are divided into three domains (see Table 1). For this test we first compute the sum across item scores for each domain. We then perform the ANCOVA analysis for each of the resulting three domain scores and then adjust for multiplicity using the Omnibus test as above.

#### MaxT test

 The Bonferroni test rejects if the minimum of the individual p-values is smaller than the Bonferroni-adjusted critical value. It is strictly conservative as it does not take into account the correlation of test statistics. If correlations are taken into account, a more powerful test can be constructed, that uniformly improves the Bonferroni test. We consider a test based on individual t-test statistics for the different item scores, computed from separate ANCOVA models. The test is based on the maximum of the individual test statistics, and the null distribution of the resulting maximum test statistics is computed based on the estimated correlation matrix of the individual test statistics.

To improve the accuracy of the subsequent normal approximation, instead of t-statistics, we first replace the t-values with transformed z-values to take into account the degrees of freedom, setting

$$z_i = \Phi ^{-1}(F_{t,df}(-t_i)), \quad i = 1, \dots , 10,$$

where $\Phi ^{-1}$ and $F_{t,df}$ denote the quantile function of the normal distribution and the CDF of the t-distribution, respectively.

Since low values of the item scores correspond to a better outcome, small (negative) t-values or equivalently large (positive) z-values, as defined above, indicate a beneficial treatment effect. Therefore, the test statistics to test the overall null hypothesis of no (beneficial) treatment effect in any item, is defined as the maximum of the z-statistics. Then, the multiplicity adjusted p-value is obtained, based on the distribution function of the multivariate normal distribution and is given by $P_{\varvec{Z}_{\max }}=1-P_{\varvec{\Sigma }}(\varvec{Z}\le \varvec{z}_{\max })$, $\varvec{Z}\sim \mathcal {N}(\varvec{0},\,\hat{\varvec{\Sigma }})\,$ where $\varvec{z}_{\max }=\left( z_{\max },\dots ,z_{\max }\right)$, $z_{\max }=\max (z_1,\dots ,z_{10})$ and the correlation matrix $\hat{\varvec{R}}$ is estimated using multiple marginal models as above (see the paragraph on OLS and GLS tests). As this test depends on the estimated correlation matrix, it controls the type 1 error rate only asymptotically. For sample sizes going to infinity, the estimated correlations converge almost surely to the actual correlations, making the null distribution estimates increasingly accurate.

The considered analysis methods are summarised in Table 2.

**Table 2:** The considered analysis methods: category of the method (first column), name of the analysis method (second column), short name used to label the method in the result section (third column), and description for each test (fourth column). Each of the methods is applied to the original scoring and the PSPRS-10 scoring..

Category	Name	Abbreviation	Description
Univariate test statistic (of aggregated scores )	PSPRS-10 Sum Scale	PSPRS-10	Sum of the item scores is used as the dependent variable.
	IRT-based test	IRT.PSIF	IRT-based estimate of the latent variable is used as the endpoint.
	Linear model-based test	LM.PSIBPF	uses a linear model-based estimate of the latent variable, with a weighted sum of the item scores, as the endpoint.
Weighted test statistic composed of that of the individual items.	O’Brien’s OLS test	OLS	sum of t-statistics of the individual items with equal weights (see Supplementary Material A, Subsection 3.1).
	O’Brien’s GLS test	GLS	sum of t-statistics of the individual items with unequal weights (see Supplementary Material A, Subsection 3.1).
	O’Brien’s GLS test	GLS-26	the GLS test based on 9 items (after eliminating item 26).
Individual (item) test statistic	Bonferroni correction	Bonf	based on the smallest p-value across the individual test statistics of the 10 items.
	maximum T-value	MaxT	based on the largest (transformed) t-value among the 10 items.
	Simes	Simes	based on individual adjusted p-values from the items.
	Omnibus	Omnibus	based on cumulative sums of the transformed sorted p-values when there is no a priori knowledge on the number of false individual null hypotheses.
	Combination test: Omnibus and domain scores	Omnibus-dom	Omnibus test applied to the domain scores, defined as the sums of all item scores within a domain.

## Simulation study

To evaluate the operating characteristics of the considered analysis methods, we conducted a large scale simulation study using three approaches to simulate individual item scores of the FDA-recommended subset of items of the PSPRS. This allows us to assess the robustness of findings with respect to specific assumptions of the data generating process. Especially, we generated data (1) from a discretised multivariate normal distribution, (2) with a Bootstrap approach based on the ABBV-8E12 study, and (3) based on a longitudinal IRT model. As no differences between groups were observed, we used pooled (across treatment groups) estimates of the outcomes distribution and also pooled the groups to generate the Bootstrap samples.

### Simulation of the item level data

Below, we describe the three data simulation approaches in detail. Table 3 summarises the key aspects of the different approaches.

All the simulation methods yielded scores using the original scoring of the ten items. To generate items according to the PSPRS-10 scoring, we collapsed in a further step the corresponding item levels. In the simulations we considered a trial with an experimental treatment and a control arm, assuming a per-group sample size of $n=70$. For each scenario 10.000 trials were simulated.

**Table 3:** Parameter specifications for simulation studies.

Name	Type	Values	Description
Analysis type	Design choice	two covariates	Analysis is based on ANCOVA models including the treatment effect and the relevant baseline value, to the analysis methods summarised in Table 2, as covariates.
Total sample size per treatment	Design choice	70 per treatment group	The sample size is fixed across simulation approaches.
Number of simulations runs		10,000 per simulation approach	The number of simulations runs is fixed across simulation approaches.
Nominal significance level	Design Choice	$\alpha =0.025$	The one-sided nominal significance level is fixed in all analysis methods and across simulation approaches.
Parametric simulation method: discretised item scores	Assumption	effect sizes are either fixed/varied across all items or fixed across domains (details in Table 4)	First, data is generated from the multivariate normal distribution. It is then discretised to the nearest integer and truncated to the range 0 to 4.
Non-parametric simulation approach: Bootstrap (without replacement)	Assumption	effect sizes are either fixed/varied across all items or fixed across domains (details in Table 4)	Data sets are resampled based from the ABBV-8E12 study at baseline and Week 52 (“Methods” Section)
IRT-based simulation method	Assumption	Multiple ratios $\rho$ (see section “Simulation of the item level data”) of the progression equation are investigated: 0.45, 0.50, 0.55, 0.60, 0.65, 0.70, 0.75.	Subjects are sampled from the ABBV-8E12 dataset. Subsequently, corresponding relevant covariates are used to construct the progression equation and generate the IRT-based item level data.

#### Discretised multivariate normal distribution

 To parameterise the simulation distribution, we estimated the $20\times 1$ mean vector and $20\times 20$ covariance matrix of the item scores at baseline and Week 52 from the ABBV-8E12 trial data. Especially, the control mean was estimated based on the data of the placebo group and the co-variances were estimated in each of the three treatment groups and then a pooled estimate, $\hat{\varvec{\Sigma }}_p$, was computed, weighting according to the sample sizes. The mean and variance estimates and the corresponding estimate of the correlation matrix are presented in Supplementary Tables 4–6.

Then, to generate the control group data, we drew vectors from a multivariate normal distribution with mean $\hat{\varvec{\mu }}_3$ and covariance $\hat{\varvec{\Sigma }}_p)$. Similarly, the experimental treatment groups data was drawn from a multivariate normal distribution with the same covariance matrix but mean $\hat{\varvec{\mu }}_3-\varvec{d}$, where $\varvec{d}$ denotes the vector of effect sizes across items.

The vectors sampled from the normal distributions are then discretised by rounding, and trimming the scores to the range 0 to 4.

To evaluate the generalisability of our results to broader settings, we conducted additional simulations detailed in Section 2 of Supplementary Material A. Specifically, we assessed the impact of sample size by simulating trials with $n=35$ and $n=140$. We also explored scenarios with strong treatment effects in certain domains or items, and lower effects in others. Furthermore, to examine the impact of the assumed correlation structure, we performed simulations with various correlation scenarios, assuming equal correlation for items within a domain and a possibly lower, equal correlation between domains.

#### Bootstrap

 We resampled subjects from the ABBV-8E12 study and considered both, resampling with and without replacement, but report here only the results of the latter as both provided similar results. We resampled patients for the treatment and control groups from the complete cases (where all items at the baseline and Week 52 visit were available) pooled across all three treatment groups. Note that by sampling subjects rather than individual item scores, we preserved the correlation between item scores as well as the correlation between baseline and follow-up measurements. For simulations under the alternative hypothesis, for the subjects sampled in the experimental treatment arms the item scores at Week 52 were adjusted by the assumed treatment effect. Especially, for an effect size $d_k$ and item scores $y_{k,i}$ for item $k=1,\ldots ,10$ and subject $i=1,\ldots , n$ in the experimental treatment group, where *n* is the per group sample size of the simulated sample, we adjust the scores $y_{k,i}$ such that the mean effect in the population (up to floor effects due to the bounded scale) is approximately $d_k$. This is achieved setting

1. For all $k=1,\ldots ,10$ and $i=1,\ldots ,n$, set $y_{k,i}\rightarrow y_{k,i}-\lfloor d_k \rfloor ,$ where $\lfloor d_k \rfloor$ denotes the floor value of the individual item effect size $d_k$.
2. Randomly select a subset of $np_k$ (rounded to the nearest integer) subject indices, denoted by $I_k$, where $p_k=d_k-\lfloor d_k \rfloor$ and set $y_{k,j}\rightarrow y_{k,j}-1$ for all $j\in I$.
3. Finally, trim the scores to the range of the scale, setting $y_{k,i}\rightarrow max(0,y_{k,i})$ for all $k=1,\ldots ,10$ and $i=1,\ldots ,n$.

#### IRT model based data generation

 We simulated item level data for the baseline and Week 52 item scores based on the longitudinal IRT model of^14^ fitted on multiple data sets from a set of interventional trials in PSP. They used a 2-parameter graded response (GR) model (see, e.g., Equation 4 in^17^) based on discrimination and difficulty as item characteristic parameters. The GR model was fitted to the ordered polytomous data, scores of the FDA-selected subset of items (but ignoring the rescoring recommendation) from the PSPRS-10 in all interventional studies. The underlying latent variable at time *t*, in years, is represented through a disease progression model for individual *i*, given by

$$\psi _{i}(t)=\psi _{i}(0)+ s_i t$$

where $\psi _{i}(0)$ and $s_i$ respectively denote the intercept and slope. For a subject *i*, intercept and slope are given as a function of relevant baseline and slope covariates of the patient, as age, sex, and PSP diagnostic phenotype (see^14^ for a detailed description of the derivation of the corresponding prediction model). To model disease progression under the alternative, for patients in the treatment group, we assumed a smaller slope in the longitudinal latent variable model. Especially, we assumed that the latent variable evolves as $\psi _{i}(0)+ \rho s_it$, where $\rho <1$ indicates a beneficial treatment effect.

To simulate the data we sampled subjects from the ABBV-8E12 study and based on the covariate information of the sampled subjects, we computed for each subject the predicted latent variable at baseline and Week 52. Based on these latent variables we then simulate the individual item scores using the items discrimination and difficulty parameter as estimated in^14^.

### Considered treatment effect scenarios

The performance of the different testing approaches depends on the pattern of treatment effects across the items. We therefore considered a range of treatment effect patterns. For the simulations based on discretised multivariate normal samples and the Bootstrap approach, the considered effect size scenarios are given in Table 4 and include the case of small, intermediate, and strong equal absolute effects across items ($\varvec{d}_{1}^\prime ,\varvec{d}_{2}^\prime ,\varvec{d}_{3}^\prime$). Here, $\varvec{d}_{l}^\prime , l=1,\dots ,12$ denote the transposed treatment effect vectors across items. We further considered scenarios ($\varvec{d}_{4}^\prime -\varvec{d}_{9}^\prime )$ where there the treatment effect is limited to items in a single or two domains. Within each domain, the absolute treatment effects are assumed to be equal. For instance, in $\varvec{d}_{5}^\prime$ we assume an equal absolute treatment effect in each the FDA-recommended items in the History Domain (items 3, 4 and 5). As a reference, we have also included three scenarios in which the treatment effect is isolated to a single item ($\varvec{d}_{10}^\prime -\varvec{d}_{12}^\prime )$.

For the data generated with the IRT model, we considered different effects on the slope of the latent variable, and specified $\rho$ as $\rho = \lbrace 0.45, 0.5,0.55,0.6,0.65,0.7,0.75 \rbrace$ which leads to power values that allow to discriminate results in the statistical tests with respect to power.

**Table 4:** Effect size scenarios considered in the simulation study.

		Dysp.FS	Use.KF	Fall	Dysa	Dysp.	Neck.Ri	Ari.FC	Gait	Pos.St	Sit
Equal effect size	$\varvec{d}_{1}^\prime$	0.20	0.20	0.20	0.20	0.20	0.20	0.20	0.20	0.20	0.20
Equal effect size	$\varvec{d}_{2}^\prime$	0.25	0.25	0.25	0.25	0.25	0.25	0.25	0.25	0.25	0.25
Equal effect size	$\varvec{d}_{3}^\prime$	0.30	0.30	0.30	0.30	0.30	0.30	0.30	0.30	0.30	0.30
History domain	$\varvec{d}_{4}^\prime$	0.85	0.85	0.85	0.00	0.00	0.00	0.00	0.00	0.00	0.00
Bulbar exam	$\varvec{d}_{5}^\prime$	0.00	0.00	0.00	1.25	1.25	0.00	0.00	0.00	0.00	0.00
Gait/midline exam	$\varvec{d}_{6}^\prime$	0.00	0.00	0.00	0.00	0.00	0.50	0.50	0.50	0.50	0.50
History domain & Bulbar exam	$\varvec{d}_{7}^\prime$	0.50	0.50	0.50	0.50	0.50	0.00	0.00	0.00	0.00	0.00
History domain & Gait/midline exam	$\varvec{d}_{8}^\prime$	0.30	0.30	0.30	0.00	0.00	0.30	0.30	0.30	0.30	0.30
Bulbar & Gait/midline exam	$\varvec{d}_{9}^\prime$	0.00	0.00	0.00	0.35	0.35	0.35	0.35	0.35	0.35	0.35
Dysphagia for solids (in History domain)	$\varvec{d}_{10}^\prime$	2.50	0.00	0.00	0.00	0.00	0.00	0.00	0.00	0.00	0.00
Dysarthria (in Bulbar exam)	$\varvec{d}_{11}^\prime$	0.00	0.00	0.00	2.50	0.00	0.00	0.00	0.00	0.00	0.00
Neck rigidity (in Gait/midline exam)	$\varvec{d}_{12}^\prime$	0.00	0.00	0.00	0.00	0.00	2.50	0.00	0.00	0.00	0.00

The rows correspond to vectors $\varvec{d}$ of effect sizes for the 10 items in the PSPRS-10.

### Results of the simulation study

In the simulation study, we assessed the type I error rate and power of each of the analysis methods for the different data generating procedures and treatment effect scenarios.

**Fig. 1:**
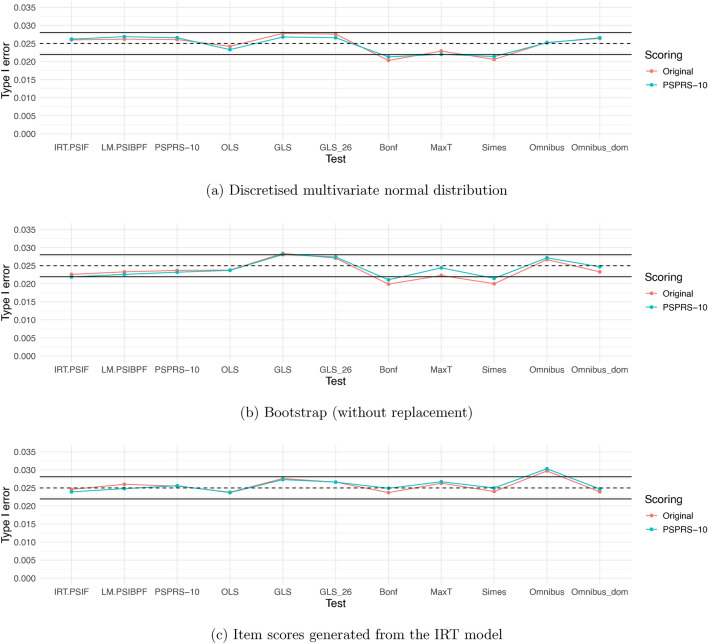
Type I error rates of the hypothesis tests for the different simulation approaches. The dashed lines represent the nominal significance level of 0.025 (one-sided). The black solid lines represent the 95% prediction limits (0.02194, 0.02806) for the estimated type I error rate from 10,000 simulation runs when the actual type I error is 0.025.

The simulated type 1 error rates indicate control of the type 1 error rate for all methods in the considered scenarios with two exceptions: The Omnibus test in the case of the simulations based on the IRT model and the GLS tests which showed some inflation of the type I error rate (see Fig. 1).

**Fig. 2:**
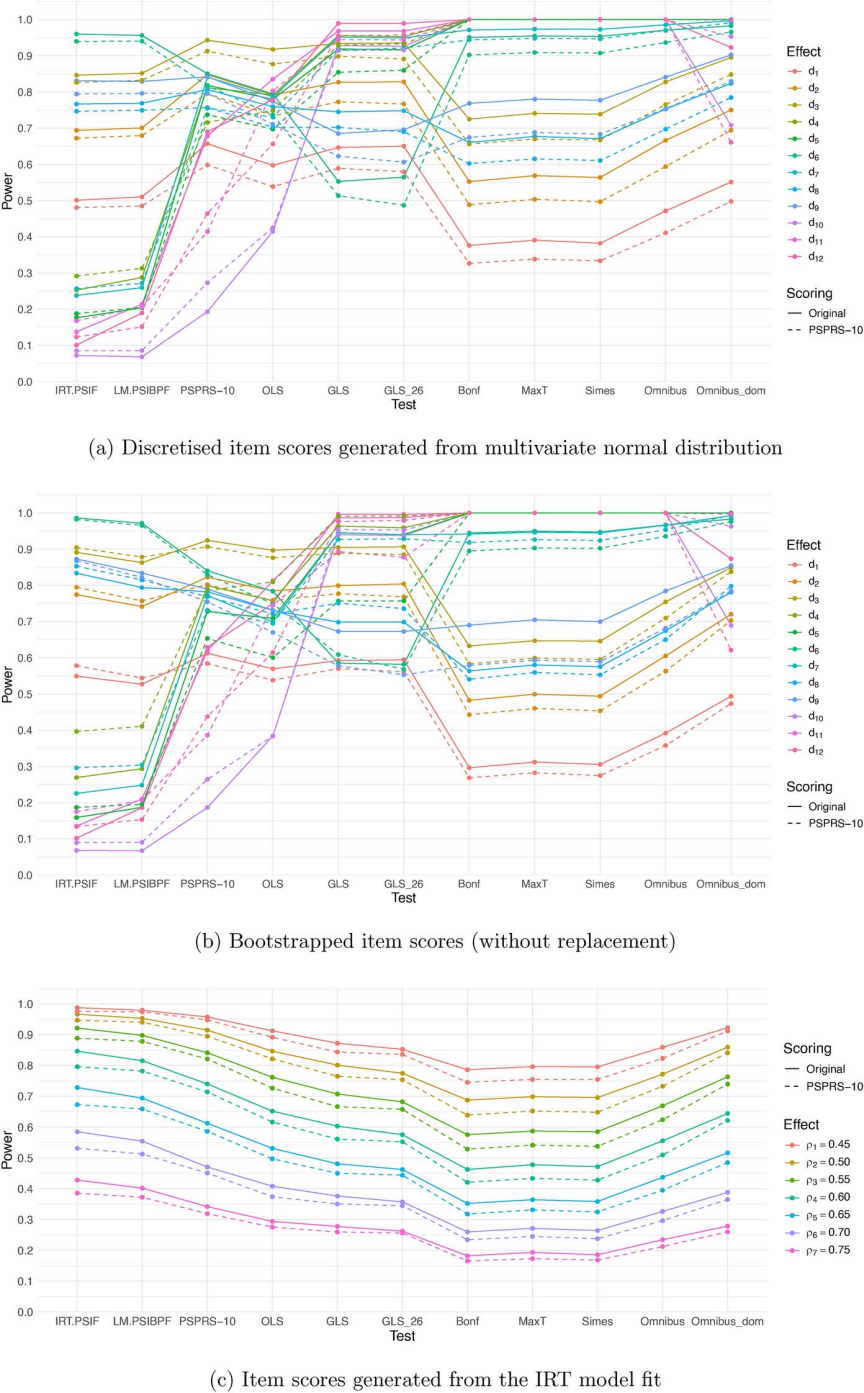
Power of the considered testing procedures for all simulation approaches and effect size scenarios.

Figure 2 summarises the power of the different methods across all simulation approaches and effect size scenarios, both for the original scoring of the items as well as the FDA-PSPRS-10 scoring. Based on 10000 simulations runs, the standard error of the power estimates is bounded from above by 0.005. To better distinguish the scenarios, separate plots for different treatment effect scenarios and data simulation approaches are provided in Supplementary Figs. 1 and 2. Additionally, Supplementary Tables 1–3 present simulated power values of the different methods across all simulation approaches and effect size scenarios, both for the original scoring of the items as well as the FDA-PSPRS-10 scoring.

#### Comparison of testing procedures

In treatment effect scenarios where there is a homogeneous absolute effect in all items (Scenarios $\varvec{d}_{1}^\prime$, $\varvec{d}_{2}^\prime$ and $\varvec{d}_{3}^\prime$) we observe the highest power for the test based on the classical PSPRS-10 using the original scoring. This holds for data simulated with Bootstrap as well as data simulated from the multivariate normal distribution. Similarly, the GLS test and its modification excluding item 26 (Gait) show high power values. However, even after eliminating this item, we observed negative weights for other items in simulated data sets, which causes issues in interpretation. In scenarios with homogeneous effects, also the test using the latent variable estimate from the IRT model as well as its approximation (with slightly lower power) and the Omnibus test based on the domain sum score show a good performance. In contrast, the other tests based on multiple testing procedures had substantially lower power.

On the other hand, when there is an effect only in certain domains or individual items, the power of tests based on multiplicity-adjusted separate tests is the highest. Here, the power of the tests based on the PSPRS-10 (both with the original and the PSPRS-10 scoring) is much lower. The large power of the multiple testing procedures in these scenarios is due to the fact that the individual effect sizes for items where there is an effect are large, such that even after multiplicity adjustment, the power is close to 1. The lower power of the PSPRS-10 is due to the dilution of the treatment effect by summing the scores of all items, even though there is a treatment effect in only a few. In the additional simulations, we have included scenarios with lower effect sizes that allow distinguishing the power between the different multiple testing approaches in this scenario.

The tests based on the IRT model have low power if there is an effect in a single domain or single item only. In the considered scenarios where the effect is in two domains, their power was higher than with the PSPRS-10. The Omnibus-dom test has a large power in scenarios where the effect size is homogeneous within one or more domains, but lower power than the other multiple testing procedures if the effect is in a single item only.

When simulating the data using the longitudinal IRT model, for all considered effect sizes (determining the change of the slope of the latent variable), the analysis based on the IRT model performed best in terms of power, followed by the approximate IRT model based on the weighted sum of the item scores. In these settings, also the PSPRS-10, the OLS test and the Omnibus_dom test showed a good performance. Note that in the IRT model, the treatment effect is modelled directly by assuming different time trends in the latent variable, which results in a treatment effect in all items.

#### Original scoring versus PSPRS-10 scoring

 For most of the tests we observe that the PSPRS-10 scoring causes a drop in the simulated power, specifically in scenarios where there is a homogeneous effect in all items (Scenarios $\varvec{d}_{1}^\prime$, $\varvec{d}_{2}^\prime$ and $\varvec{d}_{3}^\prime$). This might be due to a loss of information by collapsing item levels. For the analysis method based on the IRT models, however, there are also scenarios where the treatment effect is in single domains only, where the power appears to increase after re-scoring. In the simulations based on the IRT model there is a tendency for a decrease in power with PSPRS-10 scoring.

#### Additional simulation scenarios

 In the additional simulation scenarios based on the multivariate normal distribution described in Supplementary Material A, Section 2, we observed that assuming some effect in all items, with a stronger effect in selected domains or items, leads to some improvement in power, especially for the IRT-based test and the PSPRS-10-based tests, with both scorings. As expected, increasing the correlation between baseline and follow-up measurements leads to an increase in power, as the residual variance in the ANCOVA model decreases. However, increasing the correlation between the item scores generally leads to a decrease in power for all methods. For the PSPRS-10, for example, this is due to the fact that the variance of the sum score increases with the between-item correlation. Regarding the type 1 error rate, we observed in the additional simulations that for some correlation structures or for the scenario with the lower sample size of $n=35$, the GLS test showed an inflated type 1 error rate. The Omnibus test also showed type 1 error rates above the nominal level in some scenarios with large correlations while the simulated type 1 error rate of the Omnibus-dom test did not indicate an inflation of the type 1 error rate in these settings.

**Fig. 3:**
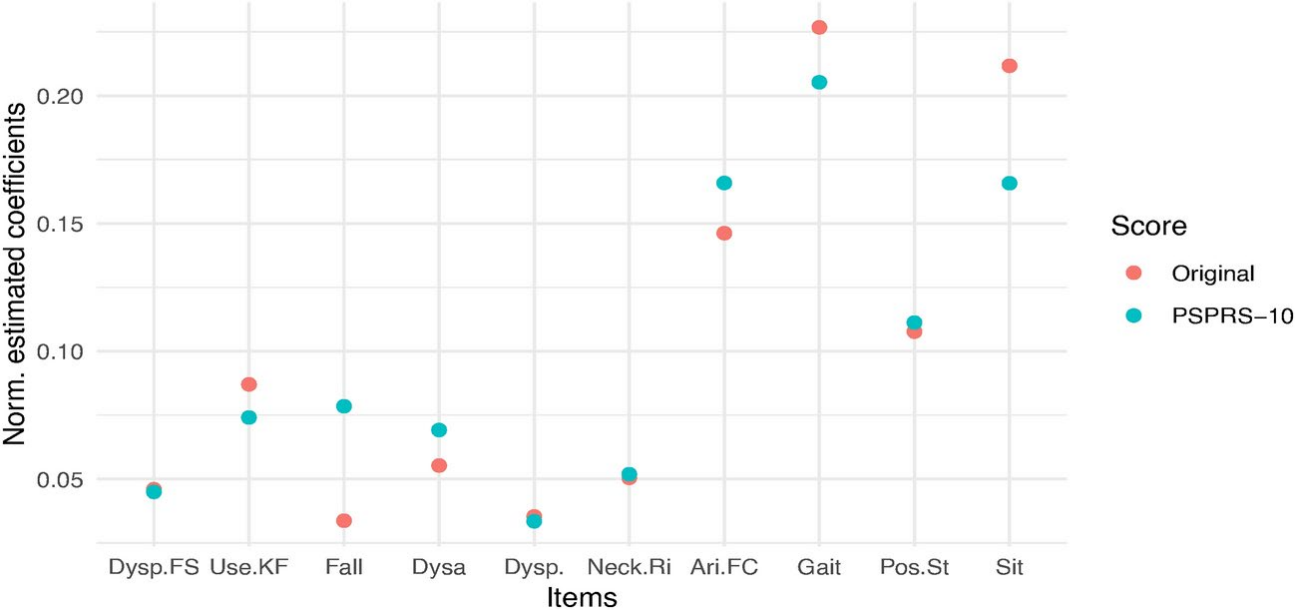
Scatterplot of the transformed estimated latent traits from the IRT model fit and the fitted values from the linear model of items.

**Fig. 4:**
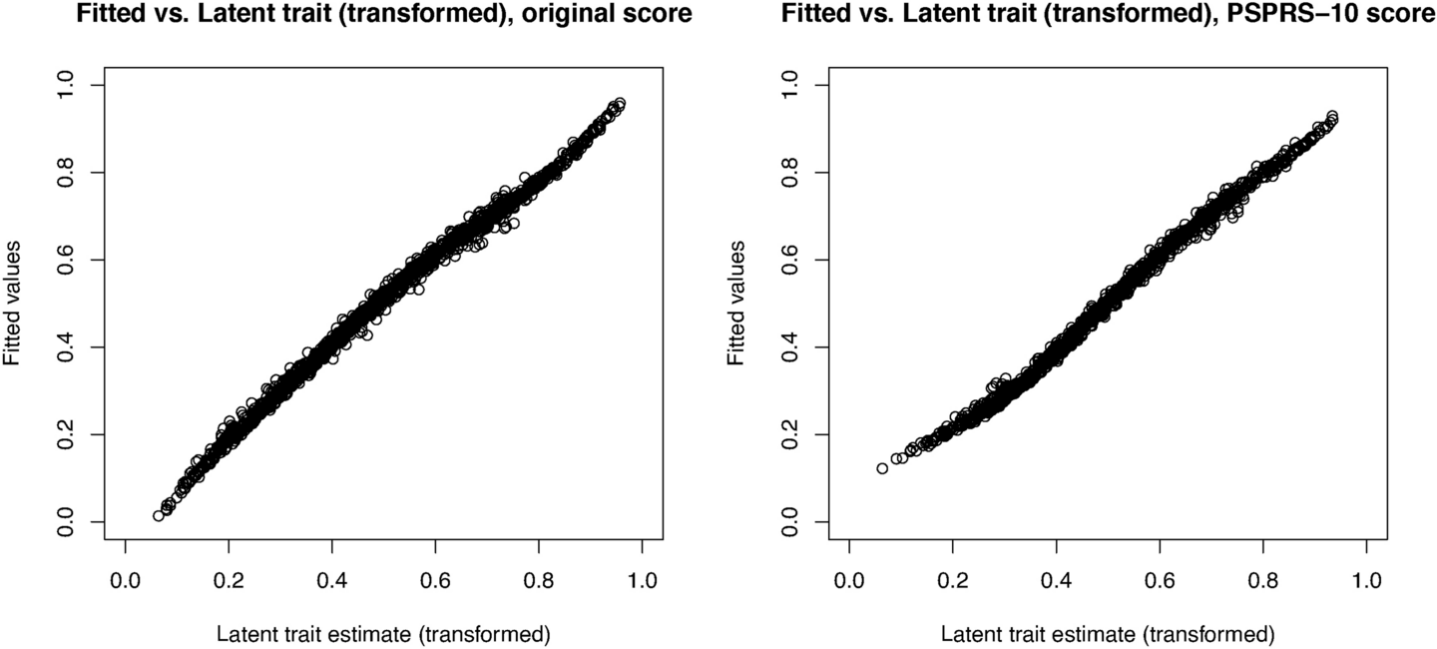
Linear model fit of the latent variable. The plot shows the normalised weights (summing to one) for the items with the original (red) and the FDA-recommended PSPRS-10 scoring (blue).

## Reanalysis of the ABBV-8E12 trial

To illustrate the application of the different analysis methods, we re-analyse the ABBV-8E12 trial with the analysis methods considered above, and compare each dose (tilavonemab 2000, tilavonemab 4000) to placebo, in separate analyses. We included 66, 71, and 64 patients in the tilavonemab 2000, tilavonemab 4000, placebo groups respectively, for which both the baseline and the Week 52 data were available for all the FDA subset of items. Note that in this data set there is a substantial proportion of missing values of item scores in later visits, specifically at Week 52, compared to baseline. This is due to the fact that the whole study was terminated early as no differences between the dose groups compared to placebo were observed in an interim analysis. Supplementary Tables 21 and 22 show the descriptive statistics of the baseline and Week 52 item scores as well as the treatment effect estimates and corresponding p-values from marginal ANCOVA models with the Week 52 scores as dependent variable and the baseline score and treatment as independent variables respectively for the original item scoring and the FDA PSPRS-10 scoring.

Results of the different hypotheses tests defined in section “Analysis methods” are given in Supplementary Table 23. While, in agreement to the original analysis, none of them indicates a significant difference between groups, we find that the testing procedures yield a wide range of p-values. This reflects the fact, that the different tests focus on different aspects of deviations from the null hypothesis.

To assess the latent variable estimates obtained by a weighted sum of the item scores used in the approximate IRT-based test (see section “Analysis methods”) we visually inspected the model fit, both, for the linear model fitted to the original and the PSPRS-10 items. Especially, we plot the latent variable estimate based on the linear model fit against the estimates obtained with the EAP method in the ABBV-8E12 data set, pooling the data from all visits and treatment groups as described above (see Fig. 3). The figure shows that, when computing the latent variables based on the original item scoring, we observe a nearly perfect model fit with minimal errors. Estimating the latent variable based on a weighted sum of the PSPRS-10 items yields a somewhat larger error, especially for small values of the latent trait. The weights of the items in the resulting linear models are depicted in Fig. 4 with the largest weights allocated to the items ’arising from chair’, ’gait’, ’postural stability’ and ’sitting down’ from the gait/midline exam domain.

**Fig. 5:**
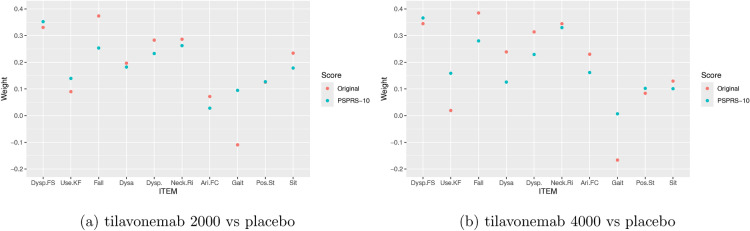
Weights of the individual test statistics in the GLS test in the re-analysis of the ABBV-8E12 trial.

The weights for the individual t-statistics in the GLS test are given in Fig. 5. For the original scoring, the weight of item 26 (Gait) becomes negative. Using the FDA PSPRS-10 scoring, we observed no negative weights.

Note that in this illustrative example we estimated the IRT model from the same clinical trial for which the model is applied to compute the endpoint. In principle, the resulting dependence of the model estimate with the clinical trial data may introduce a bias. Even though it is expected that this bias is not substantial, as the fit of the IRT model does not use treatment label information, estimating the IRT model from an independent data set, avoids this issue. Further simulations are needed to provide a detailed estimate of the impact of using the same data set to fit the IRT model.

## Discussion

In this paper, we assessed a range of testing approaches to compare treatment groups in multivariate endpoints in a simulation study in the setting of PSP. We investigated multiple data generation approaches and effect size scenarios to assess the robustness of findings. Besides classical approaches to test multivariate endpoints, we also considered tests based on estimates of the latent variables computed from an IRT model which represent the patients’ disease status. Additionally, we considered an approximate version of the corresponding outcome variable which is defined as a weighted average of the individual item scores and fits the IRT based estimate surprisingly well.

This study has several limitations. First, we assumed that the clinical trial has no missing values or dropouts. Dropouts can lead to lower power, due to a lower sample size, but they also can introduce bias if the distribution of the observed outcomes differs from the distribution of the unobserved values, which are missing. Because of repeated measurements of the PSPRS items, approaches using early outcome variables can be used to account for missing data, e.g., based on mixed models that model the course of the disease. In future work the proposed analysis approaches can be extended to such models. Vickerstaff et al.^27^ assessed a number of approaches to analyse multiple correlated outcomes focusing on settings with missing data, limiting the analysis, however, to the case of two endpoints only. Several alternative methods, which have not been considered in this study, exist. For example, analysis approaches based on factor analyses^28^ have been proposed, which can be used to derive weighted scores similar to the approximate IRT-based test considered here. Additionally, for each of the considered analysis methods, besides the baseline PSPRS item scores, sum score, or baseline latent variable estimate, other covariates such as the baseline progression rate, study site or region, sex, or age could be adjusted for in an ANCOVA. Another option to analyse multiple item scores is multivariate regression with multiple dependent variables. The modified 10-item PSPRS considered in this manuscript was recommended to a pharmaceutical company in a pre-IND meeting. However, there is no generally FDA-approved version of the PSPRS. Instead, the FDA has informally indicated that each PSP trial sponsor must have a pre-IND meeting where the FDA will provide a PSPRS modification specific to that trial. The findings on the specific PSPRS-10 considered in this paper may not generalise to other such modifications.

We utilised simulation approaches based on a discretised multivariate normal distribution and Bootstrap, both flexible tools for simulating data across diverse effect size scenarios, including uniform and heterogeneous item-level and domain-wise effect sizes. Our third approach utilised a longitudinal IRT model, focusing on the effect on the underlying latent variable. These approaches are particularly relevant for calculating power based on effect size assumptions for specific domains or items in the 10-item version of the PSPRS.

The currently used PSPRS scores based on sums of item scores are interpretable composite endpoints, and the equal weighting of the item scores makes them easy to use. Their historical use also enables comparisons to past trials. However, these scores may lack sensitivity if there is an effect in only some items. They also do not explicitly account for the correlation between items and implicitly assume additive and equal contributions of the item scores to the measure of the patient’s disease status. The heterogeneous item weights obtained from the approximate IRT analysis indicate that items do not equally contribute to the latent variable estimated by the IRT model, which is a measure of the patient’s disease status. By re-weighting the item scores, the resulting modified sum score (used in the approximate IRT-based test) has a high correlation with the latent variable. Interestingly, the items receiving a large weight correspond to items with high discriminative power in the IRT model investigated in^14^. While the latent variable estimate from the IRT model provided slightly higher power than the weighted sum score used in the approximate IRT test, the latter is more transparent and simpler to calculate, as the contribution of each item score is apparent by the size of the weight. Other tests, including the GLS and OLS tests, weight the scores very differently. Furthermore, as discussed in section “Analysis methods”, the GLS test can be very liberal, especially with small sample sizes, and can lead to negative weights. Therefore, the general guidance has been to use the OLS test instead (see Section 4.4 of^22^). Of note, O’Brien^10^ proposed a rank sum test which could also be considered.

The simulation study demonstrated that none of the considered testing procedures is uniformly optimal but the most powerful test depends on the specific configuration of effect sizes and the data generating mechanism. The test based on the PSPRS-10 performed well in scenarios where there is an homogeneous effect across items. The OLS and GLS tests are tailored to settings where there is a homogeneous effect size across the individual endpoints. Therefore, these tests showed high power in scenarios $d_1$ through $d_3$, where there is an effect in all of the items. Also in settings where there is an effect in some of the domains only, it still provides moderate power. It is reiterated that the GLS test can be liberal and therefore the OLS test might be preferred in these scenarios. Similarly, the GLS, OLS, the Omnibus tests and the tests based on item response models have a larger power than the tests based on multiplicity adjustments of marginal tests. In treatment effect scenarios, where the treatment effect is limited to few domains or items, tests based on multiple testing procedures have a higher power. Also the Omnibus tests have a high power in these settings, however, for those type I error control has only been shown for independent test statistics. Under dependence type 1 error control may be lost and has to be assessed by simulations. Excluding the GLS and omnibus tests which can be liberal, the OLS test maximises the minimal power across the alternatives $d_1-d_{12}$ in the simulations based on the multivariate normal distribution and the bootstrap method.

When simulating the data based on an IRT model, the IRT based analysis appears to be the most powerful. This is not unexpected, as it is the correct model in this case. Another observation is that, for most tests and scenarios, the FDA recommended scoring of the items causes a reduction in the simulated power.

Because all tests, with the exception of the IRT based test, test the strong null hypothesis (see section “Analysis methods”), they can be extended to multiple tests using the closed testing principle. Such multiple tests test for each item the individual null hypotheses that the expected score is larger or equal under treatment than under control controlling the FWER in the strong sense. Rejection of an individual null hypothesis not only allows to conclude that there is an effect in any item (as follows from rejection of the strong null hypothesis), but also to identify in which item the effect is. Tests based on the PSPRS-10 or the approximate IRT-based test allow for a conclusion on the (weighted) average of the item scores, which may have a clearer clinical interpretation than rejection of the strong null hypothesis.

As the optimal testing procedure in terms of statistical power depends on the specific effect size patterns assumed, identifying plausible treatment effect patterns is crucial when planning a study. For instance, treatments with disease-modifying effects that slow disease progression are expected to impact all items, either uniformly or as modeled by the longitudinal IRT model. Conversely, symptomatic treatments are likely to influence certain items or domains more selectively. See^29^ for examples of corresponding treatment candidates. Given scenarios with assumed treatment effects, simulation studies can guide the selection of the most suitable testing strategy. When there is significant uncertainty regarding the treatment effect pattern, a maximin criterion can be applied to choose methods that offer the highest minimum power across all plausible scenarios.

In conclusion, our study underscores that there is no one-size-fits-all testing procedure for evaluating treatment effects using PSPRS item scores; the optimal method varies based on the specific effect size patterns. The efficiency of the PSPRS-10, while generally robust and straightforward to apply, varies depending on the specific patterns of effect sizes encountered and more powerful alternatives are available in specific settings.

## Supplementary Information

Supplementary Information 1.

Supplementary Information 2.

## Data Availability

The data that support the findings of this study are available from Vivli - Center for Global Clinical Research Data at https://vivli.org/ but restrictions apply to the availability of these data, which were used under license for the current study, and so are not publicly available.

## References

[1] U.S. Department of Health and Human Services Food and Drug Administration, “Multiple endpoints in clinical trials guidance for industry draft guidance,” (2017). https://www.fda.gov/downloads/drugs/guidancecomplianceregulatoryinformation/guidances/ucm536750.pdf.

[2] Golbe, L. I. & Ohman-Strickland, P. A. A clinical rating scale for progressive supranuclear palsy. *Brain* **130**(6), 1552–1565 (2007). 17405767 10.1093/brain/awm032

[3] Boxer, A. L. et al. Davunetide in patients with progressive supranuclear palsy: A randomised, double-blind, placebo-controlled phase 2/3 trial. *Lancet Neurol.* **13**(7), 676–685 (2014). 24873720 10.1016/S1474-4422(14)70088-2 PMC4129545

[4] Tolosa, E. et al. A phase 2 trial of the GSK-3 inhibitor tideglusib in progressive supranuclear palsy. *Mov. Disord.* **29**(4), 470–478 (2014). 24532007 10.1002/mds.25824

[5] Nuebling, G. et al. Prospera: A randomized, controlled trial evaluating rasagiline in progressive supranuclear palsy. *J. Neurol.* **263**(8), 1565–1574 (2016). 27230855 10.1007/s00415-016-8169-1

[6] Dam, T. et al. Safety and efficacy of anti-tau monoclonal antibody gosuranemab in progressive supranuclear palsy: A phase 2, randomized, placebo-controlled trial. *Nat. Med.* **27**(8), 1451–1457 (2021). 34385707 10.1038/s41591-021-01455-x

[7] Höglinger, G. U. et al. Safety and efficacy of tilavonemab in progressive supranuclear palsy: A phase 2, randomised, placebo-controlled trial. *Lancet Neurol.* **20**(3), 182–192 (2021). 33609476 10.1016/S1474-4422(20)30489-0

[8] Roesler, T. W. et al. Four-repeat tauopathies. *Prog. Neurobiol.* **180**, 101644 (2019). 31238088 10.1016/j.pneurobio.2019.101644

[9] Levin, J., Kurz, A., Arzberger, T., Giese, A. & Höglinger, G. U. The differential diagnosis and treatment of atypical parkinsonism. *Deutsches Ä rzteblatt Int.* **113**(5), 61 (2016). 10.3238/arztebl.2016.0061 PMC4782269 26900156

[10] O’Brien, P. C. Procedures for comparing samples with multiple endpoints. *Biometrics* **40**(4), 1079–1087 (1984). 6534410

[11] Reitmeir, P. & Wassmer, G. One-sided multiple endpoint testing in two-sample comparisons. *Commun. Stat.-Simul. Comput.* **25**(1), 99–117 (1996).

[12] Ristl, R., Urach, S., Rosenkranz, G. & Posch, M. Methods for the analysis of multiple endpoints in small populations: A review. *J. Biopharm. Stat.* **29**(1), 1–29 (2019). 29985752 10.1080/10543406.2018.1489402

[13] Rasch, G. *Studies in Mathematical Psychology: I. Probabilistic Models for Some Intelligence and Attainment tests* (1960).

[14] Gewily, M. et al. Quantitative comparisons of progressive supranuclear palsy rating scale versions using item response theory. *Mov. Disord.*[SPACE]10.1002/mds.30001 (2024). 39206961 10.1002/mds.30001 PMC11657017

[15] Senn, S. Change from baseline and analysis of covariance revisited. *Stat. Med.* **25**(24), 4334–4344 (2006). 16921578 10.1002/sim.2682

[16] Siemons, L. & Krishnan, E. A short tutorial on item response theory in rheumatology. *Clin. Exp. Rheumatol.* **32**(4), 581–586 (2014). 25065775

[17] Ueckert, S. Modeling composite assessment data using item response theory. *CPT: Pharmacomet. Syst. Pharmacol.* **7**(4), 205–218 (2018). 10.1002/psp4.12280 PMC5915608 29493119

[18] Thissen, D., Pommerich, M., Billeaud, K. & Williams, V. S. Item response theory for scores on tests including polytomous items with ordered responses. *Appl. Psychol. Measurement* **19**(1), 39–49 (1995).

[19] Chalmers, R. P. Mirt: A multidimensional item response theory package for the r environment. *J. Stat. Softw.* **48**, 1–29 (2012).

[20] Pipper, C. B., Ritz, C. & Bisgaard, H. A versatile method for confirmatory evaluation of the effects of a covariate in multiple models. *J. R. Stat. Soc. Ser. C: Appl. Stat.* **61**(2), 315–326 (2012).

[21] Dallow, N. S., Leonov, S. L. & Roger, J. H. Practical usage of O’Brien’s OLS and GLS statistics in clinical trials. *Pharm. Stat.* **7**(1), 53–68 (2008). 17390306 10.1002/pst.268

[22] Dmitrienko, A., Tamhane, A. C. & Bretz, F. *Multiple Testing Problems in Pharmaceutical Statistics* (CRC Press, 2009).

[23] Logan, B. R. & Tamhane, A. C. On O’Brien’s OLS and GLS tests for multiple endpoints. *Lect. Notes-Monogr. Ser.* **47**, 76–88 (2004).

[24] Simes, R. J. An improved bonferroni procedure for multiple tests of significance. *Biometrika* **73**(3), 751–754 (1986).

[25] Hommel, G. A stagewise rejective multiple test procedure based on a modified Bonferroni test. *Biometrika* **75**(2), 383–386 (1988).

[26] Futschik, A., Taus, T. & Zehetmayer, S. An omnibus test for the global null hypothesis. *Stat. Methods Med. Res.* **28**(8), 2292–2304 (2019). 29635962 10.1177/0962280218768326 PMC6676337

[27] Vickerstaff, V., Ambler, G. & Omar, R. Z. A comparison of methods for analysing multiple outcome measures in randomised controlled trials using a simulation study. *Biometr. J.* **63**(3), 599–615 (2021). 10.1002/bimj.201900040 PMC7984364 33314364

[28] Smith, K. W. Forming composite scales and estimating their validity through factor analysis. *Soc. Forces* **53**(2), 168–180 (1974).

[29] Stamelou, M. & Höglinger, G. A review of treatment options for progressive supranuclear palsy. *CNS Drugs* **30**, 629–636 (2016). 27222018 10.1007/s40263-016-0347-2

