# Supplementary material for: Efficiency of multivariate tests in trials in progressive supranuclear palsy
Source: Sci Rep. 2024 Oct 26;14:25581. doi: 10.1038/s41598-024-76668-4 (PMC11514033; doi:10.1038/s41598-024-76668-4)
Supplement: Supplementary file 1 — Supplementary Information 1. [file 41598_2024_76668_MOESM1_ESM.pdf]

Supplementary Material A

September 22, 2024

1 Additional plots and tables of the simulation scenarios described in the main manuscript

Supplementary Table 1: Power of the considered testing procedures for the simulations based on discretised multivariate normal item scores (with the effect size scenarios from Table 4 of the main manuscript). The last row shows the minimum power per analysis test across scenarios. The maximum power per scenario (in each row) is highlighted in bold.

Original scoring

Scenario	IRT.PSIF	LM.PSIBPF	PSPRS-10	OLS	GLS	GLS-26	Bonf	MaxT	Simes	Omnibus	Omnibus-dom
d_1	0.501	0.510	**0.657**	0.597	0.647	0.650	0.376	0.391	0.382	0.472	0.551
d_2	0.694	0.701	**0.848**	0.792	0.827	0.828	0.552	0.569	0.564	0.666	0.750
d_3	0.846	0.852	**0.943**	0.917	0.934	0.935	0.725	0.741	0.738	0.828	0.895
d_4	0.253	0.288	0.811	0.792	0.955	0.953	**1.000**	**1.000**	**1.000**	**1.000**	**1.000**
d_5	0.176	0.204	0.818	0.780	0.919	0.917	**1.000**	**1.000**	**1.000**	**1.000**	**1.000**
d_6	0.960	0.956	0.851	0.795	0.553	0.565	0.951	0.955	0.954	0.971	**0.983**
d_7	0.238	0.260	0.841	0.787	0.951	0.949	0.971	0.974	0.973	0.986	**0.997**
d_8	0.767	0.769	**0.807**	0.759	0.745	0.748	0.661	0.677	0.671	0.753	0.824
d_9	0.831	0.829	**0.842**	0.774	0.685	0.696	0.768	0.780	0.777	0.841	0.902
d_{10}	0.072	0.068	0.193	0.415	0.928	0.926	**1.000**	**1.000**	**1.000**	**1.000**	0.708
d_{11}	0.138	0.214	0.678	0.835	0.968	0.968	**1.000**	**1.000**	**1.000**	**1.000**	**1.000**
d_{12}	0.101	0.189	0.690	0.779	0.990	0.989	**1.000**	**1.000**	**1.000**	**1.000**	0.923
min	0.072	0.068	0.193	0.415	0.553	0.565	0.376	0.391	0.382	0.472	0.551

PSPRS-10 scoring

Scenario	IRT.PSIF	LM.PSIBPF	PSPRS-10	OLS	GLS	GLS-26	Bonf	MaxT	Simes	Omnibus	Omnibus-dom
d_1	0.481	0.485	**0.598**	0.538	0.589	0.579	0.326	0.338	0.334	0.411	0.498
d_2	0.672	0.680	**0.795**	0.734	0.773	0.767	0.488	0.503	0.497	0.594	0.694
d_3	0.826	0.832	**0.913**	0.877	0.898	0.891	0.656	0.670	0.667	0.765	0.848
d_4	0.292	0.313	0.716	0.749	0.929	0.933	**1.000**	**1.000**	**1.000**	**1.000**	**1.000**
d_5	0.188	0.205	0.737	0.697	0.854	0.860	**1.000**	**1.000**	**1.000**	**1.000**	**1.000**
d_6	0.940	0.941	0.817	0.737	0.513	0.487	0.902	0.909	0.907	0.936	**0.966**
d_7	0.257	0.272	0.755	0.730	0.915	0.921	0.944	0.948	0.947	0.970	**0.991**
d_8	0.747	0.749	0.757	0.703	0.702	0.690	0.602	0.615	0.610	0.697	**0.784**
d_9	0.794	0.796	0.796	0.711	0.623	0.606	0.674	0.688	0.683	0.754	**0.829**
d_{10}	0.085	0.086	0.273	0.425	0.914	0.916	**1.000**	**1.000**	**1.000**	**1.000**	0.953
d_{11}	0.168	0.206	0.414	0.803	0.945	0.944	**1.000**	**1.000**	**1.000**	**1.000**	0.998
d_{12}	0.123	0.151	0.464	0.656	0.955	0.957	**1.000**	**1.000**	**1.000**	**1.000**	0.661
min	0.085	0.086	0.273	0.425	**0.513**	0.487	0.326	0.338	0.334	0.411	0.498

Supplementary Table 2: Power of the considered testing procedures for the simulations based on the Bootstrap method. The last row shows the minimum power per analysis test across scenarios. The maximum power per scenario (in each row) is highlighted in bold.

Original scoring

Scenario	IRT.PSIF	LM.PSIBPF	PSPRS-10	OLS	GLS	GLS-26	Bonf	MaxT	Simes	Omnibus	Omnibus-dom
d_1	0.549	0.527	**0.612**	0.569	0.592	0.595	0.296	0.312	0.306	0.392	0.494
d_2	0.774	0.742	**0.823**	0.785	0.799	0.804	0.483	0.500	0.494	0.605	0.720
d_3	0.891	0.863	**0.924**	0.897	0.905	0.906	0.633	0.647	0.646	0.754	0.849
d_4	0.269	0.294	0.799	0.758	0.964	0.959	**1.000**	**1.000**	**1.000**	**1.000**	**1.000**
d_5	0.159	0.187	0.728	0.709	0.945	0.940	**1.000**	**1.000**	**1.000**	**1.000**	**1.000**
d_6	**0.986**	0.972	0.840	0.784	0.586	0.582	0.945	0.950	0.947	0.967	0.983
d_7	0.225	0.248	0.772	0.699	0.946	0.940	0.942	0.947	0.945	0.966	**0.993**
d_8	**0.834**	0.794	0.782	0.731	0.698	0.699	0.564	0.580	0.576	0.675	0.781
d_9	**0.873**	0.834	0.790	0.732	0.673	0.673	0.690	0.705	0.700	0.784	0.855
d_{10}	0.068	0.067	0.186	0.384	0.940	0.938	**1.000**	**1.000**	**1.000**	**1.000**	0.689
d_{11}	0.135	0.210	0.620	0.813	0.986	0.987	**1.000**	**1.000**	**1.000**	**1.000**	**1.000**
d_{12}	0.102	0.186	0.629	0.750	0.997	0.996	**1.000**	**1.000**	**1.000**	**1.000**	0.873
min	0.068	0.067	0.186	0.384	**0.586**	0.582	0.296	0.312	0.306	0.392	0.494

PSPRS-10 scoring

Scenario	IRT.PSIF	LM.PSIBPF	PSPRS-10	OLS	GLS	GLS-26	Bonf	MaxT	Simes	Omnibus	Omnibus-dom
d_1	0.578	0.544	**0.584**	0.538	0.570	0.561	0.269	0.282	0.275	0.358	0.473
d_2	0.795	0.757	**0.802**	0.758	0.777	0.768	0.443	0.460	0.454	0.563	0.703
d_3	0.904	0.878	**0.907**	0.876	0.889	0.885	0.583	0.599	0.595	0.710	0.838
d_4	0.396	0.411	0.786	0.809	0.991	0.992	**1.000**	**1.000**	**1.000**	**1.000**	**1.000**
d_5	0.187	0.195	0.654	0.601	0.757	0.757	**1.000**	**1.000**	**1.000**	**1.000**	**1.000**
d_6	**0.982**	0.966	0.829	0.749	0.609	0.568	0.895	0.903	0.902	0.936	0.976
d_7	0.296	0.304	0.731	0.695	0.927	0.928	0.918	0.926	0.924	0.954	**0.994**
d_8	**0.853**	0.815	0.769	0.721	0.751	0.736	0.541	0.560	0.553	0.650	0.798
d_9	**0.868**	0.822	0.754	0.670	0.578	0.553	0.579	0.593	0.590	0.682	0.784
d_{10}	0.089	0.090	0.264	0.384	0.954	0.953	**1.000**	**1.000**	**1.000**	**1.000**	0.963
d_{11}	0.175	0.207	0.386	0.745	0.893	0.877	**1.000**	**1.000**	**1.000**	**1.000**	0.997
d_{12}	0.134	0.153	0.438	0.615	0.976	0.980	**1.000**	**1.000**	**1.000**	**1.000**	0.621
min	0.089	0.090	0.264	0.384	0.570	0.553	0.269	0.282	0.275	0.358	0.473

Supplementary Table 3: Power of the considered testing procedures for the simulations based on the IRT model fit.

Original scoring

Scenario	IRT.PSIF	LM.PSIBPF	PSPRS-10	OLS	GLS	GLS-26	Bonf	MaxT	Simes	Omnibus	Omnibus-dom
ρ_1	**0.988**	0.980	0.958	0.913	0.872	0.853	0.786	0.796	0.795	0.859	0.923
ρ_2	**0.967**	0.953	0.915	0.847	0.801	0.775	0.688	0.699	0.696	0.772	0.860
ρ_3	**0.921**	0.898	0.842	0.762	0.708	0.682	0.576	0.587	0.585	0.669	0.763
ρ_4	**0.847**	0.816	0.740	0.652	0.603	0.576	0.463	0.478	0.472	0.556	0.644
ρ_5	**0.729**	0.694	0.613	0.531	0.481	0.462	0.352	0.364	0.358	0.437	0.516
ρ_6	**0.585**	0.554	0.471	0.408	0.376	0.357	0.260	0.271	0.264	0.326	0.388
ρ_7	**0.428**	0.402	0.342	0.294	0.278	0.262	0.182	0.193	0.186	0.234	0.279

PSPRS-10 scoring

Scenario	IRT.PSIF	LM.PSIBPF	PSPRS-10	OLS	GLS	GLS-26	Bonf	MaxT	Simes	Omnibus	Omnibus-dom
ρ_1	**0.976**	0.974	0.948	0.892	0.844	0.836	0.746	0.755	0.755	0.823	0.912
ρ_2	**0.947**	0.941	0.895	0.822	0.765	0.754	0.639	0.652	0.648	0.733	0.841
ρ_3	**0.889**	0.878	0.821	0.727	0.666	0.658	0.529	0.541	0.538	0.624	0.740
ρ_4	**0.796**	0.782	0.714	0.617	0.561	0.553	0.421	0.434	0.428	0.509	0.622
ρ_5	**0.673**	0.659	0.586	0.497	0.450	0.444	0.318	0.331	0.325	0.395	0.485
ρ_6	**0.531**	0.513	0.451	0.374	0.350	0.345	0.234	0.245	0.238	0.296	0.365
ρ_7	**0.386**	0.372	0.319	0.276	0.260	0.256	0.166	0.173	0.168	0.212	0.260

Supplementary Table 4: Means of items at Week 52 and baseline.

	Dysp.FS	Use.KF	Fall	Dysa	Dysp.	Neck.Ri	Ari.FC	Gait	Pos.St	Sit
Week 52	0.77	2.23	2.77	1.94	1.55	2.03	2.91	2.48	2.89	2.53
Baseline	0.59	1.58	2.22	1.64	1.05	1.56	2.06	1.81	2.14	1.67

Supplementary Table 5: Variances of items at Week 52 and baseline.

	Dysp.FS	Use.KF	Fall	Dysa	Dysp.	Neck.Ri	Ari.FC	Gait	Pos.St	Sit
Week 52	0.64	1.05	1.55	0.86	1.32	1.12	1.65	0.85	1.27	1.23
Baseline	0.39	0.72	1.15	0.64	0.87	0.77	1.58	0.80	1.25	0.83

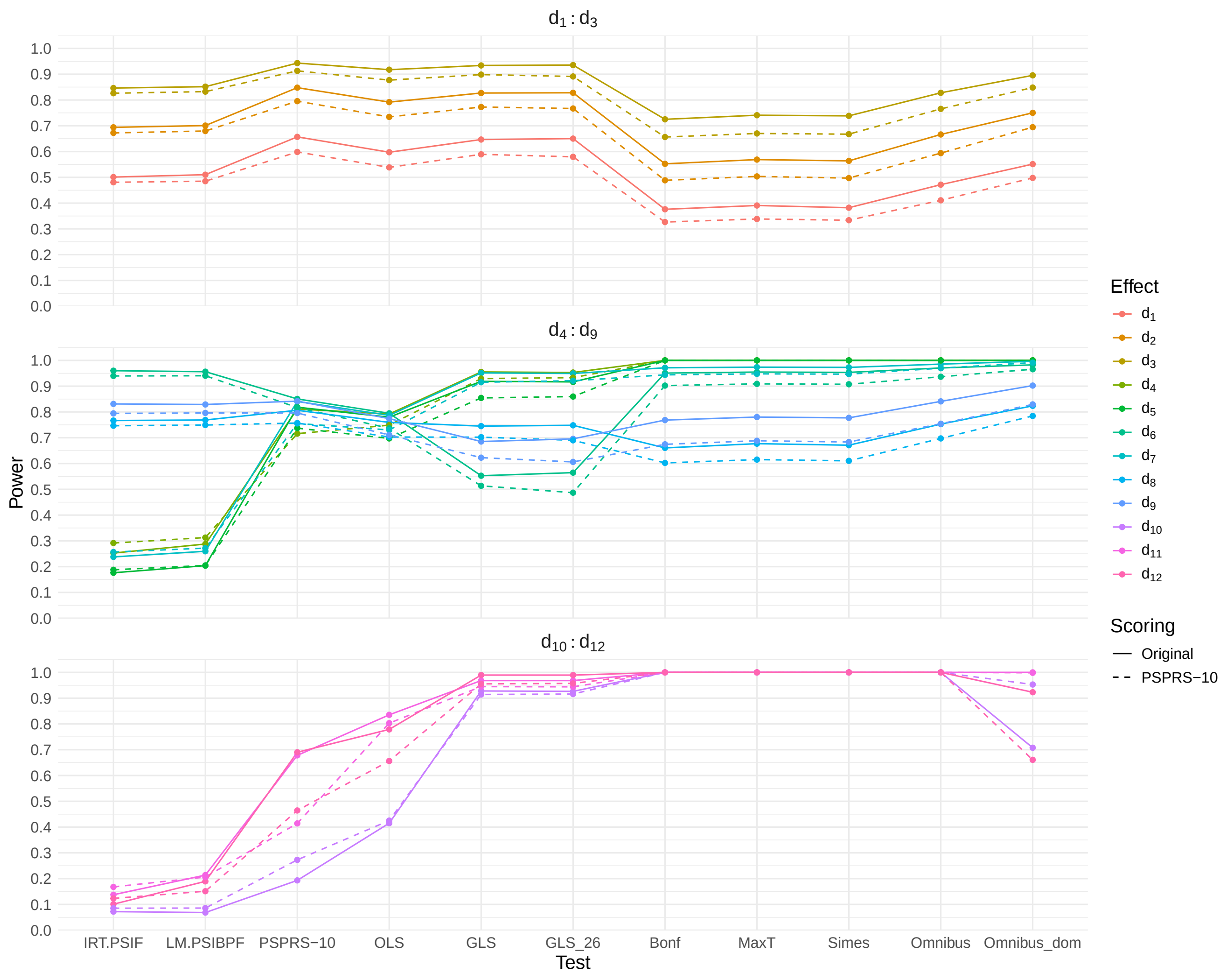

Supplementary Figure 1: Power of the considered testing procedures for the simulations based on discretised multivariate normal item scores (with the effect size scenarios from Table 4 of the main manuscript).

2 Additional simulations based on discretised multivariate normal distribution

2.1 Description of the additional simulation scenarios

Additional sample sizes and effect size scenarios We performed simulations as described in the main manuscript based on the multivariate normal distribution but with per-group sample sizes of $n = 35$ and $n = 140$, using the covariance matrix estimated from the ABBV-8E12 trial data (see Section 4.1 of the main manuscript for more details). Supplementary Tables 4, 5, and 6 show the mean and variance vectors, and the correlation matrix of the ABBV-8E12 trial data at week 52 and at baseline, respectively. For $n = 140$, the effect sizes from Table 4 of the main manuscript are rescaled by multiplying them by a scaling parameter $c = \sqrt{70}/\sqrt{140}$, so that the

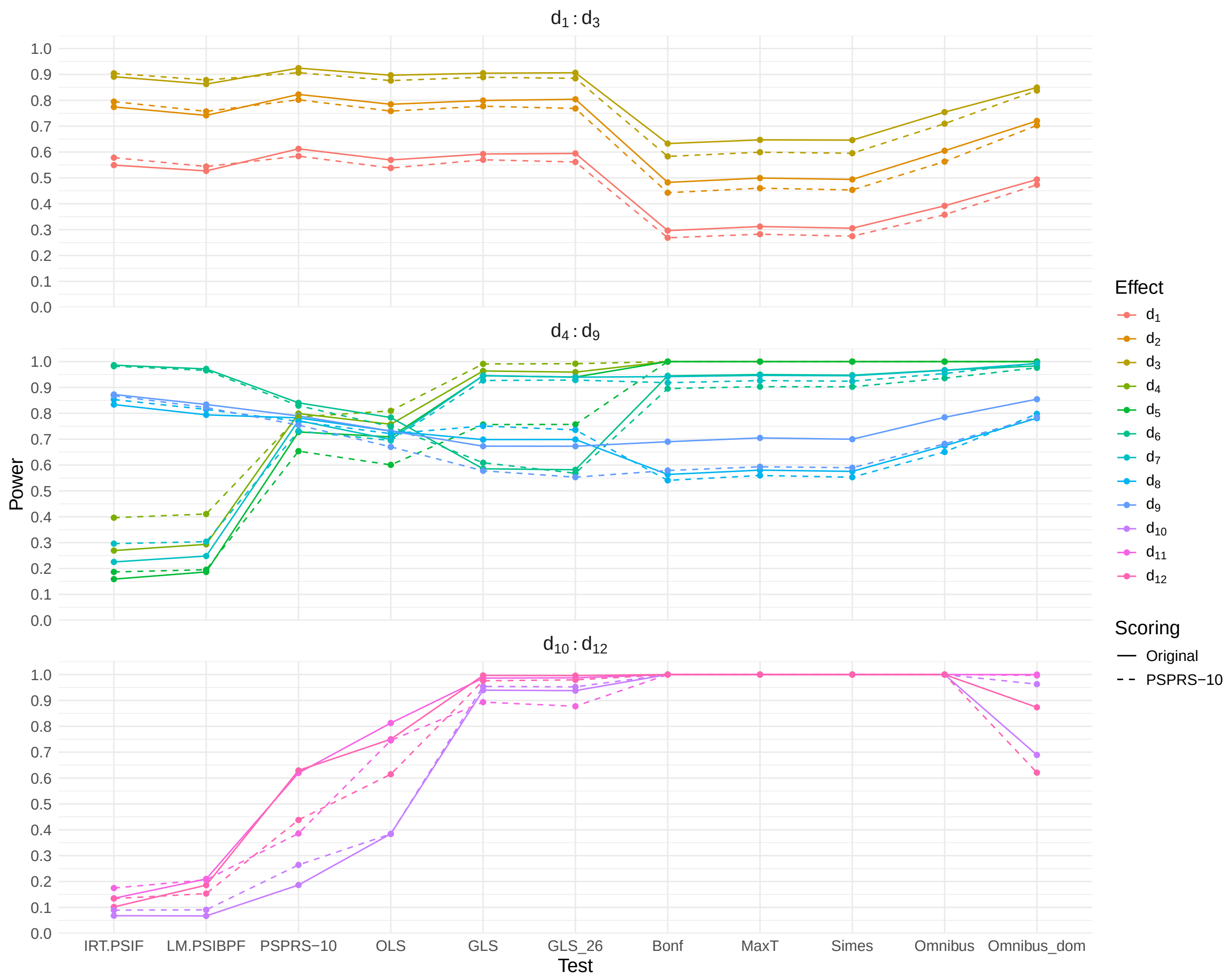

Supplementary Figure 2: Power of the considered testing procedures for the simulations based on the Bootstrap method.

non-centrality parameters are the same as in the original scenario where $n = 70$.

In addition, for the sample size of $n = 70$, we considered further alternative hypotheses by modifying the effect size scenarios in Table 4 of the main manuscript, assuming a minimum effect size in all items. Thus, we modified the scenarios in Table 4, replacing all 0s with 0.1. To avoid scenarios with very large power, other effect sizes were reduced where appropriate. See Supplementary Table 7 for the resulting scenarios.

Modified correlation matrices and effect size scenarios Furthermore, for the sample size of $n = 70$, we performed simulations based on the discretised multivariate normal distribution under a range of correlation scenarios between the different item scores and the item scores at baseline and follow-up. The 20×20 correlation matrix $\mathbf{R}$ of the 20-dimensional vector of the 10 item scores

Supplementary Table 6: Correlation matrix of item scores. Items of Table 1 of the main manuscript are respectively shown with numbers 1-10 and B abbreviates baseline.

	1-W52	2-W52	3-W52	4-W52	5-W52	6-W52	7-W52	8-W52	9-W52	10-W52	1-B	2-B	3-B	4-B	5-B	6-B	7-B	8-B	9-B	10-B
1-W52	1.00	0.40	0.20	0.38	0.45	0.19	0.28	0.36	0.29	0.32	0.58	0.30	0.21	0.31	0.30	0.19	0.28	0.25	0.19	0.36
2-W52	0.40	1.00	0.35	0.46	0.42	0.38	0.53	0.53	0.43	0.53	0.21	0.46	0.26	0.31	0.28	0.34	0.38	0.40	0.34	0.35
3-W52	0.20	0.35	1.00	0.22	0.15	0.28	0.28	0.39	0.38	0.32	0.11	0.24	0.47	0.13	0.07	0.21	0.22	0.24	0.19	0.21
4-W52	0.38	0.46	0.22	1.00	0.42	0.30	0.41	0.40	0.33	0.44	0.34	0.33	0.23	0.57	0.31	0.25	0.32	0.36	0.32	0.33
5-W52	0.45	0.42	0.15	0.42	1.00	0.28	0.35	0.36	0.29	0.32	0.35	0.27	0.16	0.33	0.38	0.25	0.23	0.21	0.19	0.29
6-W52	0.19	0.38	0.28	0.30	0.28	1.00	0.41	0.40	0.44	0.38	0.21	0.37	0.23	0.15	0.05	0.63	0.30	0.24	0.34	0.33
7-W52	0.28	0.53	0.28	0.41	0.35	0.41	1.00	0.77	0.68	0.77	0.25	0.34	0.25	0.33	0.22	0.29	0.57	0.52	0.51	0.53
8-W52	0.36	0.53	0.39	0.40	0.36	0.40	0.77	1.00	0.73	0.79	0.30	0.32	0.27	0.26	0.15	0.28	0.56	0.65	0.57	0.57
9-W52	0.29	0.43	0.38	0.33	0.29	0.44	0.68	0.73	1.00	0.66	0.26	0.27	0.28	0.21	0.09	0.30	0.49	0.50	0.61	0.46
10-W52	0.32	0.53	0.32	0.44	0.32	0.38	0.77	0.79	0.66	1.00	0.25	0.33	0.22	0.24	0.22	0.29	0.53	0.56	0.53	0.57
1-B	0.58	0.21	0.11	0.34	0.35	0.21	0.25	0.30	0.26	0.25	1.00	0.23	0.16	0.32	0.31	0.23	0.30	0.26	0.22	0.32
2-B	0.30	0.46	0.24	0.33	0.27	0.37	0.34	0.32	0.27	0.33	0.23	1.00	0.27	0.29	0.17	0.28	0.42	0.33	0.30	0.42
3-B	0.21	0.26	0.47	0.23	0.16	0.23	0.25	0.27	0.28	0.22	0.16	0.27	1.00	0.21	0.08	0.22	0.30	0.32	0.28	0.25
4-B	0.31	0.31	0.13	0.57	0.33	0.15	0.33	0.26	0.21	0.24	0.32	0.29	0.21	1.00	0.31	0.08	0.32	0.31	0.25	0.24
5-B	0.30	0.28	0.07	0.31	0.38	0.05	0.22	0.15	0.09	0.22	0.31	0.17	0.08	0.31	1.00	0.03	0.15	0.20	0.15	0.22
6-B	0.19	0.34	0.21	0.25	0.25	0.63	0.29	0.28	0.30	0.29	0.23	0.28	0.22	0.08	0.03	1.00	0.25	0.25	0.28	0.27
7-B	0.28	0.38	0.22	0.32	0.23	0.30	0.57	0.56	0.49	0.53	0.30	0.42	0.30	0.32	0.15	0.25	1.00	0.63	0.61	0.63
8-B	0.25	0.40	0.24	0.36	0.21	0.24	0.52	0.65	0.50	0.56	0.26	0.33	0.32	0.31	0.20	0.25	0.63	1.00	0.64	0.62
9-B	0.19	0.34	0.19	0.32	0.19	0.34	0.51	0.57	0.61	0.53	0.22	0.30	0.28	0.25	0.15	0.28	0.61	0.64	1.00	0.60
10-B	0.36	0.35	0.21	0.33	0.29	0.33	0.53	0.57	0.46	0.57	0.32	0.42	0.25	0.24	0.22	0.27	0.63	0.62	0.60	1.00

Supplementary Table 7: Additional effect size scenarios with small effects in all items and larger effects in a few items or domains. The rows correspond to vectors $\tilde{\boldsymbol{d}}$ of effect sizes for the 10 items in the FDA recommended modification of the PSPRS scale. The first three scenarios are the same as the initial d_1-d_3 of Table 4 of the main manuscript.

		Dysp.FS	Use.KF	Fall	Dysa	Dysp.	Neck.Ri	Ari.FC	Gait	Pos.St	Sit
Equal effect size	$\boldsymbol{d}_1$	0.20	0.20	0.20	0.20	0.20	0.20	0.20	0.20	0.20	0.20
Equal effect size	$\boldsymbol{d}_2$	0.25	0.25	0.25	0.25	0.25	0.25	0.25	0.25	0.25	0.25
Equal effect size	$\boldsymbol{d}_3$	0.30	0.30	0.30	0.30	0.30	0.30	0.30	0.30	0.30	0.30
History domain	$\tilde{\boldsymbol{d}}_4$	0.60	0.60	0.60	0.10	0.10	0.10	0.10	0.10	0.10	0.10
Bulbar exam	$\tilde{\boldsymbol{d}}_5$	0.10	0.10	0.10	0.85	0.85	0.10	0.10	0.10	0.10	0.10
Gait/midline exam	$\tilde{\boldsymbol{d}}_6$	0.10	0.10	0.10	0.10	0.10	0.40	0.40	0.40	0.40	0.40
History domain & Bulbar exam	$\tilde{\boldsymbol{d}}_7$	0.40	0.40	0.40	0.40	0.40	0.10	0.10	0.10	0.10	0.10
History domain & Gait/midline exam	$\tilde{\boldsymbol{d}}_8$	0.30	0.30	0.30	0.10	0.10	0.30	0.30	0.30	0.30	0.30
Bulbar & Gait/midline exam	$\tilde{\boldsymbol{d}}_9$	0.10	0.10	0.10	0.30	0.30	0.30	0.30	0.30	0.30	0.30
Dysphagia for solids (in History domain)	$\tilde{\boldsymbol{d}}_{10}$	1.60	0.10	0.10	0.10	0.10	0.10	0.10	0.10	0.10	0.10
Dysarthria (in Bulbar exam)	$\tilde{\boldsymbol{d}}_{11}$	0.10	0.10	0.10	1.60	0.10	0.10	0.10	0.10	0.10	0.10
Neck rigidity (in Gait/midline exam)	$\tilde{\boldsymbol{d}}_{12}$	0.10	0.10	0.10	0.10	0.10	1.60	0.10	0.10	0.10	0.10

before discretisation, measured at baseline and follow-up, can be partitioned into 4 sub-matrices:

$$\mathbf{R} = \left[\begin{array}{c|c} \mathbf{R}_1 & \mathbf{R}_2 \\ \hline \mathbf{R}_3 & \mathbf{R}_4 \end{array}\right]$$

where $\mathbf{R}_1$ is the sub-matrix of correlations between item scores at week 52 and $\mathbf{R}_4$ is the sub-matrix of correlations at baseline. $\mathbf{R}_2$ is the sub-matrix of correlations between item scores at week 52 and baseline, such that $\mathbf{R}_3 = \mathbf{R}_2^T$. The items are further grouped into domains as outlined in Table 1 of the main manuscript. We assumed equal low, moderate, and high correlations between items within each domain and a possibly lower equal correlation between items in different domains, such that the sub-matrices $\mathbf{R}_1$ and $\mathbf{R}_4$ have a block structure. We assumed equal between-item correlations at baseline and follow-up, i.e., $\mathbf{R}_1 = \mathbf{R}_4$.

Thus, the correlation matrices are parameterised by three constants: the correlation between item scores belonging to the same domain at a specific visit, denoted by r_{wd} (within-domain correlation); the correlation between item scores at a specific visit belonging to different domains, denoted by r_{bd} (between-domain correlation); and the correlation between item scores of a specific item at baseline and at follow-up, denoted by r_{bf}. The correlation between item i at baseline and item j at follow-up is then given by $r_{bf} \cdot r_{wd}$ or $r_{bf} \cdot r_{bd}$, depending on whether the items belong to the same or different domains. This defines the sub-matrices $\mathbf{R}_2$ and $\mathbf{R}_3$.

In total, 8 correlation scenarios have been considered, which are summarised in Supplementary Table 8. The variance of all item scores (before discretisation) at week 52 and baseline was assumed to be one. The mean item scores before discretisation in the control group at baseline and follow-up were assumed to be 1.6 and 2.2, respectively. These values are the mean item scores in the placebo group observed in the ABBV-8E12 trial data at week 52 and at baseline (see Supplementary Table 4). Other aspects of the simulations, including the effect size scenarios $\mathbf{d}$ (Table 4 of the main manuscript) and sample size $n = 70$, are as described in the simulation study reported in the main manuscript (see Section 4.1).

Furthermore, in selected scenarios where there is an effect in a single item or selected domains only, the power of some of the considered methods is equal to 1, and therefore no meaningful comparison between these methods is possible (scenarios d_4, d_5, and $d_{10} - d_{12}$ of Table 4 in the main manuscript). Thererfore, we considered an additional alternative with smaller effect sizes in these scenarios for the case with the correlations $r_{wd} = 0.1$, $r_{bd} = 0.1$ and $r_{bf} = 0.5$. Specifically, in scenarios d_4 and d_5, the non-zero effect sizes have been reduced to 0.45 and 0.5, respectively, and in $d_{10} - d_{12}$ to 0.6.

2.2 Results of the additional simulations

We assessed the type I error rate and power of each of the above simulation settings with 10^4 simulation runs per scenario.

Additional sample sizes and effect size scenarios Supplementary Figure 3 presents the type I error rate estimates for the additional sample sizes. For the GLS test we observed an inflated type I error rate for the case of $n = 35$. The MaxT test exhausts the significance level in contrast to the Bonferroni and Simes tests which are conservative.

Supplementary Table 8: Assumed correlations between items. The correlation between item scores belonging to the same domain at a specific visit is denoted by r_{wd} (within-domain correlation); the correlation between item scores at a specific visit belonging to different domains is denoted by r_{bd} (between-domain correlation); and the correlation between item scores of a specific item at baseline and at follow-up is denoted by r_{bf}.

r_{wd}	r_{bd}	r_{bf}
0	0	$0, 0.5$
0.1	0.1	$0, 0.5$
0.5	0.1	$0, 0.5$
0.8	0.5	$0, 0.5$

Supplementary Figures 6-11 and Supplementary Tables 9-11 summarise the power of the different methods for the additional simulations with additional sample sizes and effect size scenarios, both for the original scoring of the items as well as the PSPRS-10 scoring.

The simulations with a sample size of $n = 140$ and correspondingly rescaled effect sizes gave very similar results to the case of $n = 70$ under the alternatives listed in Table 4. For the case of $n = 35$, where the effect sizes were not rescaled, as expected, the power drops accordingly. In addition, the type I error rate for the GLS test is inflated.

In scenarios where there is a small effect in most items and a strong effect in selected items, especially the IRT-based test and the PSPRS-10-based tests (with both scoring methods) show improved power compared to the scenarios considered in the main manuscript, which have no effect in most items but a strong effect in the selected items.

Modified correlation matrices and effect size scenarios Supplementary Figures 4 and 5 presents the type I error rate estimates for the modified correlation scenarios. The GLS test is observed to have an inflated type I error rate estimate for the cases with lower within-domain and between-domain correlations. For the cases where r_{wd} and r_{bd} are higher the Bonferroni test and Simes test are extremely conservative. In contrast the MaxT test which accounts for the correlations exhausts the significance level.

Supplementary Figures 12-27 and Supplementary Tables 12-20 summarise the power of the different methods for the additional simulation scenarios, both for the original scoring of the items as well as the PSPRS-10 scoring, assuming correlations of the item scores as defined in Supplementary Table 8. In general, we observe a higher power if the correlation between items at baseline and follow-up is higher (if $r_{bf} = 0.5$ compared to $r_{bf} = 0$). As the correlation between items within a domain or between domains (the first two columns in Supplementary Table 8) increases, the resulting power decreases. However, the rank of the methods with respect to power does not sensitively depend on the correlations.

Finally, in the scenarios where there is an effect in a single item or selected domains, but the effect has been chosen smaller than in Table 4 in the main manuscript, we observe that the Bonferroni, MaxT, and Simes tests have very similar power values and are more powerful than all tests but the two Omnibus procedures. The Omnibus procedure has even larger power in this setting. The Omnibus-dom procedure has even larger power than the Omnibus procedure only if

the effect is homogeneous within domains. However, if there is an effect in a single item only, the power drops substantially (see Supplementary Table 16) and is even lower than the power of the Bonferroni, MaxT and Simes test.

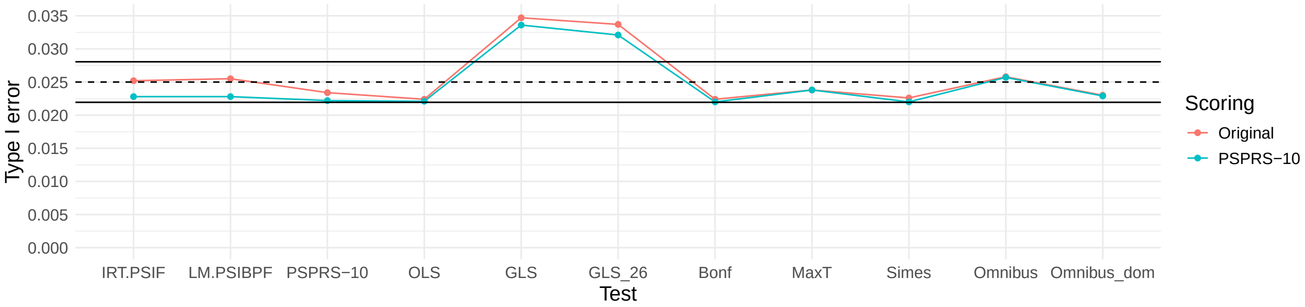

(a) n=35

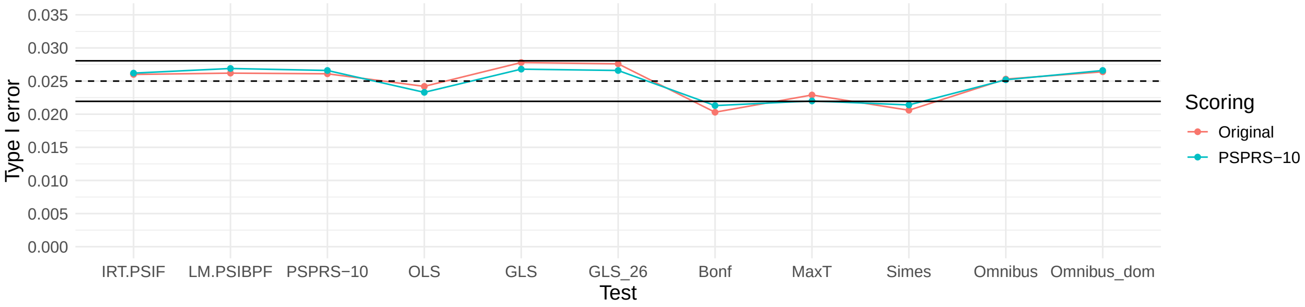

(b) n=70

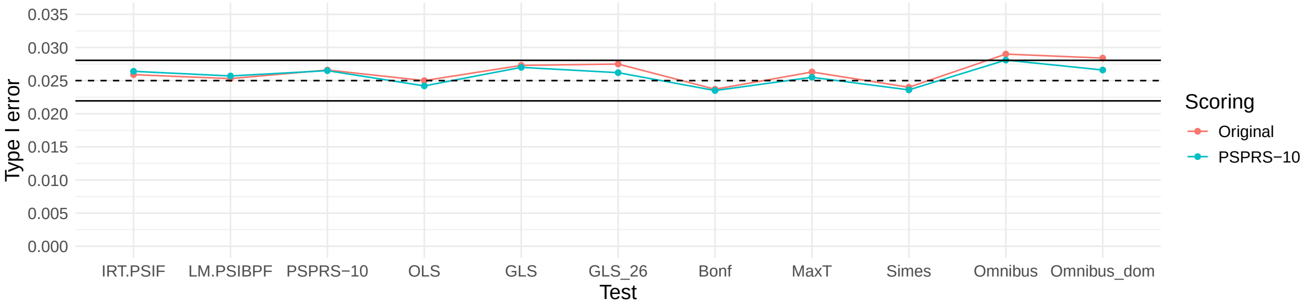

(c) n=140

Supplementary Figure 3: Type I error rates of the hypothesis tests for the simulations with additional samples sizes. The dashed lines represent the nominal significance level of 0.025 (one-sided). The black solid lines represent the 95% prediction limits (0.02194, 0.02806) for the estimated type I error rate from 10,000 simulation runs when the actual type I error is 0.025.

3 Multivariate tests

This section aims at providing technical details about the OLS and GLS tests. Let n_1 and n_2 denote, respectively, the number of subjects for the control and test groups. Further, $m \geq 2$

Supplementary Table 9: Power of the considered testing procedures for the simulation based on discretised multivariate normal scores, $n = 35$ with the original effect size scenarios $\boldsymbol{d}$ as defined in Table 4 in the main manuscript.

Original scoring

Scenario	IRT.PSIF	LM.PSIBPF	PSPRS-10	OLS	GLS	GLS-26	Bonf	MaxT	Simes	Omnibus	Omnibus-dom
d_1	0.282	0.288	0.380	0.336	0.405	0.400	0.195	0.208	0.199	0.250	0.304
d_2	0.409	0.418	0.544	0.488	0.557	0.554	0.287	0.303	0.293	0.370	0.441
d_3	0.550	0.556	0.697	0.645	0.702	0.699	0.410	0.426	0.418	0.506	0.596
d_4	0.139	0.159	0.495	0.476	0.752	0.747	0.976	0.978	0.977	0.984	0.996
d_5	0.109	0.123	0.512	0.475	0.704	0.696	1.000	1.000	1.000	1.000	1.000
d_6	0.741	0.735	0.553	0.499	0.346	0.349	0.657	0.675	0.666	0.728	0.779
d_7	0.142	0.154	0.532	0.481	0.743	0.738	0.695	0.712	0.704	0.763	0.879
d_8	0.475	0.480	0.508	0.464	0.483	0.484	0.352	0.368	0.358	0.434	0.502
d_9	0.534	0.532	0.537	0.471	0.435	0.437	0.425	0.441	0.432	0.508	0.593
d_{10}	0.053	0.051	0.116	0.230	0.707	0.701	1.000	1.000	1.000	1.000	0.363
d_{11}	0.087	0.128	0.390	0.536	0.826	0.825	1.000	1.000	1.000	1.000	0.999
d_{12}	0.069	0.117	0.404	0.479	0.887	0.883	1.000	1.000	1.000	1.000	0.608
min	0.053	0.051	0.116	0.230	0.346	0.349	0.195	0.208	0.199	0.250	0.304

PSPRS-10 scoring

Scenario	IRT.PSIF	LM.PSIBPF	PSPRS-10	OLS	GLS	GLS-26	Bonf	MaxT	Simes	Omnibus	Omnibus-dom
d_1	0.272	0.274	0.340	0.294	0.358	0.352	0.168	0.177	0.172	0.215	0.268
d_2	0.390	0.397	0.490	0.436	0.507	0.494	0.250	0.262	0.255	0.320	0.400
d_3	0.531	0.534	0.645	0.586	0.648	0.638	0.360	0.373	0.367	0.449	0.544
d_4	0.161	0.178	0.413	0.439	0.699	0.704	0.963	0.967	0.964	0.975	0.996
d_5	0.109	0.117	0.440	0.404	0.615	0.615	0.998	0.998	0.998	0.998	1.000
d_6	0.694	0.690	0.518	0.450	0.322	0.302	0.570	0.585	0.577	0.641	0.706
d_7	0.151	0.155	0.452	0.429	0.679	0.683	0.621	0.636	0.627	0.693	0.832
d_8	0.452	0.453	0.454	0.418	0.443	0.431	0.309	0.322	0.314	0.380	0.461
d_9	0.498	0.499	0.496	0.418	0.383	0.367	0.349	0.362	0.354	0.422	0.505
d_{10}	0.060	0.060	0.156	0.234	0.682	0.681	1.000	1.000	1.000	1.000	0.666
d_{11}	0.111	0.130	0.232	0.504	0.777	0.775	1.000	1.000	1.000	1.000	0.912
d_{12}	0.084	0.096	0.258	0.379	0.789	0.790	1.000	1.000	1.000	1.000	0.335
min	0.060	0.060	0.156	0.234	0.322	0.302	0.168	0.177	0.172	0.215	0.268

Supplementary Table 10: Power of the considered testing procedures for the simulation based on discretised multivariate normal scores, $n = 70$ with additional effect size scenarios $\tilde{\boldsymbol{d}}$ defined in Supplementary Table 7. The first three scenarios (d_1-d_3) which result in the same power as those in Supplementary Table 1 are not presented.

Original scoring

Scenario	IRT.PSIF	LM.PSIBPF	PSPRS-10	OLS	GLS	GLS-26	Bonf	MaxT	Simes	Omnibus	Omnibus-dom
$\tilde{d}_4$	0.422	0.450	0.817	0.786	0.918	0.917	0.985	0.986	0.986	0.990	0.998
$\tilde{d}_5$	0.370	0.396	0.839	0.781	0.895	0.893	1.000	1.000	1.000	1.000	1.000
$\tilde{d}_6$	0.896	0.893	0.842	0.797	0.679	0.687	0.800	0.811	0.808	0.861	0.899
$\tilde{d}_7$	0.413	0.436	0.834	0.786	0.916	0.914	0.839	0.847	0.844	0.890	0.957
$\tilde{d}_8$	0.803	0.806	0.868	0.819	0.823	0.826	0.662	0.676	0.672	0.758	0.826
$\tilde{d}_9$	0.756	0.758	0.817	0.761	0.732	0.736	0.627	0.643	0.636	0.720	0.801
$\tilde{d}_{10}$	0.288	0.280	0.554	0.731	0.971	0.971	1.000	1.000	1.000	1.000	0.850
$\tilde{d}_{11}$	0.394	0.457	0.828	0.850	0.936	0.937	1.000	1.000	1.000	1.000	1.000
$\tilde{d}_{12}$	0.324	0.416	0.821	0.809	0.969	0.969	1.000	1.000	1.000	1.000	0.879

PSPRS-10 scoring

Scenario	IRT.PSIF	LM.PSIBPF	PSPRS-10	OLS	GLS	GLS-26	Bonf	MaxT	Simes	Omnibus	Omnibus-dom
$\tilde{d}_4$	0.443	0.459	0.746	0.735	0.880	0.881	0.976	0.977	0.976	0.983	0.997
$\tilde{d}_5$	0.368	0.377	0.772	0.707	0.829	0.830	0.997	0.997	0.997	0.998	0.999
$\tilde{d}_6$	0.868	0.870	0.809	0.740	0.629	0.607	0.717	0.729	0.725	0.786	0.847
$\tilde{d}_7$	0.421	0.433	0.768	0.733	0.871	0.873	0.776	0.786	0.782	0.842	0.931
$\tilde{d}_8$	0.779	0.785	0.819	0.768	0.771	0.763	0.603	0.618	0.612	0.697	0.792
$\tilde{d}_9$	0.730	0.734	0.776	0.701	0.675	0.660	0.532	0.547	0.542	0.629	0.716
$\tilde{d}_{10}$	0.304	0.301	0.608	0.711	0.961	0.961	1.000	1.000	1.000	1.000	0.974
$\tilde{d}_{11}$	0.387	0.411	0.622	0.753	0.874	0.871	1.000	1.000	1.000	1.000	0.961
$\tilde{d}_{12}$	0.328	0.347	0.653	0.703	0.911	0.913	1.000	1.000	1.000	1.000	0.656

Supplementary Table 11: Power of the considered testing procedures for the simulation based on discretised multivariate normal scores, $n = 140$ with the rescaled effect size scenarios $c\boldsymbol{d}$, where $\boldsymbol{d}$ is defined in Table 4 in the main manuscript and $c = \sqrt{70}/\sqrt{140}$.

Original scoring

Scenario	IRT.PSIF	LM.PSIBPF	PSPRS-10	OLS	GLS	GLS-26	Bonf	MaxT	Simes	Omnibus	Omnibus-dom
cd_1	0.508	0.522	0.659	0.594	0.628	0.631	0.368	0.386	0.377	0.460	0.552
cd_2	0.694	0.702	0.836	0.786	0.818	0.821	0.549	0.566	0.559	0.658	0.747
cd_3	0.851	0.855	0.944	0.917	0.933	0.935	0.741	0.754	0.752	0.836	0.896
cd_4	0.269	0.300	0.830	0.809	0.963	0.961	1.000	1.000	1.000	1.000	1.000
cd_5	0.204	0.227	0.842	0.784	0.932	0.931	1.000	1.000	1.000	1.000	1.000
cd_6	0.961	0.958	0.849	0.797	0.542	0.556	0.956	0.959	0.959	0.972	0.985
cd_7	0.242	0.268	0.833	0.786	0.953	0.952	0.972	0.973	0.973	0.985	0.997
cd_8	0.767	0.769	0.804	0.753	0.735	0.740	0.660	0.676	0.672	0.755	0.818
cd_9	0.837	0.833	0.837	0.777	0.684	0.691	0.771	0.784	0.780	0.842	0.901
cd_{10}	0.103	0.094	0.335	0.660	0.994	0.993	1.000	1.000	1.000	1.000	0.948
cd_{11}	0.197	0.282	0.811	0.879	0.977	0.976	1.000	1.000	1.000	1.000	1.000
cd_{12}	0.138	0.241	0.796	0.821	0.995	0.995	1.000	1.000	1.000	1.000	0.974
min	0.103	0.094	0.335	0.594	0.542	0.556	0.368	0.386	0.377	0.460	0.552

PSPRS-10 scoring

Scenario	IRT.PSIF	LM.PSIBPF	PSPRS-10	OLS	GLS	GLS-26	Bonf	MaxT	Simes	Omnibus	Omnibus-dom
cd_1	0.488	0.496	0.605	0.539	0.574	0.568	0.321	0.337	0.327	0.407	0.501
cd_2	0.677	0.685	0.790	0.732	0.764	0.757	0.479	0.494	0.489	0.584	0.694
cd_3	0.830	0.838	0.915	0.877	0.897	0.894	0.668	0.682	0.679	0.772	0.856
cd_4	0.313	0.330	0.739	0.764	0.940	0.944	1.000	1.000	1.000	1.000	1.000
cd_5	0.206	0.215	0.766	0.707	0.872	0.878	1.000	1.000	1.000	1.000	1.000
cd_6	0.942	0.942	0.823	0.738	0.506	0.478	0.907	0.912	0.911	0.938	0.967
cd_7	0.265	0.280	0.749	0.733	0.918	0.924	0.948	0.951	0.951	0.970	0.992
cd_8	0.748	0.749	0.755	0.698	0.688	0.676	0.602	0.615	0.612	0.696	0.789
cd_9	0.802	0.799	0.799	0.712	0.623	0.606	0.670	0.685	0.680	0.755	0.835
cd_{10}	0.125	0.125	0.462	0.678	0.991	0.992	1.000	1.000	1.000	1.000	1.000
cd_{11}	0.213	0.247	0.496	0.767	0.928	0.928	1.000	1.000	1.000	1.000	1.000
cd_{12}	0.159	0.183	0.547	0.674	0.964	0.967	1.000	1.000	1.000	1.000	0.758
min	0.125	0.125	0.462	0.539	0.506	0.478	0.321	0.337	0.327	0.407	0.501

Supplementary Table 12: Power of the considered testing procedures for the simulation based on discretised multivariate normal scores with the correlations $r_{wd} = 0$, $r_{bd} = 0$ and $r_{bf} = 0$.

Original scoring

Scenario	IRT.PSIF	LM.PSIBPF	PSPRS-10	OLS	GLS	GLS-26	Bonf	MaxT	Simes	Omnibus	Omnibus-dom
d_1	0.806	0.833	0.944	0.942	0.944	0.920	0.372	0.374	0.377	0.465	0.797
d_2	0.939	0.953	0.993	0.994	0.993	0.988	0.561	0.565	0.573	0.693	0.948
d_3	0.987	0.992	0.999	0.999	1.000	0.998	0.744	0.747	0.758	0.873	0.993
d_4	0.474	0.532	0.995	0.994	0.985	0.993	1.000	1.000	1.000	1.000	1.000
d_5	0.409	0.356	0.992	0.994	0.978	0.986	1.000	1.000	1.000	1.000	1.000
d_6	1.000	1.000	0.993	0.993	0.988	0.954	0.967	0.968	0.971	0.988	1.000
d_7	0.438	0.470	0.994	0.994	0.990	0.994	0.964	0.965	0.967	0.987	1.000
d_8	0.967	0.977	0.988	0.988	0.988	0.971	0.660	0.663	0.672	0.779	0.977
d_9	0.988	0.991	0.991	0.991	0.990	0.972	0.789	0.791	0.800	0.883	0.994
d_{10}	0.273	0.269	0.950	0.991	0.952	0.968	1.000	1.000	1.000	1.000	1.000
d_{11}	0.372	0.380	0.953	0.992	0.958	0.970	1.000	1.000	1.000	1.000	1.000
d_{12}	0.238	0.316	0.951	0.991	0.954	0.968	1.000	1.000	1.000	1.000	0.998
min	0.238	0.269	0.944	0.942	0.944	0.920	0.372	0.374	0.377	0.465	0.797

PSPRS-10 scoring

Scenario	IRT.PSIF	LM.PSIBPF	PSPRS-10	OLS	GLS	GLS-26	Bonf	MaxT	Simes	Omnibus	Omnibus-dom
d_1	0.765	0.810	0.900	0.908	0.912	0.878	0.324	0.327	0.328	0.398	0.715
d_2	0.914	0.938	0.981	0.982	0.982	0.970	0.496	0.498	0.504	0.611	0.898
d_3	0.980	0.988	0.998	0.998	0.998	0.996	0.673	0.677	0.688	0.807	0.979
d_4	0.442	0.594	0.980	0.980	0.969	0.982	1.000	1.000	1.000	1.000	1.000
d_5	0.362	0.369	0.983	0.988	0.970	0.980	1.000	1.000	1.000	1.000	1.000
d_6	0.999	0.999	0.979	0.981	0.977	0.922	0.931	0.932	0.937	0.967	1.000
d_7	0.393	0.502	0.981	0.983	0.980	0.987	0.927	0.928	0.932	0.967	0.998
d_8	0.951	0.966	0.969	0.972	0.971	0.942	0.583	0.585	0.591	0.687	0.946
d_9	0.980	0.984	0.983	0.982	0.982	0.952	0.720	0.722	0.730	0.822	0.986
d_{10}	0.301	0.424	0.995	0.990	0.954	0.969	1.000	1.000	1.000	1.000	1.000
d_{11}	0.364	0.360	0.738	0.983	0.945	0.959	1.000	1.000	1.000	1.000	1.000
d_{12}	0.244	0.243	0.805	0.965	0.910	0.933	1.000	1.000	1.000	1.000	0.951
min	0.244	0.243	0.738	0.908	0.910	0.878	0.324	0.327	0.328	0.398	0.715

Supplementary Table 13: Power of the considered testing procedures for the simulation based on discretised multivariate normal scores with the correlations $r_{wd} = 0$, $r_{bd} = 0$ and $r_{bf} = 0.5$.

Original scoring

Scenario	IRT.PSIF	LM.PSIBPF	PSPRS-10	OLS	GLS	GLS-26	Bonf	MaxT	Simes	Omnibus	Omnibus-dom
d_1	0.879	0.905	0.977	0.976	0.976	0.962	0.466	0.469	0.475	0.584	0.887
d_2	0.974	0.982	0.999	0.998	0.998	0.997	0.680	0.683	0.693	0.816	0.984
d_3	0.996	0.998	1.000	1.000	1.000	1.000	0.854	0.856	0.868	0.947	0.999
d_4	0.553	0.630	0.999	0.999	0.994	0.998	1.000	1.000	1.000	1.000	1.000
d_5	0.484	0.431	0.998	0.998	0.989	0.993	1.000	1.000	1.000	1.000	1.000
d_6	1.000	1.000	0.999	0.999	0.997	0.981	0.992	0.992	0.994	0.998	1.000
d_7	0.517	0.560	0.999	0.999	0.998	0.999	0.992	0.992	0.993	0.998	1.000
d_8	0.988	0.993	0.997	0.997	0.997	0.991	0.785	0.787	0.797	0.886	0.995
d_9	0.997	0.998	0.998	0.998	0.996	0.990	0.888	0.890	0.899	0.952	0.999
d_{10}	0.328	0.332	0.984	0.997	0.970	0.983	1.000	1.000	1.000	1.000	1.000
d_{11}	0.445	0.455	0.983	0.998	0.972	0.981	1.000	1.000	1.000	1.000	1.000
d_{12}	0.287	0.389	0.983	0.997	0.967	0.977	1.000	1.000	1.000	1.000	1.000
min	0.287	0.332	0.977	0.976	0.967	0.962	0.466	0.469	0.475	0.584	0.887

PSPRS-10 scoring

Scenario	IRT.PSIF	LM.PSIBPF	PSPRS-10	OLS	GLS	GLS-26	Bonf	MaxT	Simes	Omnibus	Omnibus-dom
d_1	0.830	0.870	0.946	0.949	0.944	0.921	0.384	0.387	0.392	0.486	0.798
d_2	0.952	0.972	0.994	0.994	0.993	0.989	0.584	0.586	0.594	0.719	0.949
d_3	0.991	0.996	1.000	1.000	1.000	0.999	0.764	0.767	0.778	0.886	0.994
d_4	0.501	0.670	0.993	0.992	0.983	0.991	1.000	1.000	1.000	1.000	1.000
d_5	0.417	0.428	0.995	0.996	0.983	0.991	1.000	1.000	1.000	1.000	1.000
d_6	1.000	1.000	0.993	0.993	0.989	0.954	0.969	0.970	0.973	0.989	1.000
d_7	0.441	0.567	0.995	0.995	0.992	0.996	0.972	0.973	0.976	0.990	1.000
d_8	0.974	0.984	0.987	0.987	0.986	0.968	0.680	0.683	0.690	0.795	0.977
d_9	0.990	0.992	0.993	0.993	0.991	0.979	0.812	0.815	0.822	0.899	0.997
d_{10}	0.345	0.487	0.998	0.997	0.969	0.981	1.000	1.000	1.000	1.000	1.000
d_{11}	0.421	0.415	0.815	0.992	0.957	0.970	1.000	1.000	1.000	1.000	1.000
d_{12}	0.284	0.286	0.873	0.981	0.931	0.951	1.000	1.000	1.000	1.000	0.977
min	0.284	0.286	0.815	0.949	0.931	0.921	0.384	0.387	0.392	0.486	0.798

Supplementary Table 14: Power of the considered testing procedures for the simulation based on discretised multivariate normal scores with the correlations $r_{wd} = 0.1$, $r_{bd} = 0.1$ and $r_{bf} = 0$.

Original scoring

Scenario	IRT.PSIF	LM.PSIBPF	PSPRS-10	OLS	GLS	GLS-26	Bonf	MaxT	Simes	Omnibus	Omnibus-dom
d_1	0.634	0.653	0.745	0.745	0.760	0.736	0.346	0.351	0.351	0.432	0.628
d_2	0.824	0.839	0.909	0.909	0.911	0.899	0.526	0.532	0.535	0.640	0.835
d_3	0.931	0.941	0.974	0.975	0.975	0.969	0.688	0.694	0.701	0.809	0.943
d_4	0.337	0.382	0.914	0.917	0.896	0.935	1.000	1.000	1.000	1.000	1.000
d_5	0.285	0.249	0.893	0.910	0.876	0.914	1.000	1.000	1.000	1.000	1.000
d_6	0.991	0.993	0.912	0.911	0.900	0.809	0.949	0.950	0.953	0.974	0.999
d_7	0.307	0.329	0.911	0.910	0.901	0.939	0.949	0.951	0.953	0.973	0.998
d_8	0.876	0.893	0.876	0.876	0.880	0.845	0.618	0.624	0.629	0.726	0.900
d_9	0.934	0.935	0.900	0.900	0.900	0.851	0.743	0.748	0.754	0.832	0.961
d_{10}	0.192	0.196	0.770	0.907	0.878	0.908	1.000	1.000	1.000	1.000	1.000
d_{11}	0.266	0.266	0.780	0.910	0.879	0.913	1.000	1.000	1.000	1.000	1.000
d_{12}	0.170	0.222	0.769	0.907	0.875	0.908	1.000	1.000	1.000	1.000	0.983
min	0.170	0.196	0.745	0.745	0.760	0.736	0.346	0.351	0.351	0.432	0.628

PSPRS-10 scoring

Scenario	IRT.PSIF	LM.PSIBPF	PSPRS-10	OLS	GLS	GLS-26	Bonf	MaxT	Simes	Omnibus	Omnibus-dom
d_1	0.604	0.633	0.704	0.710	0.721	0.695	0.304	0.309	0.310	0.379	0.575
d_2	0.795	0.819	0.880	0.881	0.882	0.866	0.463	0.469	0.472	0.576	0.778
d_3	0.916	0.935	0.961	0.962	0.961	0.951	0.631	0.636	0.641	0.747	0.916
d_4	0.331	0.434	0.877	0.878	0.865	0.906	0.999	0.999	0.999	1.000	1.000
d_5	0.263	0.263	0.879	0.895	0.856	0.897	1.000	1.000	1.000	1.000	1.000
d_6	0.986	0.985	0.876	0.884	0.875	0.765	0.904	0.906	0.909	0.948	0.995
d_7	0.288	0.360	0.872	0.877	0.871	0.911	0.907	0.908	0.912	0.946	0.993
d_8	0.852	0.871	0.838	0.842	0.847	0.804	0.546	0.553	0.554	0.651	0.852
d_9	0.916	0.915	0.878	0.879	0.877	0.824	0.683	0.688	0.692	0.776	0.940
d_{10}	0.220	0.304	0.939	0.922	0.865	0.899	1.000	1.000	1.000	1.000	1.000
d_{11}	0.271	0.261	0.521	0.891	0.864	0.896	1.000	1.000	1.000	1.000	1.000
d_{12}	0.182	0.177	0.584	0.831	0.807	0.847	1.000	1.000	1.000	1.000	0.873
min	0.182	0.177	0.521	0.710	0.721	0.695	0.304	0.309	0.310	0.379	0.575

Supplementary Table 15: Power of the considered testing procedures for the simulation based on discretised multivariate normal scores with the correlations $r_{wd} = 0.1$, $r_{bd} = 0.1$ and $r_{bf} = 0.5$.

Original scoring

Scenario	IRT.PSIF	LM.PSIBPF	PSPRS-10	OLS	GLS	GLS-26	Bonf	MaxT	Simes	Omnibus	Omnibus-dom
d_1	0.739	0.763	0.846	0.847	0.851	0.831	0.430	0.435	0.437	0.538	0.745
d_2	0.898	0.918	0.962	0.962	0.965	0.955	0.633	0.638	0.647	0.760	0.918
d_3	0.975	0.981	0.993	0.994	0.994	0.990	0.803	0.808	0.816	0.900	0.979
d_4	0.404	0.464	0.964	0.967	0.946	0.969	1.000	1.000	1.000	1.000	1.000
d_5	0.344	0.305	0.956	0.964	0.929	0.955	1.000	1.000	1.000	1.000	1.000
d_6	0.998	0.999	0.962	0.962	0.950	0.884	0.984	0.984	0.986	0.994	1.000
d_7	0.373	0.405	0.964	0.964	0.954	0.976	0.984	0.985	0.986	0.994	1.000
d_8	0.942	0.955	0.942	0.944	0.944	0.920	0.739	0.744	0.750	0.841	0.960
d_9	0.971	0.975	0.956	0.957	0.955	0.922	0.855	0.858	0.864	0.919	0.989
d_{10}	0.234	0.236	0.867	0.952	0.921	0.944	1.000	1.000	1.000	1.000	1.000
d_{11}	0.321	0.327	0.869	0.950	0.915	0.940	1.000	1.000	1.000	1.000	1.000
d_{12}	0.208	0.271	0.865	0.952	0.912	0.939	1.000	1.000	1.000	1.000	0.996
min	0.208	0.236	0.846	0.847	0.851	0.831	0.430	0.435	0.437	0.538	0.745

PSPRS-10 scoring

Scenario	IRT.PSIF	LM.PSIBPF	PSPRS-10	OLS	GLS	GLS-26	Bonf	MaxT	Simes	Omnibus	Omnibus-dom
d_1	0.692	0.728	0.797	0.796	0.801	0.777	0.363	0.368	0.369	0.460	0.674
d_2	0.867	0.891	0.937	0.938	0.938	0.924	0.549	0.554	0.559	0.670	0.870
d_3	0.959	0.970	0.987	0.987	0.986	0.980	0.719	0.725	0.732	0.839	0.960
d_4	0.383	0.511	0.936	0.936	0.914	0.947	1.000	1.000	1.000	1.000	1.000
d_5	0.315	0.319	0.940	0.951	0.908	0.941	1.000	1.000	1.000	1.000	1.000
d_6	0.995	0.996	0.932	0.933	0.922	0.839	0.953	0.954	0.956	0.977	1.000
d_7	0.336	0.426	0.936	0.938	0.926	0.954	0.962	0.963	0.965	0.984	0.999
d_8	0.913	0.929	0.912	0.911	0.907	0.874	0.644	0.650	0.653	0.750	0.925
d_9	0.955	0.959	0.940	0.939	0.929	0.884	0.783	0.786	0.791	0.861	0.977
d_{10}	0.257	0.365	0.976	0.963	0.910	0.938	1.000	1.000	1.000	1.000	1.000
d_{11}	0.314	0.303	0.614	0.931	0.895	0.921	1.000	1.000	1.000	1.000	1.000
d_{12}	0.216	0.210	0.684	0.891	0.854	0.890	1.000	1.000	1.000	1.000	0.934
min	0.216	0.210	0.614	0.796	0.801	0.777	0.363	0.368	0.369	0.460	0.674

Supplementary Table 16: Power of the considered testing procedures for the simulation based on discretised multivariate normal scores with the correlations $r_{wd} = 0.1$, $r_{bd} = 0.1$ and $r_{bf} = 0.5$. Note that in scenarios d_4 and d_5 (of Table 4 of the main manuscript) the non-zero effect sizes have been respectively reduced to 0.45, 0.5 and in $d_{10} - d_{12}$ to 0.6. The rest of scenarios are not considered.

Original scoring

Scenario	IRT.PSIF	LM.PSIBPF	PSPRS-10	OLS	GLS	GLS-26	Bonf	MaxT	Simes	Omnibus	Omnibus-dom
d_4	0.146	0.170	0.522	0.521	0.539	0.596	0.859	0.861	0.864	0.894	0.984
d_5	0.085	0.084	0.314	0.312	0.340	0.384	0.843	0.846	0.847	0.865	0.964
d_{10}	0.041	0.054	0.139	0.142	0.165	0.179	0.827	0.830	0.827	0.832	0.351
d_{11}	0.068	0.064	0.143	0.145	0.171	0.189	0.827	0.829	0.827	0.830	0.564
d_{12}	0.053	0.059	0.142	0.141	0.170	0.184	0.834	0.836	0.834	0.838	0.182

PSPRS-10 scoring

Scenario	IRT.PSIF	LM.PSIBPF	PSPRS-10	OLS	GLS	GLS-26	Bonf	MaxT	Simes	Omnibus	Omnibus-dom
d_4	0.134	0.179	0.450	0.452	0.478	0.528	0.770	0.773	0.775	0.810	0.950
d_5	0.078	0.084	0.294	0.298	0.317	0.358	0.779	0.782	0.782	0.807	0.937
d_{10}	0.044	0.068	0.203	0.151	0.165	0.179	0.827	0.830	0.827	0.832	0.571
d_{11}	0.058	0.058	0.084	0.125	0.152	0.165	0.673	0.676	0.674	0.681	0.220
d_{12}	0.052	0.052	0.103	0.125	0.150	0.160	0.694	0.698	0.695	0.701	0.109

Supplementary Table 17: Power of the considered testing procedures for the simulation based on discretised multivariate normal scores with the correlations $r_{wd} = 0.5$, $r_{bd} = 0.1$ and $r_{bf} = 0$.

Original scoring

Scenario	IRT.PSIF	LM.PSIBPF	PSPRS-10	OLS	GLS	GLS-26	Bonf	MaxT	Simes	Omnibus	Omnibus-dom
d_1	0.414	0.432	0.556	0.556	0.586	0.581	0.302	0.318	0.309	0.384	0.423
d_2	0.592	0.620	0.752	0.750	0.774	0.771	0.457	0.476	0.468	0.567	0.618
d_3	0.743	0.771	0.881	0.882	0.896	0.892	0.615	0.633	0.628	0.727	0.779
d_4	0.184	0.244	0.759	0.768	0.854	0.863	0.999	0.999	0.999	1.000	1.000
d_5	0.155	0.160	0.725	0.755	0.959	0.961	1.000	1.000	1.000	1.000	1.000
d_6	0.927	0.921	0.749	0.747	0.552	0.521	0.849	0.858	0.855	0.895	0.907
d_7	0.172	0.218	0.748	0.746	0.915	0.919	0.907	0.914	0.911	0.942	0.974
d_8	0.666	0.692	0.710	0.711	0.639	0.627	0.526	0.544	0.537	0.631	0.655
d_9	0.752	0.751	0.737	0.739	0.726	0.712	0.650	0.668	0.661	0.744	0.789
d_{10}	0.117	0.133	0.572	0.756	0.864	0.869	1.000	1.000	1.000	1.000	0.996
d_{11}	0.149	0.174	0.585	0.761	0.940	0.944	1.000	1.000	1.000	1.000	1.000
d_{12}	0.108	0.148	0.587	0.756	0.726	0.789	1.000	1.000	1.000	1.000	0.779
min	0.108	0.133	0.556	0.556	0.552	0.521	0.302	0.318	0.309	0.384	0.423

PSPRS-10 scoring

Scenario	IRT.PSIF	LM.PSIBPF	PSPRS-10	OLS	GLS	GLS-26	Bonf	MaxT	Simes	Omnibus	Omnibus-dom
d_1	0.402	0.428	0.528	0.527	0.556	0.549	0.269	0.281	0.274	0.344	0.392
d_2	0.577	0.611	0.727	0.722	0.742	0.734	0.411	0.428	0.422	0.517	0.585
d_3	0.737	0.762	0.865	0.861	0.872	0.867	0.563	0.580	0.577	0.684	0.748
d_4	0.204	0.284	0.714	0.717	0.828	0.837	0.997	0.997	0.997	0.998	1.000
d_5	0.160	0.175	0.722	0.742	0.935	0.943	1.000	1.000	1.000	1.000	1.000
d_6	0.907	0.896	0.721	0.728	0.534	0.495	0.802	0.809	0.807	0.857	0.886
d_7	0.178	0.242	0.711	0.716	0.884	0.894	0.867	0.875	0.874	0.914	0.962
d_8	0.648	0.675	0.676	0.675	0.610	0.594	0.471	0.484	0.480	0.572	0.619
d_9	0.735	0.730	0.724	0.722	0.688	0.670	0.602	0.617	0.613	0.697	0.762
d_{10}	0.144	0.207	0.814	0.791	0.821	0.832	1.000	1.000	1.000	1.000	1.000
d_{11}	0.168	0.175	0.377	0.745	0.921	0.929	1.000	1.000	1.000	1.000	0.998
d_{12}	0.123	0.124	0.424	0.663	0.619	0.689	1.000	1.000	1.000	1.000	0.557
min	0.123	0.124	0.377	0.527	0.534	0.495	0.269	0.281	0.274	0.344	0.392

Supplementary Table 18: Power of the considered testing procedures for the simulation based on discretised multivariate normal scores with the correlations $r_{wd} = 0.5$, $r_{bd} = 0.1$ and $r_{bf} = 0.5$.

Original scoring

Scenario	IRT.PSIF	LM.PSIBPF	PSPRS-10	OLS	GLS	GLS-26	Bonf	MaxT	Simes	Omnibus	Omnibus-dom
d_1	0.509	0.536	0.666	0.669	0.700	0.690	0.377	0.393	0.385	0.476	0.525
d_2	0.701	0.728	0.852	0.853	0.872	0.868	0.567	0.586	0.580	0.686	0.738
d_3	0.841	0.865	0.946	0.947	0.956	0.955	0.730	0.746	0.744	0.835	0.878
d_4	0.224	0.301	0.863	0.866	0.921	0.927	1.000	1.000	1.000	1.000	1.000
d_5	0.196	0.203	0.829	0.854	0.981	0.983	1.000	1.000	1.000	1.000	1.000
d_6	0.973	0.970	0.851	0.851	0.662	0.625	0.933	0.939	0.937	0.961	0.970
d_7	0.212	0.267	0.857	0.856	0.964	0.967	0.969	0.972	0.972	0.984	0.995
d_8	0.782	0.803	0.822	0.819	0.756	0.740	0.645	0.660	0.656	0.749	0.781
d_9	0.852	0.851	0.841	0.840	0.828	0.812	0.769	0.781	0.779	0.848	0.889
d_{10}	0.136	0.157	0.691	0.838	0.908	0.914	1.000	1.000	1.000	1.000	1.000
d_{11}	0.180	0.215	0.696	0.840	0.965	0.968	1.000	1.000	1.000	1.000	1.000
d_{12}	0.127	0.180	0.701	0.837	0.794	0.846	1.000	1.000	1.000	1.000	0.887
min	0.127	0.157	0.666	0.669	0.662	0.625	0.377	0.393	0.385	0.476	0.525

PSPRS-10 scoring

Scenario	IRT.PSIF	LM.PSIBPF	PSPRS-10	OLS	GLS	GLS-26	Bonf	MaxT	Simes	Omnibus	Omnibus-dom
d_1	0.486	0.516	0.631	0.630	0.650	0.643	0.334	0.345	0.340	0.421	0.485
d_2	0.676	0.710	0.822	0.818	0.831	0.823	0.500	0.513	0.510	0.617	0.689
d_3	0.821	0.849	0.929	0.923	0.929	0.926	0.663	0.677	0.675	0.776	0.849
d_4	0.245	0.351	0.816	0.812	0.892	0.903	0.999	1.000	1.000	1.000	1.000
d_5	0.198	0.215	0.817	0.835	0.963	0.969	1.000	1.000	1.000	1.000	1.000
d_6	0.957	0.950	0.816	0.815	0.628	0.579	0.883	0.889	0.889	0.922	0.951
d_7	0.220	0.290	0.813	0.815	0.941	0.946	0.940	0.944	0.944	0.967	0.991
d_8	0.749	0.775	0.779	0.773	0.708	0.691	0.569	0.581	0.579	0.667	0.736
d_9	0.829	0.824	0.826	0.816	0.783	0.761	0.710	0.721	0.719	0.797	0.860
d_{10}	0.166	0.247	0.895	0.876	0.872	0.881	1.000	1.000	1.000	1.000	1.000
d_{11}	0.198	0.210	0.456	0.816	0.945	0.952	1.000	1.000	1.000	1.000	1.000
d_{12}	0.144	0.148	0.524	0.744	0.685	0.753	1.000	1.000	1.000	1.000	0.679
min	0.144	0.148	0.456	0.630	0.628	0.579	0.334	0.345	0.340	0.421	0.485

Supplementary Table 19: Power of the considered testing procedures for the simulation based on discretised multivariate normal scores with the correlations $r_{wd} = 0.8$, $r_{bd} = 0.5$ and $r_{bf} = 0$.

Original scoring

Scenario	IRT.PSIF	LM.PSIBPF	PSPRS-10	OLS	GLS	GLS-26	Bonf	MaxT	Simes	Omnibus	Omnibus-dom
d_1	0.270	0.271	0.305	0.304	0.320	0.318	0.208	0.255	0.215	0.292	0.289
d_2	0.395	0.397	0.450	0.447	0.466	0.464	0.323	0.380	0.335	0.427	0.427
d_3	0.529	0.530	0.592	0.588	0.614	0.612	0.439	0.502	0.453	0.565	0.565
d_4	0.125	0.159	0.456	0.463	0.575	0.581	0.993	0.996	0.993	0.996	0.997
d_5	0.111	0.114	0.422	0.450	0.803	0.806	1.000	1.000	1.000	1.000	1.000
d_6	0.751	0.700	0.450	0.451	0.284	0.275	0.738	0.783	0.747	0.809	0.774
d_7	0.122	0.147	0.450	0.451	0.657	0.663	0.801	0.843	0.808	0.857	0.886
d_8	0.459	0.449	0.414	0.412	0.340	0.337	0.379	0.437	0.391	0.485	0.457
d_9	0.532	0.501	0.434	0.432	0.415	0.410	0.496	0.559	0.510	0.602	0.598
d_{10}	0.080	0.095	0.324	0.461	0.909	0.910	1.000	1.000	1.000	1.000	0.982
d_{11}	0.100	0.119	0.330	0.474	0.945	0.946	1.000	1.000	1.000	1.000	1.000
d_{12}	0.078	0.104	0.324	0.462	0.865	0.882	1.000	1.000	1.000	1.000	0.610
min	0.078	0.095	0.305	0.304	0.284	0.275	0.208	0.255	0.215	0.292	0.289

PSPRS-10 scoring

Scenario	IRT.PSIF	LM.PSIBPF	PSPRS-10	OLS	GLS	GLS-26	Bonf	MaxT	Simes	Omnibus	Omnibus-dom
d_1	0.268	0.275	0.298	0.298	0.300	0.296	0.204	0.236	0.210	0.276	0.279
d_2	0.389	0.400	0.439	0.438	0.435	0.433	0.310	0.350	0.320	0.407	0.412
d_3	0.525	0.536	0.580	0.577	0.570	0.567	0.423	0.467	0.434	0.534	0.546
d_4	0.157	0.194	0.425	0.425	0.545	0.562	0.990	0.993	0.991	0.993	0.996
d_5	0.123	0.124	0.433	0.452	0.705	0.722	1.000	1.000	1.000	1.000	1.000
d_6	0.712	0.676	0.438	0.441	0.274	0.251	0.697	0.730	0.705	0.765	0.741
d_7	0.141	0.166	0.430	0.432	0.608	0.629	0.775	0.809	0.782	0.832	0.877
d_8	0.444	0.455	0.403	0.402	0.340	0.329	0.362	0.405	0.372	0.457	0.440
d_9	0.514	0.494	0.435	0.433	0.369	0.356	0.474	0.519	0.484	0.572	0.580
d_{10}	0.100	0.143	0.528	0.505	0.616	0.619	1.000	1.000	1.000	1.000	1.000
d_{11}	0.114	0.121	0.210	0.466	0.919	0.922	1.000	1.000	1.000	1.000	0.991
d_{12}	0.095	0.098	0.239	0.383	0.664	0.701	1.000	1.000	1.000	1.000	0.400
min	0.095	0.098	0.210	0.298	0.274	0.251	0.204	0.236	0.210	0.276	0.279

Supplementary Table 20: Power of the considered testing procedures for the simulation based on discretised multivariate normal scores with the correlations $r_{wd} = 0.8$, $r_{bd} = 0.5$ and $r_{bf} = 0.5$.

Original scoring

Scenario	IRT.PSIF	LM.PSIBPF	PSPRS-10	OLS	GLS	GLS-26	Bonf	MaxT	Simes	Omnibus	Omnibus-dom
d_1	0.334	0.328	0.385	0.383	0.400	0.398	0.263	0.311	0.272	0.357	0.360
d_2	0.491	0.480	0.558	0.557	0.573	0.572	0.413	0.471	0.425	0.531	0.528
d_3	0.639	0.623	0.709	0.706	0.723	0.721	0.553	0.611	0.570	0.672	0.676
d_4	0.152	0.195	0.572	0.577	0.687	0.693	0.999	1.000	0.999	1.000	1.000
d_5	0.134	0.133	0.531	0.559	0.876	0.882	1.000	1.000	1.000	1.000	1.000
d_6	0.853	0.797	0.555	0.553	0.364	0.350	0.860	0.885	0.865	0.902	0.883
d_7	0.141	0.169	0.558	0.555	0.762	0.772	0.902	0.923	0.907	0.936	0.957
d_8	0.564	0.540	0.515	0.514	0.442	0.434	0.494	0.551	0.509	0.603	0.576
d_9	0.650	0.605	0.544	0.540	0.520	0.511	0.626	0.680	0.638	0.721	0.726
d_{10}	0.092	0.107	0.403	0.541	0.949	0.951	1.000	1.000	1.000	1.000	0.996
d_{11}	0.122	0.142	0.416	0.550	0.972	0.972	1.000	1.000	1.000	1.000	1.000
d_{12}	0.094	0.120	0.411	0.546	0.917	0.934	1.000	1.000	1.000	1.000	0.750
min	0.092	0.107	0.385	0.383	0.364	0.350	0.263	0.311	0.272	0.357	0.360

PSPRS-10 scoring

Scenario	IRT.PSIF	LM.PSIBPF	PSPRS-10	OLS	GLS	GLS-26	Bonf	MaxT	Simes	Omnibus	Omnibus-dom
d_1	0.328	0.340	0.372	0.370	0.366	0.362	0.247	0.280	0.253	0.337	0.347
d_2	0.478	0.491	0.538	0.528	0.520	0.517	0.380	0.415	0.390	0.486	0.506
d_3	0.627	0.643	0.691	0.681	0.667	0.663	0.511	0.553	0.524	0.627	0.659
d_4	0.193	0.242	0.537	0.530	0.641	0.662	0.998	0.999	0.999	0.999	1.000
d_5	0.149	0.150	0.538	0.557	0.770	0.791	1.000	1.000	1.000	1.000	1.000
d_6	0.802	0.780	0.532	0.534	0.337	0.304	0.806	0.831	0.814	0.856	0.845
d_7	0.162	0.198	0.526	0.527	0.700	0.722	0.873	0.891	0.878	0.913	0.949
d_8	0.543	0.552	0.495	0.487	0.417	0.404	0.450	0.491	0.460	0.550	0.551
d_9	0.618	0.601	0.536	0.528	0.451	0.434	0.582	0.620	0.592	0.677	0.695
d_{10}	0.119	0.175	0.635	0.605	0.701	0.706	1.000	1.000	1.000	1.000	1.000
d_{11}	0.137	0.146	0.260	0.537	0.936	0.939	1.000	1.000	1.000	1.000	0.999
d_{12}	0.109	0.112	0.299	0.455	0.732	0.756	1.000	1.000	1.000	1.000	0.506
min	0.109	0.112	0.260	0.370	0.337	0.304	0.247	0.280	0.253	0.337	0.347

Supplementary Table 21: Descriptive statistics of the 10 items (with the original scoring) and marginal ANCOVA tests for comparing the respective group (2nd column) against placebo. For the baseline and Week 52 measurements, means and standard errors are reported. The one to the last column shows the treatment effect estimates (with standard error) from the marginal ANCOVA models for each of the items. Negative values correspond to a beneficial effect of treatment.

		Baseline	Week 52	Difference	ANCOVA	P-value
Dysp.FS	til. 2000 mg	0.652(0.073)	0.864(0.089)	0.212(0.082)	0.06(0.104)	0.716
	til. 4000 mg	0.662(0.078)	1.028(0.112)	0.366(0.088)	0.207(0.114)	0.964
	Placebo	0.594(0.076)	0.766(0.088)	0.172(0.072)	–	–
Use.KF	til. 2000 mg	1.591(0.108)	2.242(0.133)	0.652(0.119)	0.001(0.167)	0.503
	til. 4000 mg	1.577(0.098)	2.296(0.116)	0.718(0.105)	0.062(0.156)	0.653
	Placebo	1.578(0.106)	2.234(0.127)	0.656(0.138)	–	–
Fall	til. 2000 mg	2.409(0.126)	2.712(0.155)	0.303(0.146)	−0.149(0.194)	0.221
	til. 4000 mg	2.056(0.122)	2.451(0.151)	0.394(0.137)	−0.229(0.189)	0.114
	Placebo	2.219(0.147)	2.766(0.149)	0.547(0.157)	–	–
Dysa.	til. 2000 mg	1.50(0.104)	2.03(0.124)	0.53(0.099)	0.186(0.133)	0.918
	til. 4000 mg	1.563(0.091)	2.042(0.111)	0.479(0.098)	0.152(0.13)	0.878
	Placebo	1.641(0.098)	1.938(0.104)	0.297(0.099)	–	–
Dysp.	til. 2000 mg	1.197(0.117)	1.439(0.137)	0.242(0.15)	−0.171(0.189)	0.183
	til. 4000 mg	1.028(0.104)	1.521(0.137)	0.493(0.131)	−0.016(0.184)	0.466
	Placebo	1.047(0.123)	1.547(0.148)	0.50(0.149)	–	–
Neck.Ri	til. 2000 mg	1.773(0.101)	2.182(0.128)	0.409(0.108)	−0.011(0.148)	0.471
	til. 4000 mg	1.648(0.108)	2.028(0.125)	0.38(0.097)	−0.07(0.139)	0.308
	Placebo	1.562(0.111)	2.031(0.135)	0.469(0.104)	–	–
Ari.FC	til. 2000 mg	2.076(0.159)	2.879(0.158)	0.803(0.163)	−0.035(0.194)	0.429
	til. 4000 mg	2.183(0.145)	2.873(0.149)	0.69(0.123)	−0.111(0.174)	0.262
	Placebo	2.062(0.157)	2.906(0.165)	0.844(0.145)	–	–
Gait	til. 2000 mg	1.985(0.117)	2.606(0.114)	0.621(0.094)	0.008(0.126)	0.527
	til. 4000 mg	1.944(0.10)	2.451(0.106)	0.507(0.087)	−0.121(0.123)	0.163
	Placebo	1.812(0.111)	2.484(0.118)	0.672(0.10)	–	–
Pos.St	til. 2000 mg	2.242(0.13)	2.879(0.132)	0.636(0.114)	−0.071(0.158)	0.327
	til. 4000 mg	2.056(0.139)	2.761(0.141)	0.704(0.114)	−0.079(0.159)	0.311
	Placebo	2.141(0.141)	2.891(0.139)	0.75(0.135)	–	–
Sit	til. 2000 mg	1.712(0.107)	2.394(0.143)	0.682(0.102)	−0.166(0.155)	0.143
	til. 4000 mg	1.718(0.101)	2.437(0.133)	0.718(0.121)	−0.122(0.162)	0.227
	Placebo	1.672(0.126)	2.531(0.13)	0.859(0.126)	–	–

Supplementary Table 22: Descriptive statistics of the 10 items (with the PSPRS-10 scoring) and marginal ANCOVA tests for comparing the respective group (2nd column) against placebo. Similar to Supplementary Table 21, for the baseline and Week 52 measurements, means and standard errors are reported. The one to the last column shows the treatment effect estimates (with standard error) from the marginal ANCOVA models for each of the items. Negative values correspond to a beneficial effect of treatment.

		Baseline	Week 52	Difference	ANCOVA	P-value
Dysp.FS	til. 2000 mg	0.652(0.073)	0.864(0.089)	0.212(0.082)	0.06(0.104)	0.716
	til. 4000 mg	0.662(0.078)	1.028(0.112)	0.366(0.088)	0.207(0.114)	0.964
	Placebo	0.594(0.076)	0.766(0.088)	0.172(0.072)	–	–
Use.KF	til. 2000 mg	1.561(0.099)	2.106(0.11)	0.545(0.106)	−0.012(0.143)	0.467
	til. 4000 mg	1.563(0.094)	2.211(0.102)	0.648(0.092)	0.093(0.135)	0.754
	Placebo	1.578(0.106)	2.125(0.108)	0.547(0.128)	–	–
Fall	til. 2000 mg	1.167(0.051)	1.348(0.07)	0.182(0.064)	−0.016(0.092)	0.432
	til. 4000 mg	1.042(0.047)	1.268(0.063)	0.225(0.061)	−0.037(0.088)	0.339
	Placebo	1.094(0.062)	1.328(0.071)	0.234(0.076)	–	–
Dysa.	til. 2000 mg	1.015(0.055)	1.303(0.065)	0.288(0.071)	0.09(0.082)	0.865
	til. 4000 mg	1.056(0.049)	1.225(0.061)	0.169(0.063)	−0.004(0.079)	0.482
	Placebo	1.031(0.054)	1.219(0.057)	0.188(0.062)	–	–
Dysp.	til. 2000 mg	1.197(0.117)	1.439(0.137)	0.242(0.15)	−0.171(0.189)	0.183
	til. 4000 mg	1.028(0.104)	1.521(0.137)	0.493(0.131)	−0.016(0.184)	0.466
	Placebo	1.047(0.123)	1.547(0.148)	0.5(0.149)	–	–
Neck.Ri	til. 2000 mg	1.091(0.060)	1.455(0.092)	0.364(0.077)	0.089(0.112)	0.786
	til. 4000 mg	1.127(0.066)	1.338(0.085)	0.211(0.072)	−0.05(0.107)	0.32
	Placebo	1.062(0.066)	1.344(0.092)	0.281(0.085)	–	–
Ari.FC	til. 2000 mg	0.606(0.094)	1.152(0.102)	0.545(0.097)	−0.075(0.127)	0.277
	til. 4000 mg	0.634(0.088)	1.127(0.10)	0.493(0.085)	−0.12(0.122)	0.163
	Placebo	0.562(0.091)	1.203(0.102)	0.641(0.101)	–	–
Gait	til. 2000 mg	1(0.101)	1.455(0.092)	0.455(0.081)	−0.068(0.11)	0.269
	til. 4000 mg	0.958(0.097)	1.338(0.087)	0.38(0.076)	−0.16(0.106)	0.066
	Placebo	0.828(0.101)	1.422(0.102)	0.594(0.094)	–	–
Pos.St	til. 2000 mg	1.303(0.116)	1.894(0.128)	0.591(0.108)	−0.052(0.15)	0.364
	til. 4000 mg	1.155(0.121)	1.817(0.127)	0.662(0.108)	−0.034(0.149)	0.409
	Placebo	1.266(0.114)	1.922(0.13)	0.656(0.118)	–	–
Sit	til. 2000 mg	0.788(0.093)	1.455(0.13)	0.667(0.097)	−0.102(0.15)	0.249
	til. 4000 mg	0.761(0.093)	1.493(0.12)	0.732(0.108)	−0.048(0.152)	0.375
	Placebo	0.797(0.105)	1.562(0.122)	0.766(0.121)	–	–

		IRT-PSIF	LM-PSIBPF	PSPRS-10	OLS	GLS	GLS-26	Bonf	MaxT	Simes	Omnibus	Omnibus-dom
Original scoring	til. 2000 mg vs Placebo	0.30	0.34	0.23	0.40	0.34	0.37	1.00	0.64	0.79	0.73	0.52
	til. 4000 mg vs Placebo	0.29	0.31	0.35	0.41	0.55	0.53	1.00	0.56	0.72	0.64	0.42
PSPRS-10 scoring	til. 2000 mg vs Placebo	0.13	0.18	0.24	0.44	0.54	0.54	1.00	0.73	0.86	0.80	0.55
	til. 4000 mg vs Placebo	0.27	0.27	0.39	0.39	0.60	0.60	0.66	0.39	0.60	0.55	0.45

Supplementary Table 23: One-sided p-values of the hypothesis tests in the re-analysis of the ABBV-8E12 trial using different analysis methods, based on the original scoring and the FDA re-scoring.

stands for the number of endpoints for each treatment group. Additionally, x_{ijk} represents the measurement on the kth endpoint for the jth subject in the ith treatment group. For treatment group i, the vector of observations, $\boldsymbol{x}_{ij} = (x_{ij1}, x_{ij2}, \ldots, x_{ijm})'$, $j = 1, 2, \ldots, n_i$ has mean vector $\boldsymbol{\mu}_i = (\mu_{i1}, \mu_{i2}, \ldots, \mu_{im})'$ and covariance matrix $\boldsymbol{\Sigma}_i$. The covariance matrix $\boldsymbol{\Sigma}_i$ is based on pairwise covariances and is of dimension $m \times m$. Furthermore, $\boldsymbol{\delta} = \boldsymbol{\mu}_2 - \boldsymbol{\mu}_1 = (\delta_1, \delta_2, \ldots, \delta_m)'$ denotes the vector of mean differences between the treatment group and the control group. The interest is to test a one-sided alternative to detect a treatment effect as follows

$$\begin{cases} H_0 : \delta_k \geq 0, \forall k \in \{1, \ldots, m\} \\ H_1 : \exists\ k \in \{1, \ldots, m\} : \ \delta_k \leq 0. \end{cases}$$

This hypothesis test indicates that, under the alternative the test treatment is supposed to have a nonzero effect in at least one of the components of the multivariate endpoint.

3.1 O'Brien's OLS and GLS tests (homoscedastic case)

For the OLS and GLS tests we used similar ideas as presented in [1, 2]. There the t-statistics are based on the vector of mean differences. Here we estimate the t-statistics from the marginal ANCOVA models. We denote by $t_k = \hat{\beta}_k / \mathrm{SE}(\hat{\beta}_k)$ the standardised treatment effect estimate for kth endpoint, in which $\hat{\beta}_k$ is the estimated coefficient of the treatment variable in the marginal ANCOVA model of kth item and $\mathrm{SE}(\hat{\beta}_k)$ indicates the corresponding standard error estimate. The vector of t-statistics is then assumed (under the null) to have a multivariate normal distribution with the zero mean and the correlation matrix $\boldsymbol{R}$ of the treatment coefficients from the marginal ANCOVA models. Assuming that there is a consistent estimate of $\boldsymbol{R}$, O'Brien [1] proposed the OLS test statistic as

$$t_{OLS} = \frac{\boldsymbol{j}'\boldsymbol{t}}{\sqrt{\boldsymbol{j}'\hat{\boldsymbol{R}}\boldsymbol{j}}}. \tag{1}$$

Here $\boldsymbol{j}$ represents a vector of 1's with an appropriate dimension. Additionally, $\boldsymbol{t}$ is the vector of t-statistics, with each component denoted by t_k, described above.

As mentioned in earlier sections, we considered the available data from the ABBV-8E12 study for each of the 10 endpoints at two time points: baseline and week 52. To derive the vector of individual t-statistics in the OLS test statistic (vector of $\boldsymbol{t}$ on the right-hand side of Eq. 1), we fitted multiple marginal models to data for each endpoint. Finally, we calculated Eq. 1 using the estimated correlation matrix $\hat{\boldsymbol{R}}$ of the treatment coefficients from the marginal ANCOVA models, $\hat{\boldsymbol{R}}$. This calculation was performed using the `multcomp` package in `R`. Each t_k is marginally t-distributed (under H_0) with $n_1 + n_2 - 3$ degrees of freedom, where the degrees of freedom are

determined by including two covariates (baseline and treatment) in the fitted marginal ANCOVA models.

Due to correlation between the endpoints in each group, one may prefer a test statistic based on the generalised least squares (GLS) estimate of t-statistics. In this case, the corresponding GLS test statistic is represented as [1]

$$t_{GLS} = \frac{\boldsymbol{j}'\hat{\boldsymbol{R}}^{-1}\boldsymbol{t}}{\sqrt{\boldsymbol{j}'\hat{\boldsymbol{R}}^{-1}\boldsymbol{j}}}. \tag{2}$$

Both the OLS and GLS test statistics are weighted sums of individual test statistics for m endpoints. The former uses equal weights, while the latter employs unequal weights determined by $\hat{\boldsymbol{R}}^{-1}$. For small samples, the OLS test statistic is approximated by the t-distribution with $n_1 + n_2 - 2m$ d.f. We have employed the modification, presented in [2], as mentioned in Subsection 3.2 using $0.5(n1 + n2 - 3)(1 + 1/m^2)$.

4 IRT model estimation

An item response model describes the relationship between the underlying latent trait and the probability of a certain response (score) for a specific item. The model assumes that the questionnaire/data in hand measures a latent variable, and depending on the number of parameters used in the model, the assumption is that the latent variable prediction is dependent on those parameters and is estimated simultaneously along with those parameters using an estimation algorithm. We have considered a two parameter GR model, which is usually used to model graded items. It assumes that the item difficulty and its discrimination capacity directly affects the probability of a certain score, which in turn is linked to a certain latent variable. The probability for a subject i to have at least a score of s for item j is [3]:

$$P(y_{ij} \geq s) = \frac{1}{1 + e^{a_j(b_{j,s} - \psi_i)}}, \tag{3}$$

where a_j denotes the discrimination parameter and $b_{j,s}$ is the difficulty parameter for the s-th step of the item. Besides ψ_i is the representation of the unobserved latent trait. The discrimination parameter a quantifies how well an item differentiates between individuals with different levels of the latent trait being measured. The difficulty parameters $b_1 - b_4$ indicate the level of the latent trait at which an individual is equally likely to choose one response category over another. The probability to have a certain score s is calculated as

$$P(y_{ij} = s) = P(y_{ij} \geq s) - P(y_{ij} \geq s + 1).$$

Additionally $P(y_{ij} \geq 0)$=1 and $P(y_{ij} \geq S_j + 1) = 0$ hold.

Using the aggregated data at the baseline and follow up visit of the PSPRS-10 scale from the ABBV-8E12 trial, the estimated parameters for the GR model, Eq. (3), are presented in the following table.

Supplementary Table 24: Estimation of the IRT model parameters based on the ABBV-8E12 trial dataset, where the aggregated data from all treatment groups and visits are used to fit the IRT model.

	Original scoring					PSPRS-10 scoring				
	a	b_1	b_2	b_3	b_4	a	b_1	b_2	b_3	b_4
Dysp.FS	0.918	−0.558	2.581	4.439	6.555	0.918	−0.554	2.586	4.436	6.537
Use.KF	1.673	−2.126	−0.332	0.752	2.423	1.677	−2.132	−0.328	0.750	–
Fall	0.919	−3.510	−1.491	0.239	1.453	1.197	−2.894	1.243	–	–
Dysa.	1.118	−2.806	−0.400	1.765	3.524	1.152	−2.765	1.759	–	–
Dysp.	0.942	−1.000	0.561	1.969	5.845	0.929	−1.009	0.571	1.994	5.905
Neck.Ri	0.967	−2.740	−0.701	1.234	3.437	1.013	−2.656	1.204	3.314	–
Ari.FC	3.370	−1.439	−0.541	−0.253	0.674	3.315	−0.244	0.682	–	–
Gait	3.772	−2.051	−0.669	0.131	1.854	4.132	−0.648	0.132	–	–
Pos.St	2.429	−1.685	−0.940	−0.185	0.839	2.524	−0.920	−0.176	0.831	–
Sit	3.420	−1.558	−0.439	0.424	1.577	3.316	−0.434	0.432	1.591	–

References

[1] P. C. O'Brien, "Procedures for comparing samples with multiple endpoints," *Biometrics*, pp. 1079–1087, 1984.

[2] B. R. Logan and A. C. Tamhane, "On O'Brien's OLS and GLS tests for multiple endpoints," *Lecture Notes-Monograph Series*, pp. 76–88, 2004.

[3] S. Ueckert, "Modeling composite assessment data using item response theory," *CPT: Pharmacometrics & Systems Pharmacology*, vol. 7, no. 4, pp. 205–218, 2018.

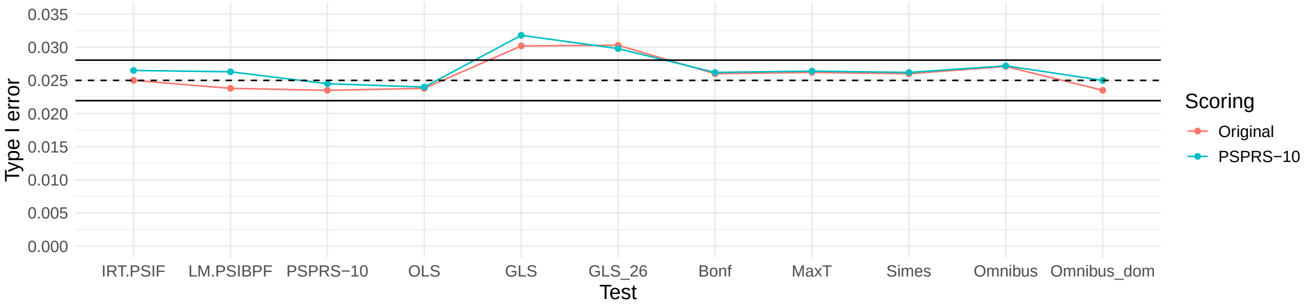

(a) $r_{wd} = 0$, $r_{bd} = 0$ and $r_{bf} = 0$

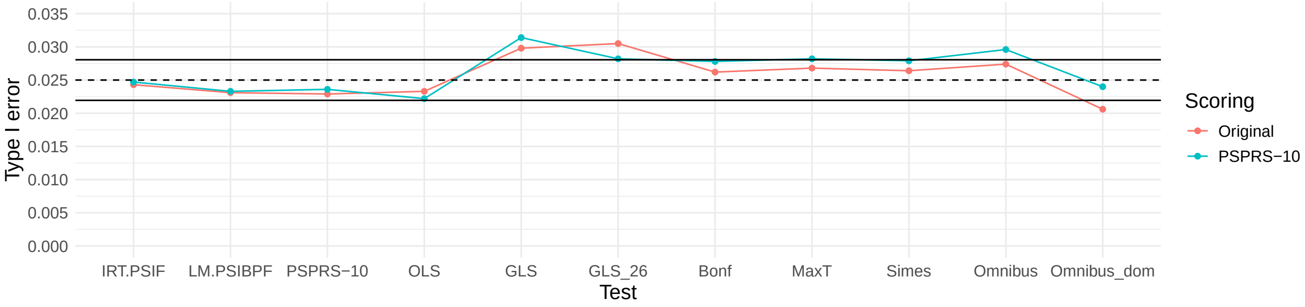

(b) $r_{wd} = 0$, $r_{bd} = 0$ and $r_{bf} = 0.5$

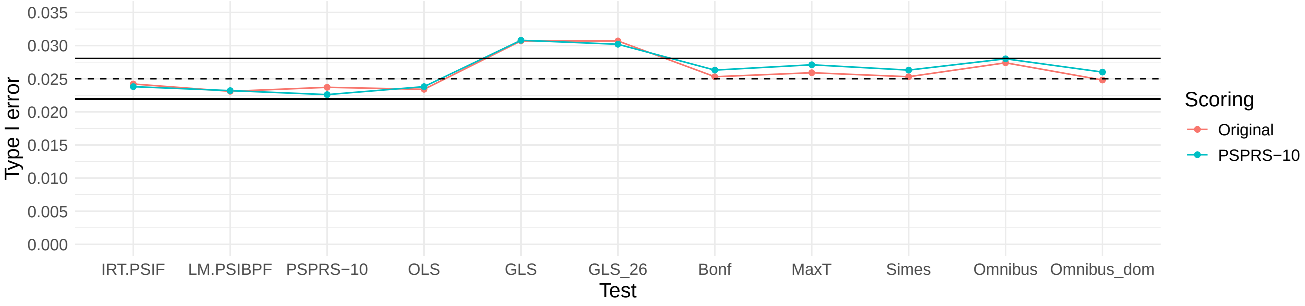

(c) $r_{wd} = 0.1$, $r_{bd} = 0.1$ and $r_{bf} = 0$

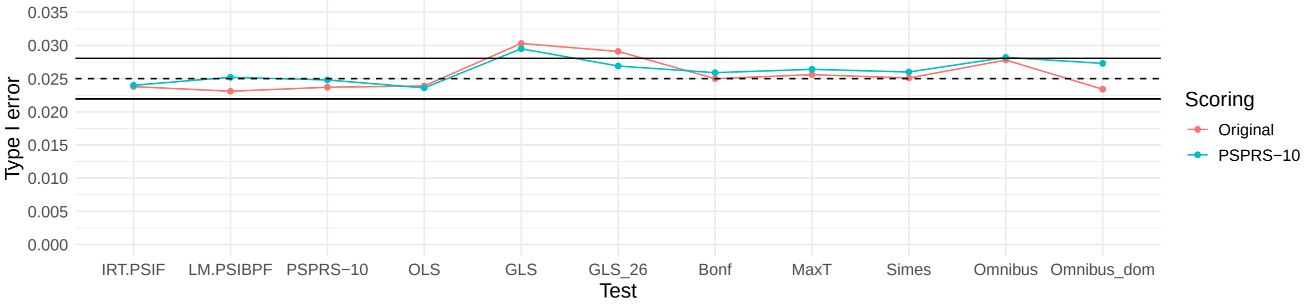

(d) $r_{wd} = 0.1$, $r_{bd} = 0.1$ and $r_{bf} = 0.5$

Supplementary Figure 4: Type I error rates of the hypothesis tests for the simulations with modified correlation matrices. The dashed lines represent the nominal significance level of 0.025 (one-sided). The black solid lines represent the 95% prediction limits (0.02194, 0.02806) for the estimated type I error rate from 10,000 simulation runs when the actual type I error is 0.025.

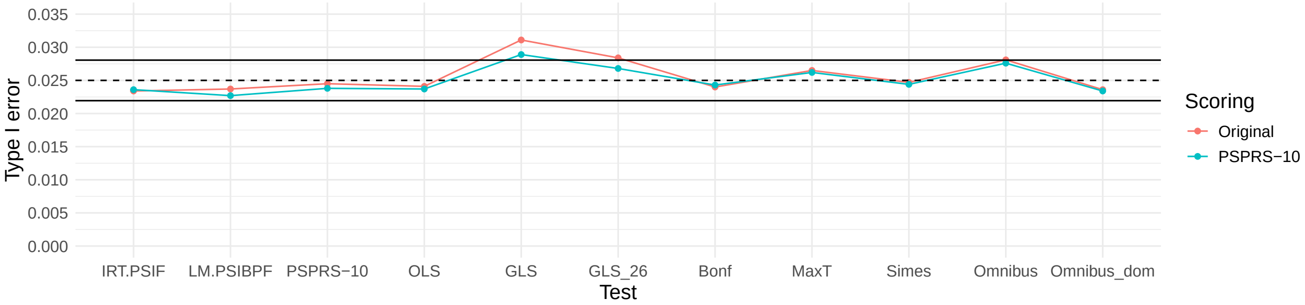

(a) $r_{wd} = 0.5$, $r_{bd} = 0.1$ and $r_{bf} = 0$

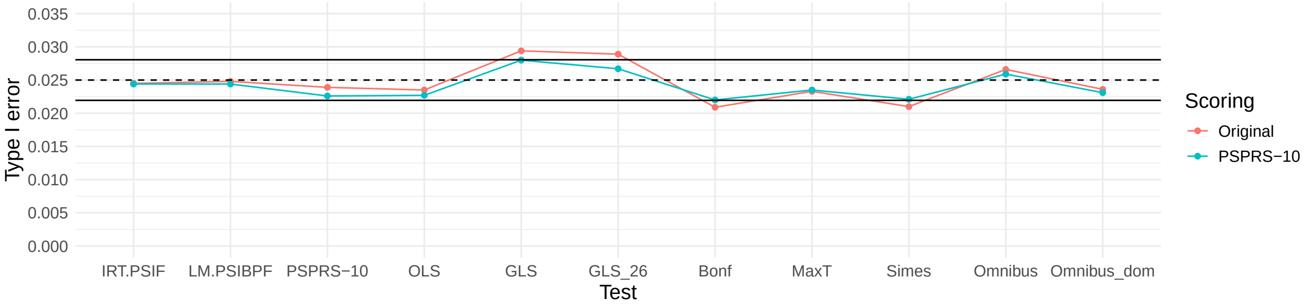

(b) $r_{wd} = 0.5$, $r_{bd} = 0.1$ and $r_{bf} = 0.5$

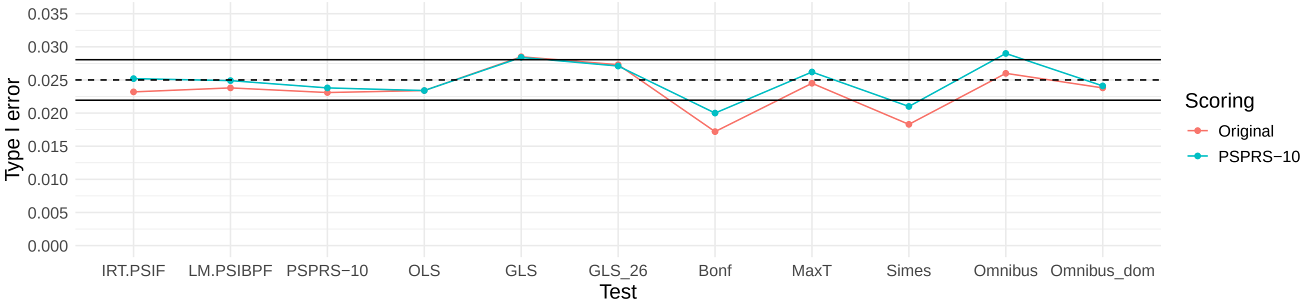

(c) $r_{wd} = 0.8$, $r_{bd} = 0.5$ and $r_{bf} = 0$

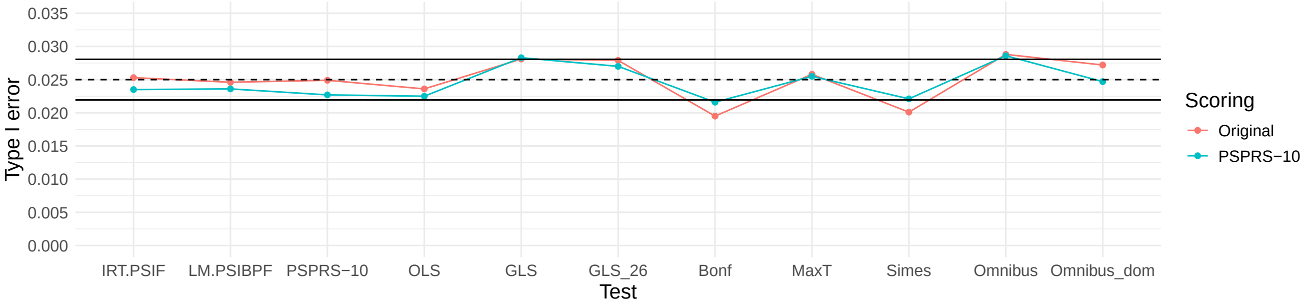

(d) $r_{wd} = 0.8$, $r_{bd} = 0.5$ and $r_{bf} = 0.5$

Supplementary Figure 5: Type I error rates of the hypothesis tests for the simulations with modified correlation matrices. The dashed lines represent the nominal significance level of 0.025 (one-sided). The black solid lines represent the 95% prediction limits (0.02194, 0.02806) for the estimated type I error rate from 10,000 simulation runs when the actual type I error is 0.025.

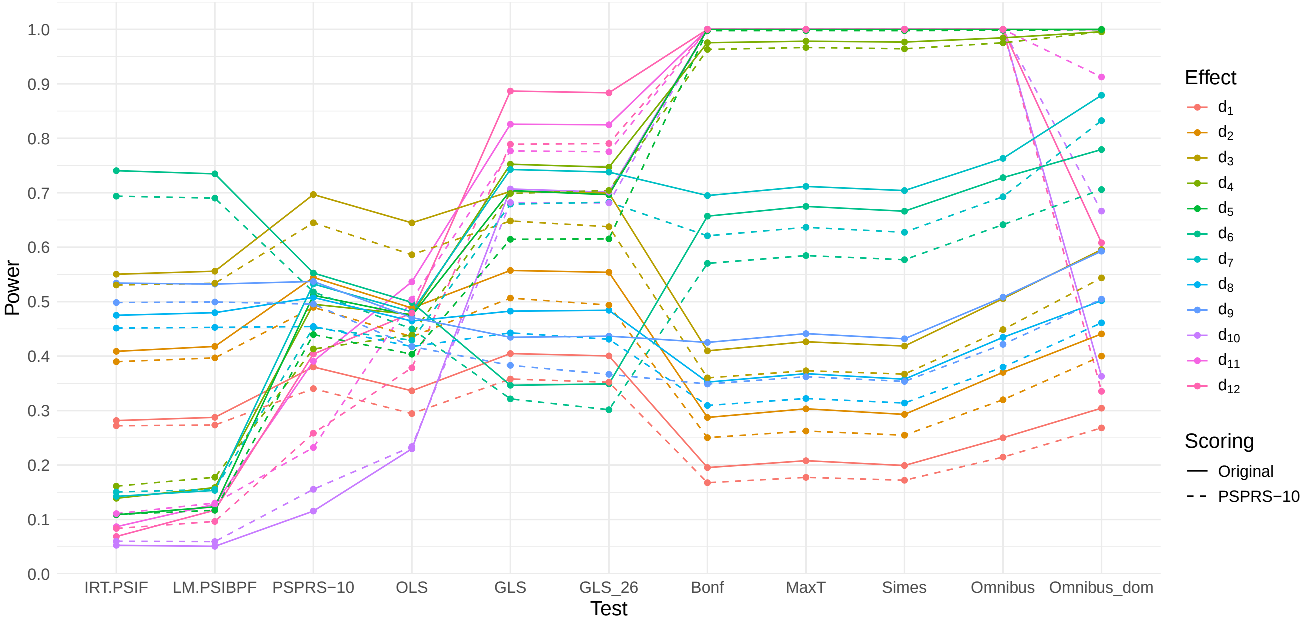

Supplementary Figure 6: Power of the considered testing procedures for the simulation based on discretised multivariate normal scores, $n = 35$ with the original effect size scenarios $\boldsymbol{d}$ as defined in Table 4 in the main manuscript.

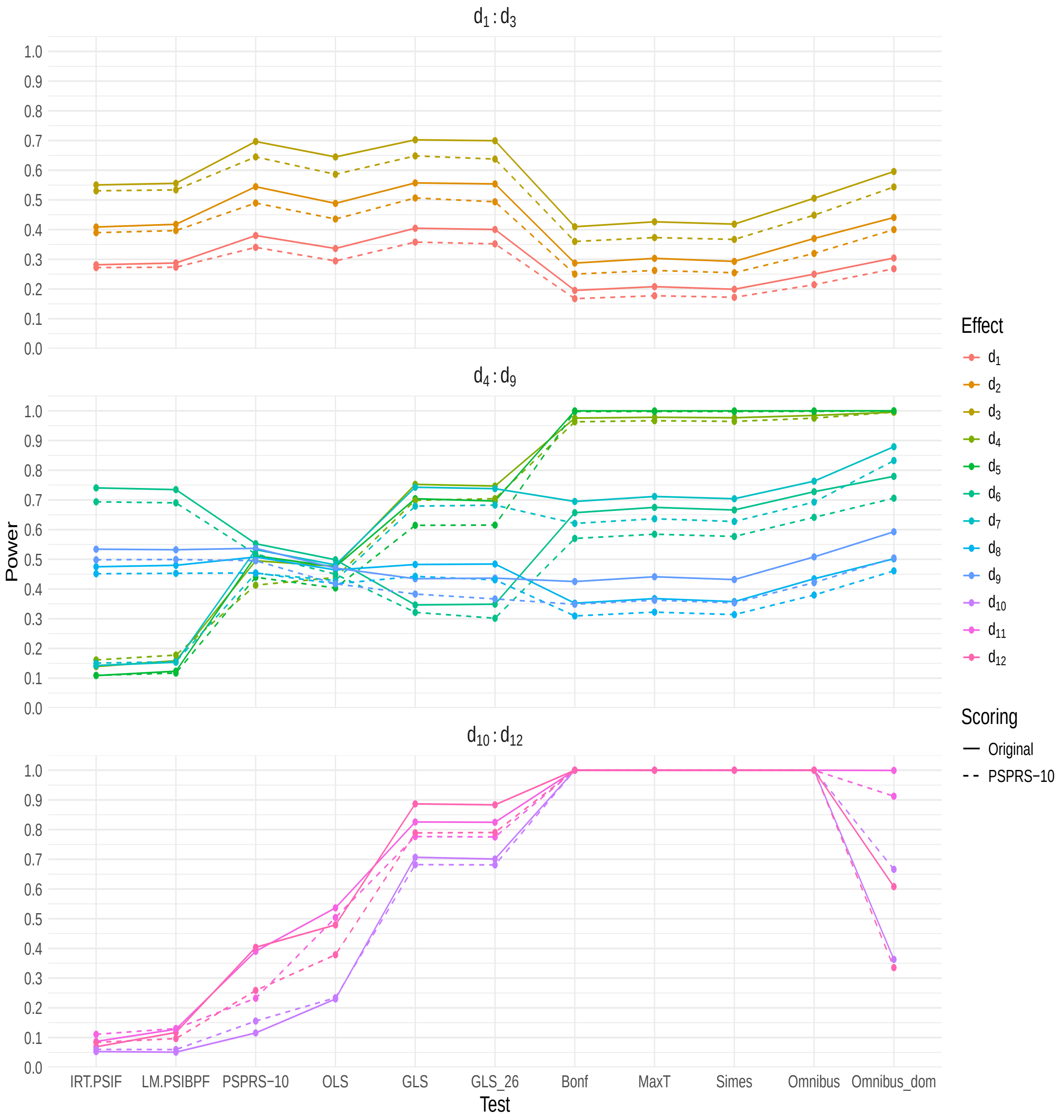

Supplementary Figure 7: Power of the considered testing procedures for the simulation based on discretised multivariate normal scores, $n = 35$ with the original effect size scenarios $\boldsymbol{d}$ as defined in Table 4 in the main manuscript.

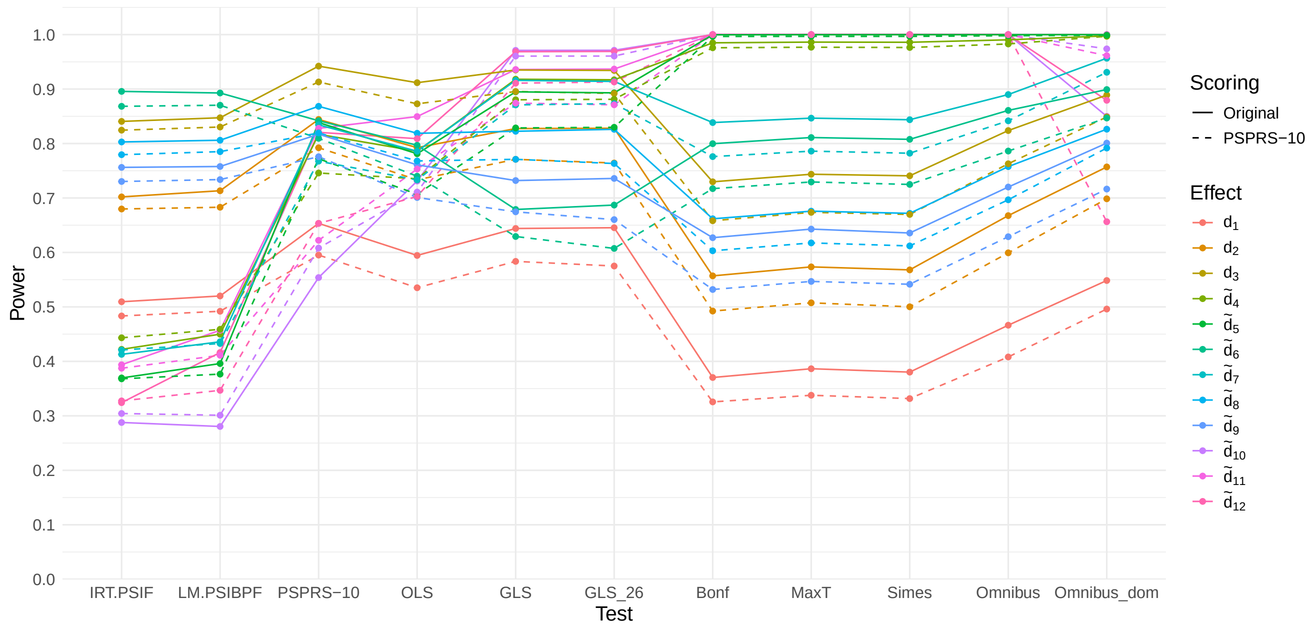

Supplementary Figure 8: Power of the considered testing procedures for the simulation based on discretised multivariate normal scores, $n = 70$ with modified effect size scenarios $\tilde{\boldsymbol{d}}$ defined in Supplementary Table 7.

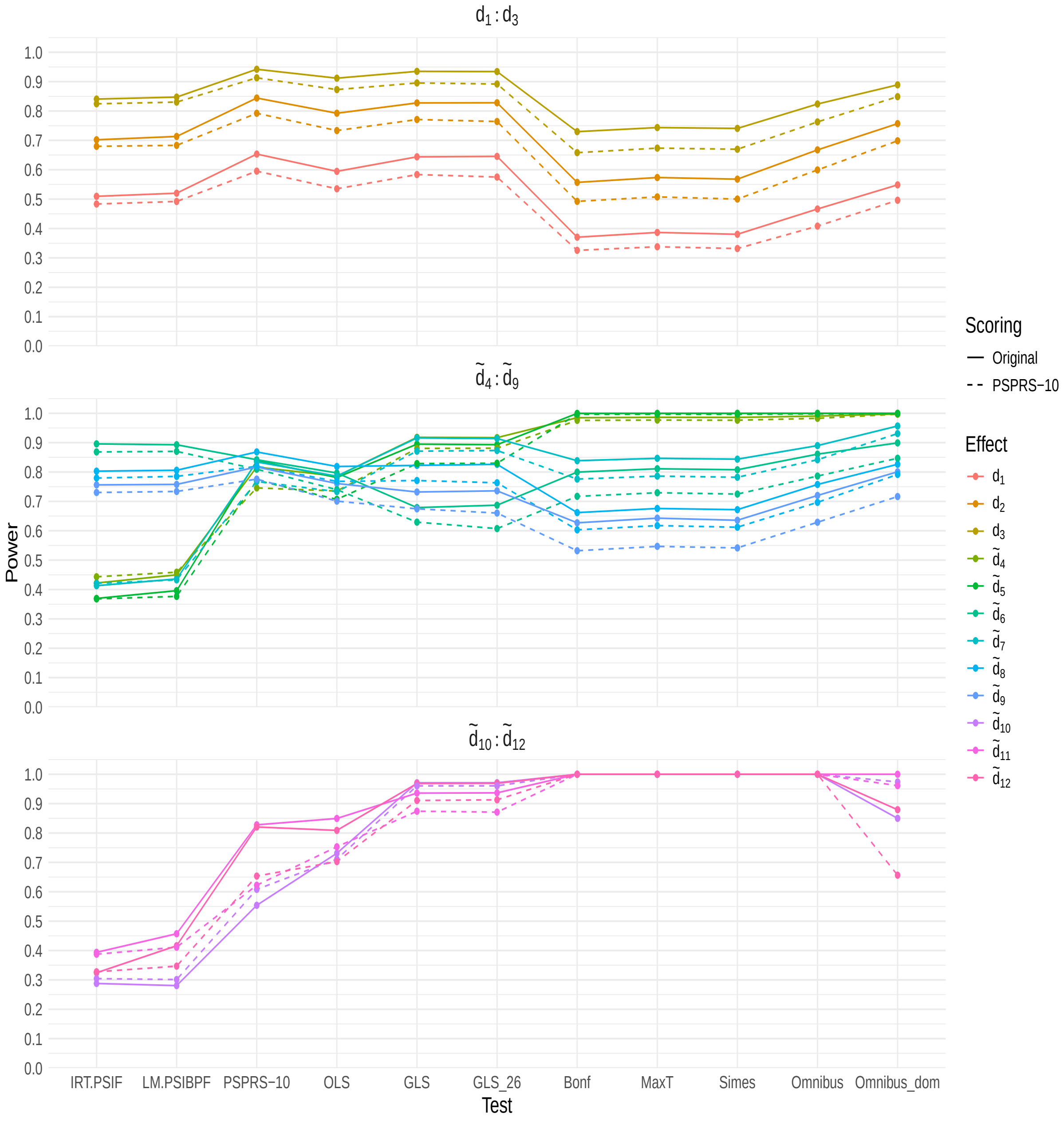

Supplementary Figure 9: Power of the considered testing procedures for the simulation based on discretised multivariate normal scores, $n = 70$ with modified effect size scenarios $\tilde{\boldsymbol{d}}$ defined in Supplementary Table 7.

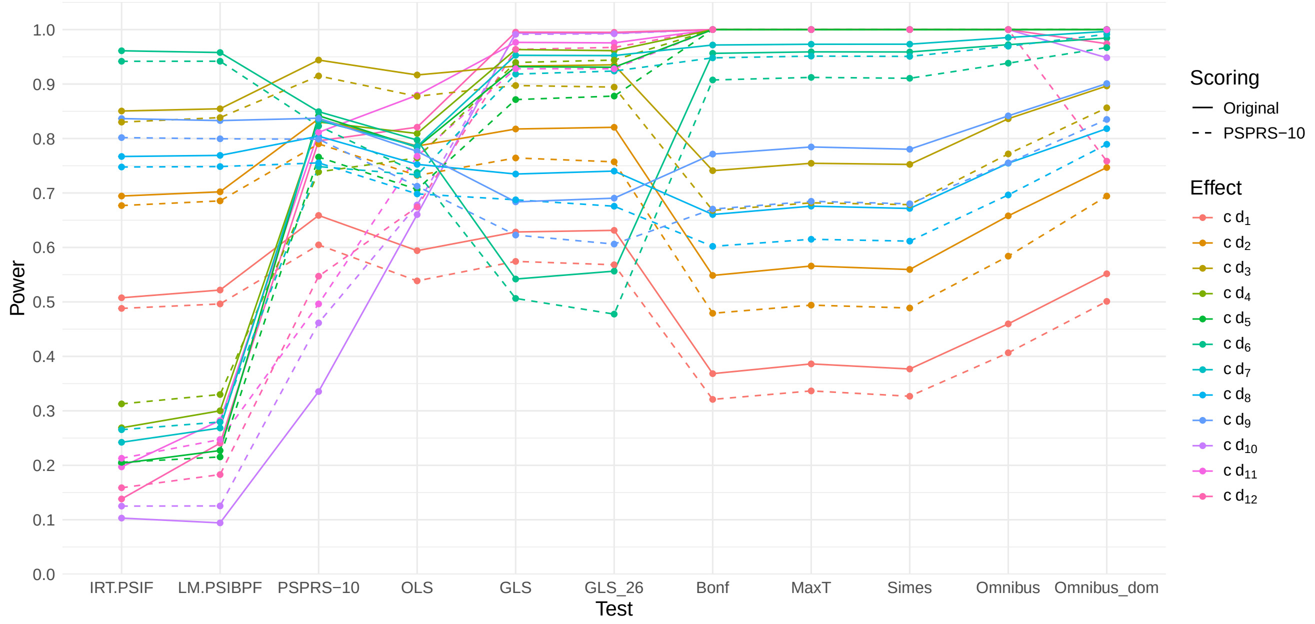

Supplementary Figure 10: Power of the considered testing procedures for the simulation based on discretised multivariate normal scores, $n = 140$ with rescaled effect size scenarios $c\boldsymbol{d}$, where $\boldsymbol{d}$ is defined in the main manuscript in Table 4 and $c = \sqrt{70}/\sqrt{140}$.

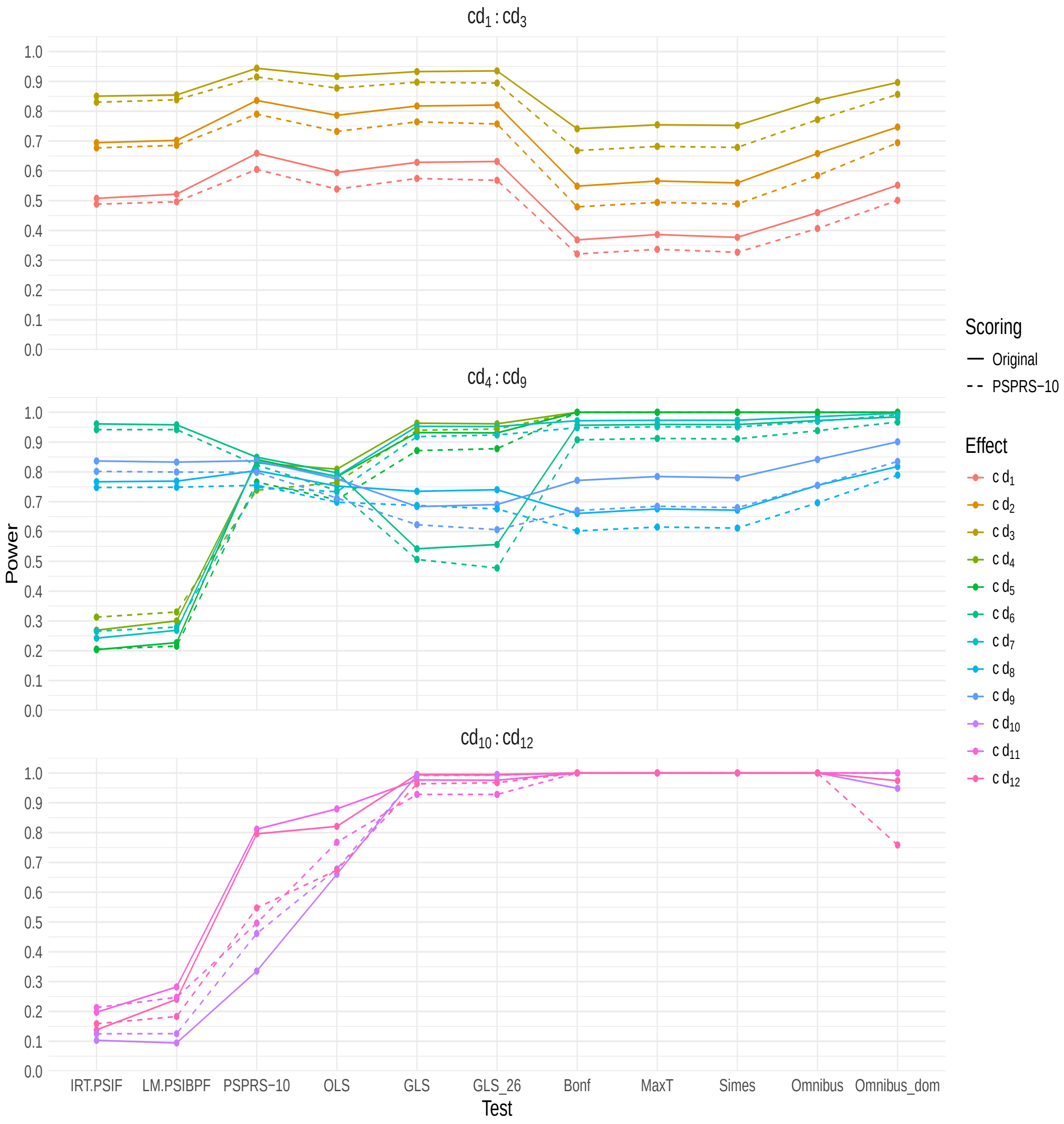

Supplementary Figure 11: Power of the considered testing procedures for the simulation based on discretised multivariate normal scores, $n = 140$ with the rescaled effect size scenarios $c\boldsymbol{d}$, where $\boldsymbol{d}$ is defined in Table 4 in the main manuscript and $c = \sqrt{70}/\sqrt{140}$.

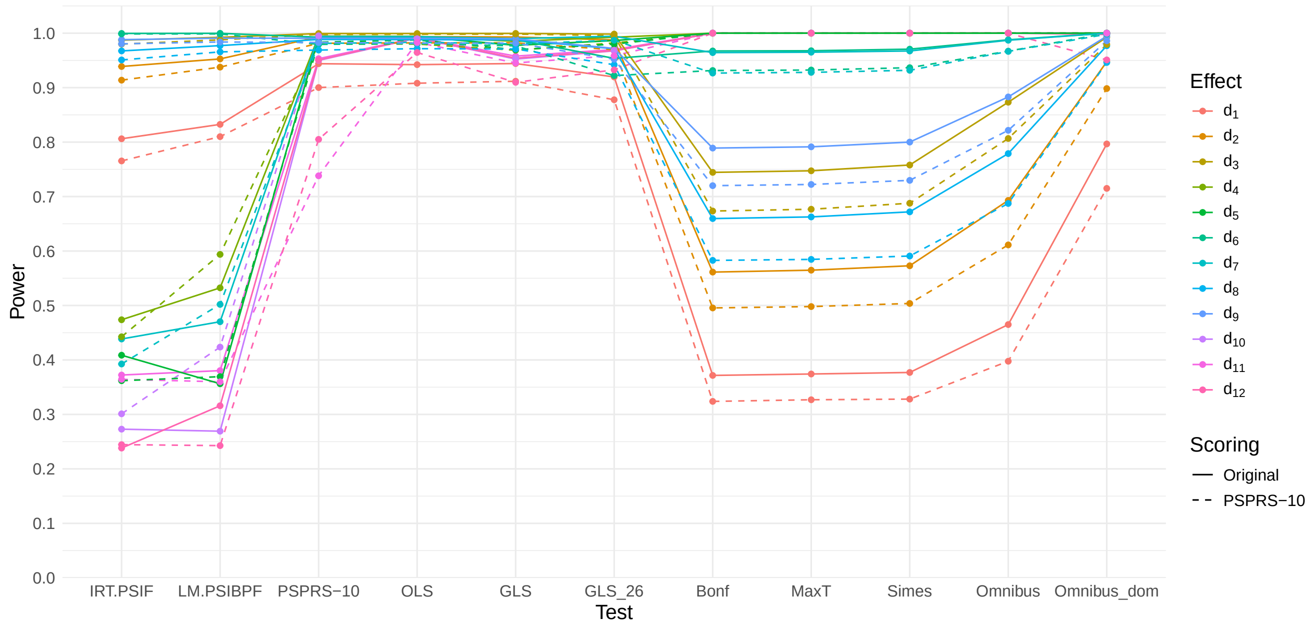

Supplementary Figure 12: Power of the considered testing procedures for the simulation based on discretised multivariate normal scores with the correlations $r_{wd} = 0$, $r_{bd} = 0$ and $r_{bf} = 0$.

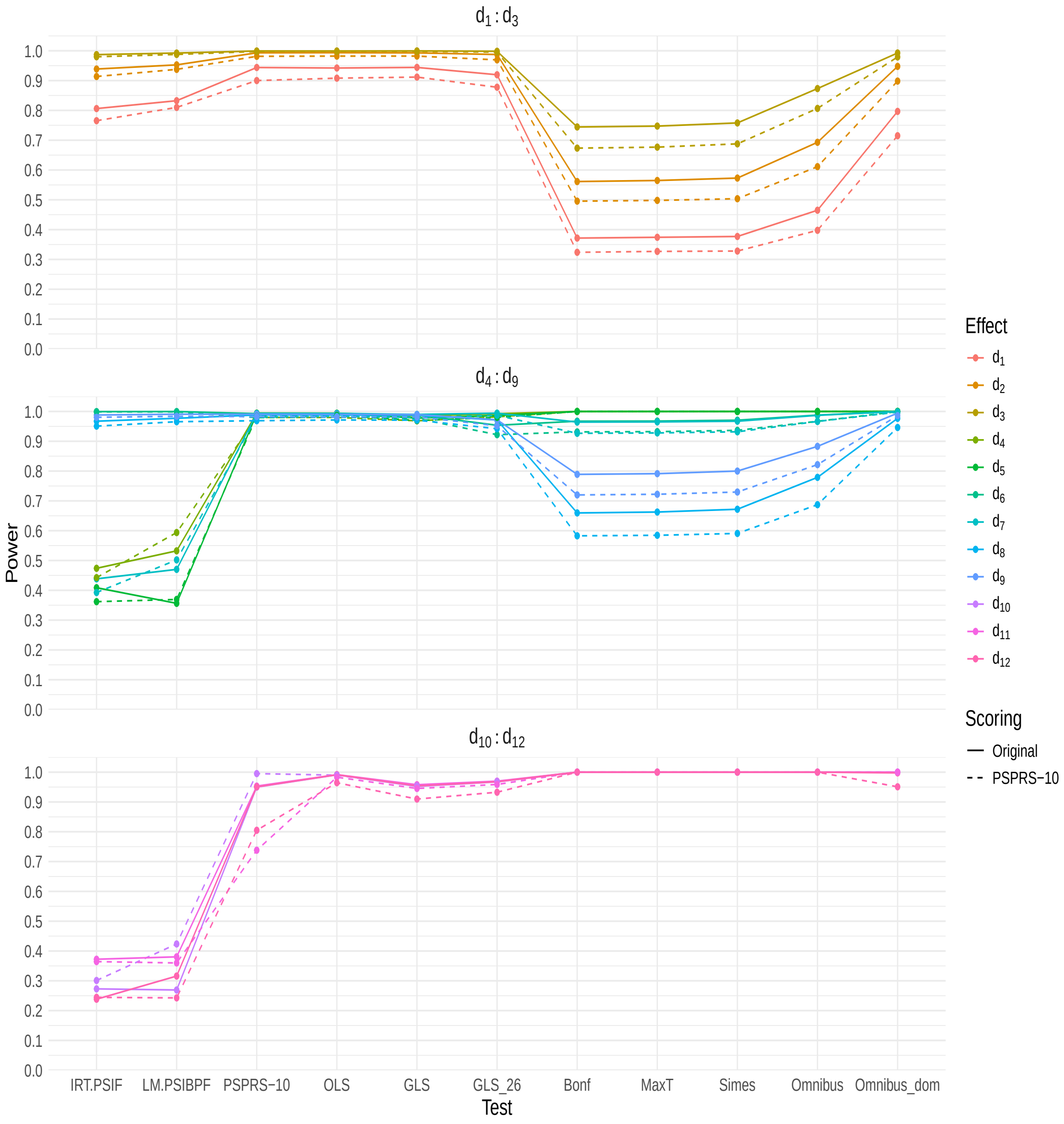

Supplementary Figure 13: Power of the considered testing procedures for the simulation based on discretised multivariate normal scores with the correlations $r_{wd} = 0$, $r_{bd} = 0$ and $r_{bf} = 0$.

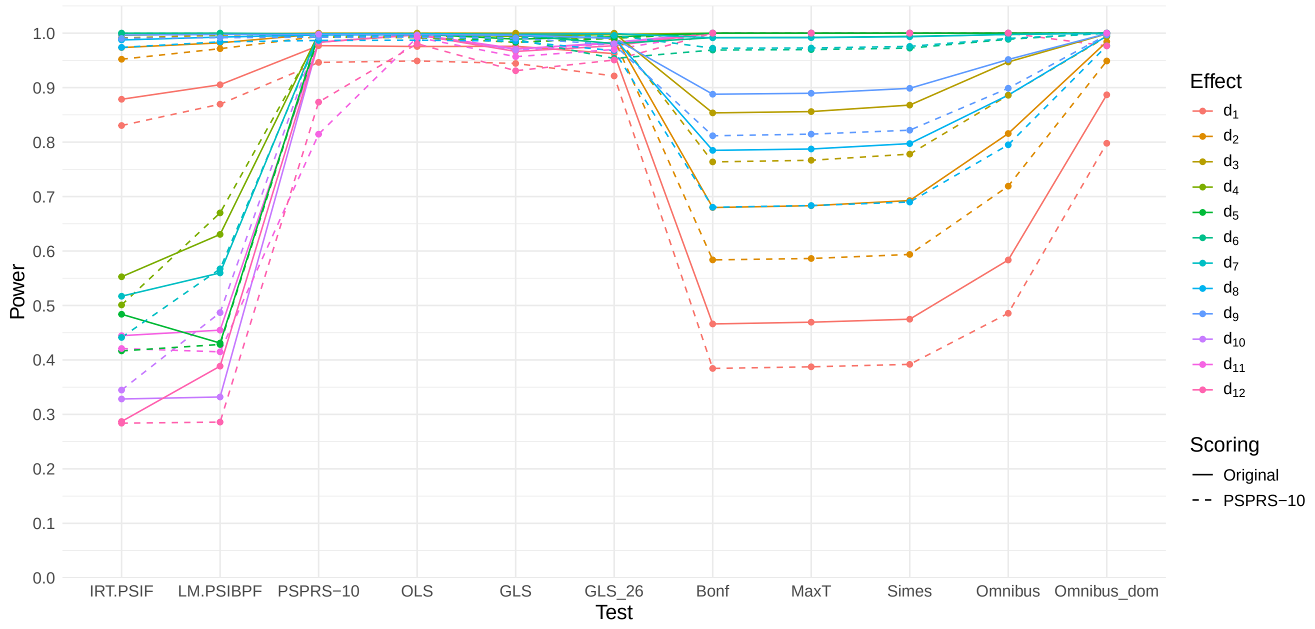

Supplementary Figure 14: Power of the considered testing procedures for the simulation based on discretised multivariate normal scores with the correlations $r_{wd} = 0$, $r_{bd} = 0$ and $r_{bf} = 0.5$.

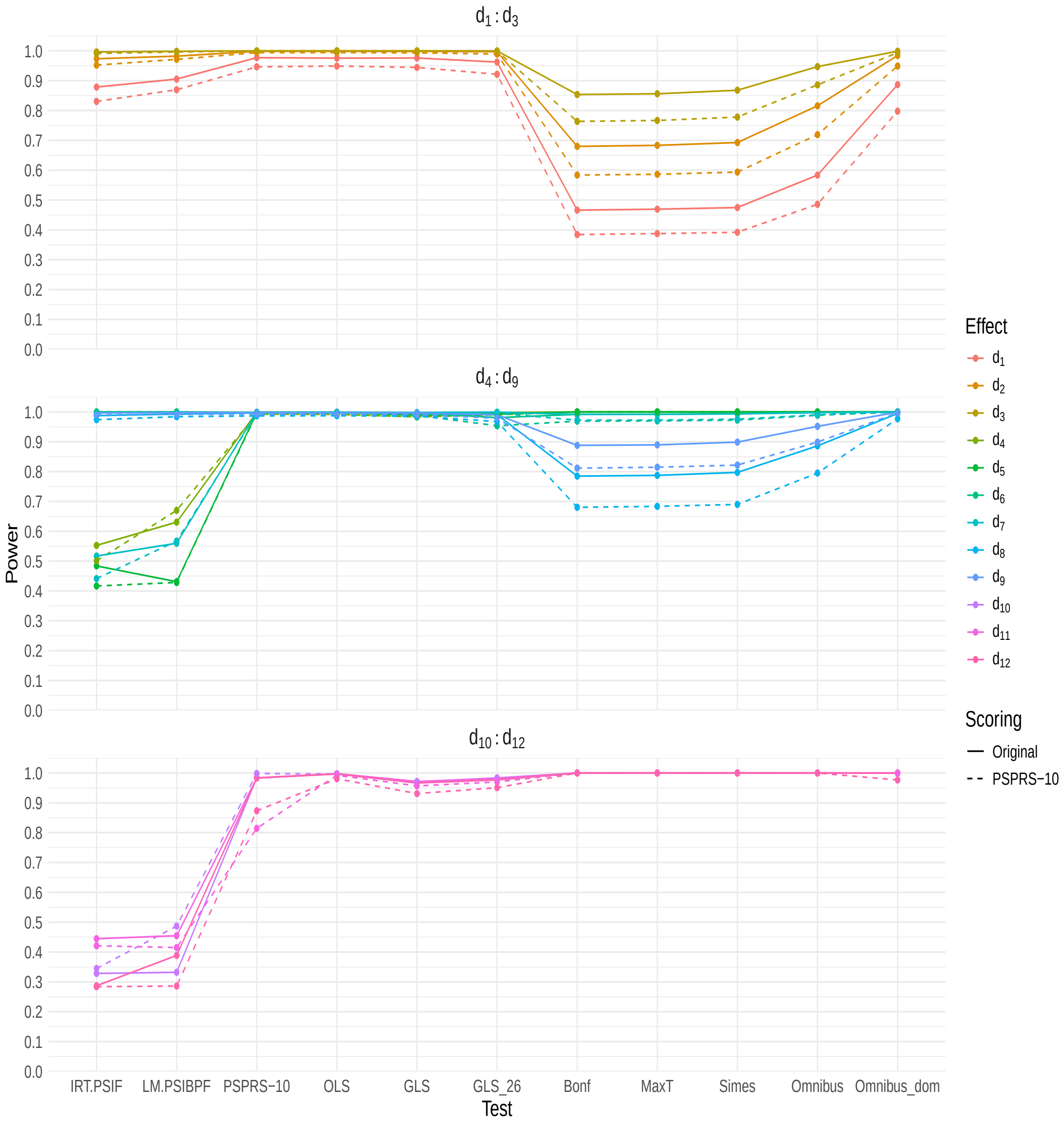

Supplementary Figure 15: Power of the considered testing procedures for the simulation based on discretised multivariate normal scores with the correlations $r_{wd} = 0$, $r_{bd} = 0$ and $r_{bf} = 0.5$.

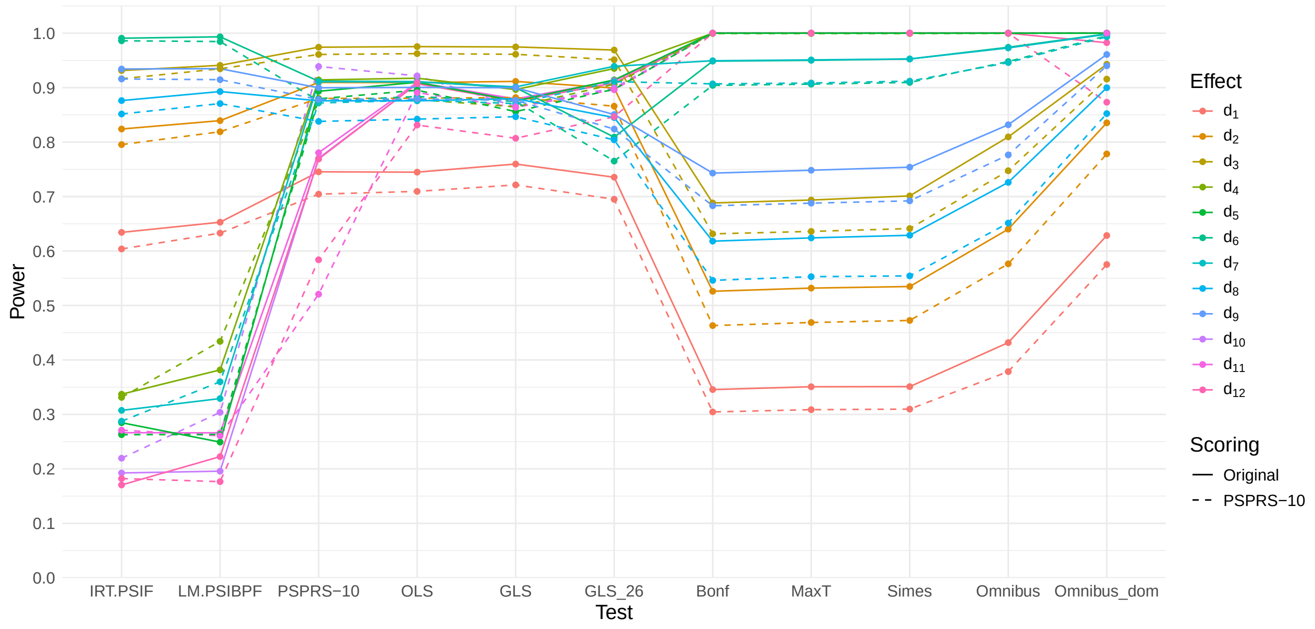

Supplementary Figure 16: Power of the considered testing procedures for the simulation based on discretised multivariate normal scores with the correlations $r_{wd} = 0.1$, $r_{bd} = 0.1$ and $r_{bf} = 0$.

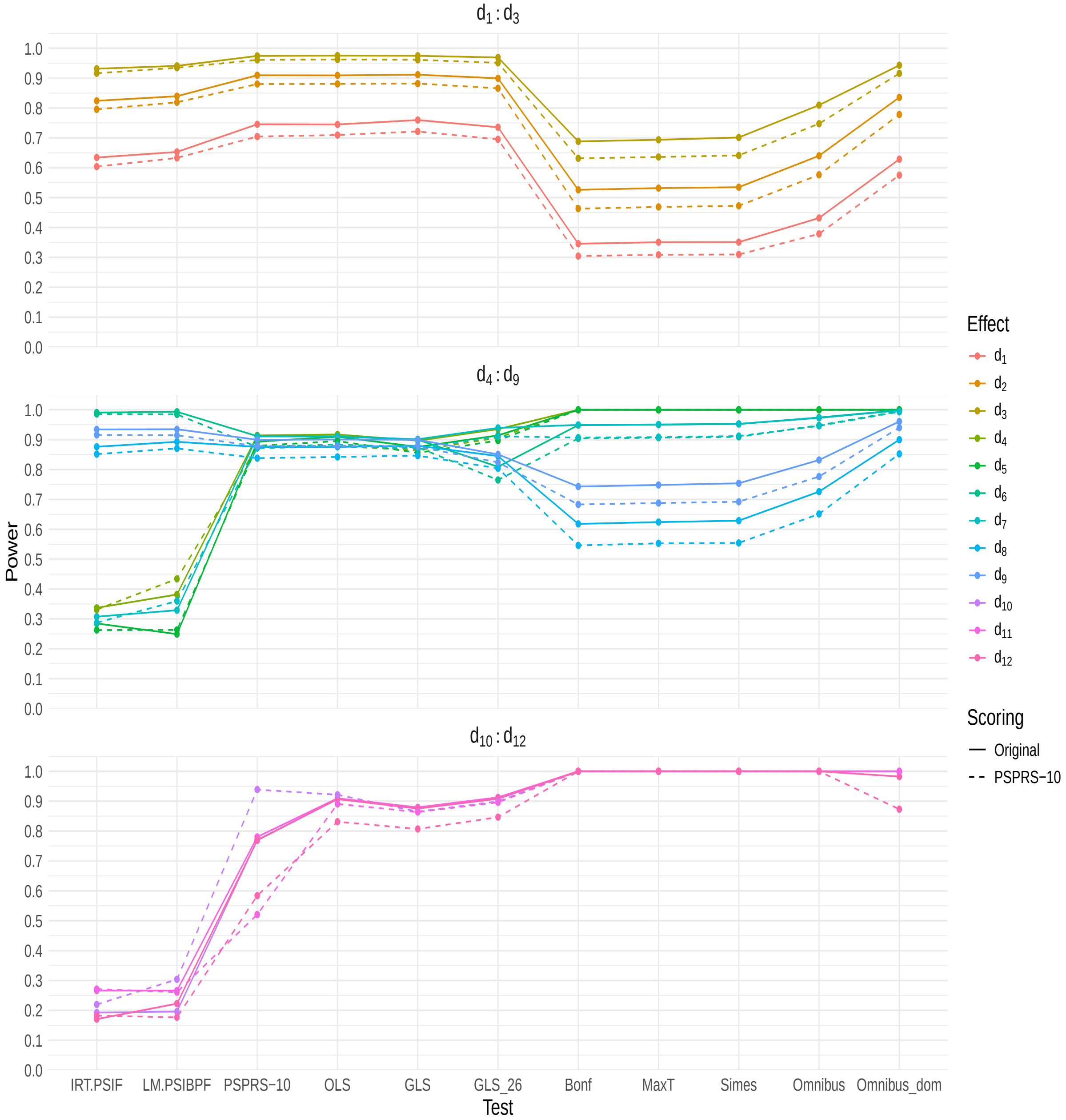

Supplementary Figure 17: Power of the considered testing procedures for the simulation based on discretised multivariate normal scores with the correlations $r_{wd} = 0.1$, $r_{bd} = 0.1$ and $r_{bf} = 0$.

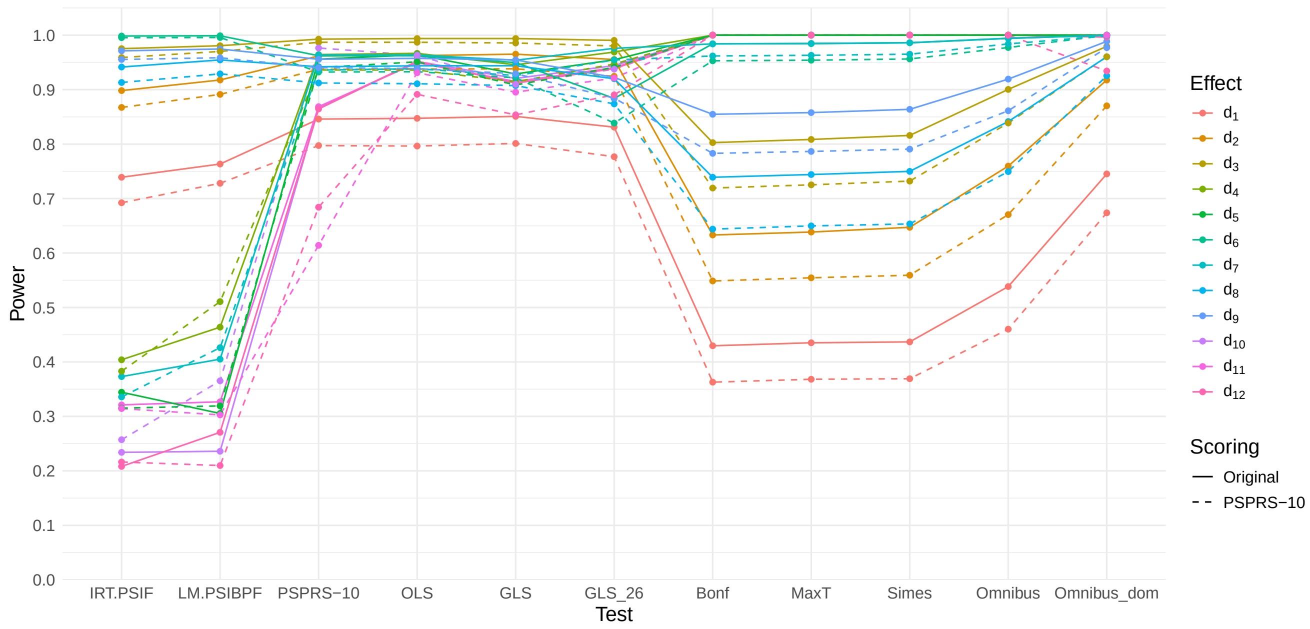

Supplementary Figure 18: Power of the considered testing procedures for the simulation based on discretised multivariate normal scores with the correlations $r_{wd} = 0.1$, $r_{bd} = 0.1$ and $r_{bf} = 0.5$.

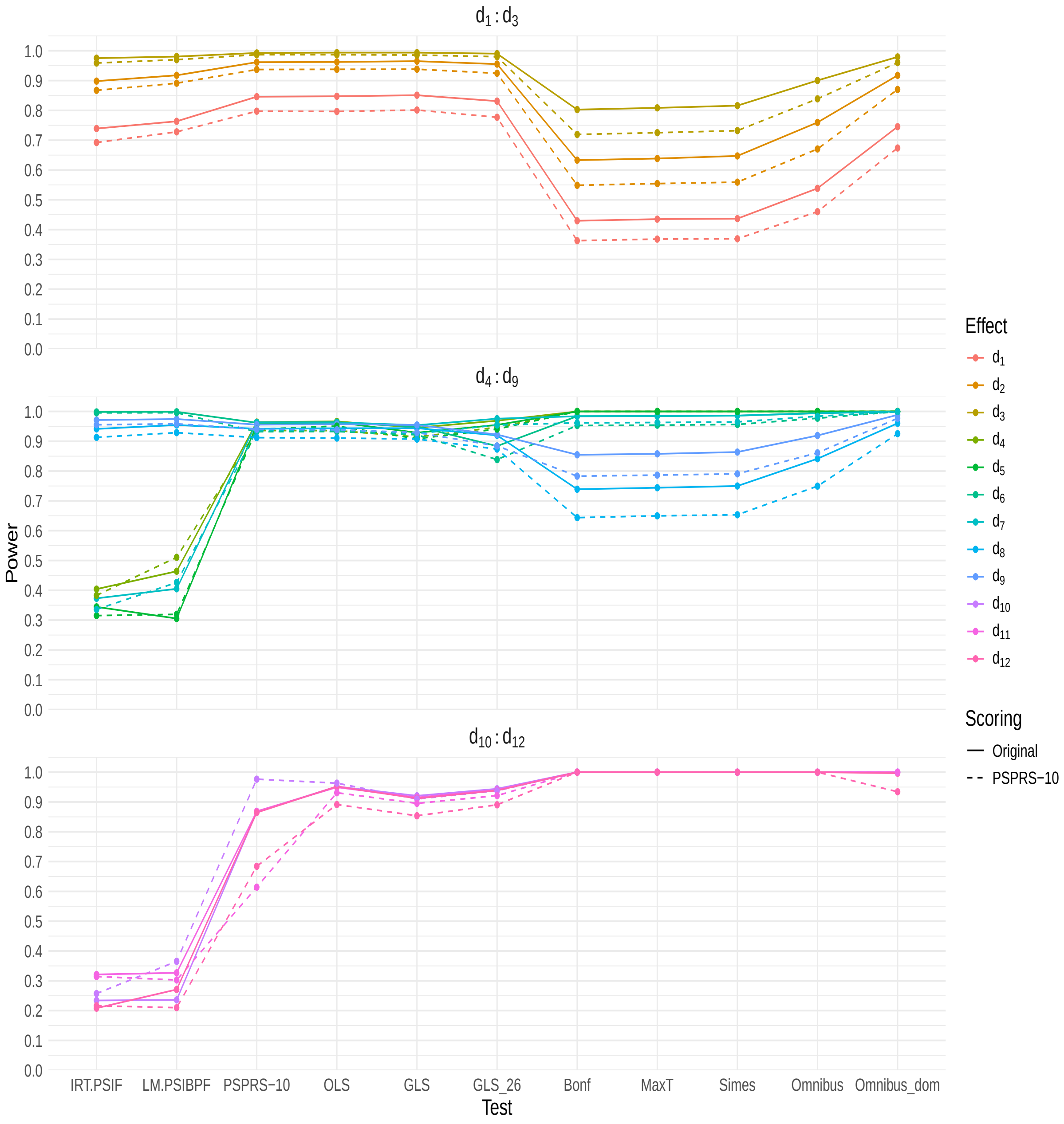

Supplementary Figure 19: Power of the considered testing procedures for the simulation based on discretised multivariate normal scores with the correlations $r_{wd} = 0.1$, $r_{bd} = 0.1$ and $r_{bf} = 0.5$.

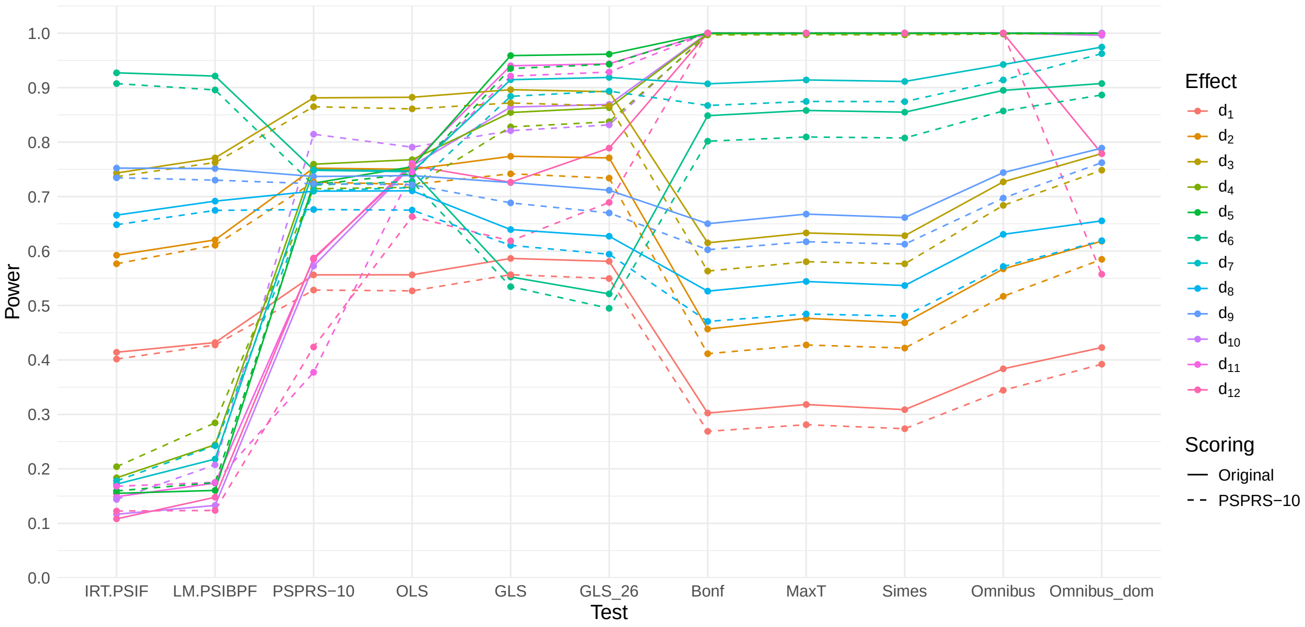

Supplementary Figure 20: Power of the considered testing procedures for the simulation based on discretised multivariate normal scores with the correlations $r_{wd} = 0.5$, $r_{bd} = 0.1$ and $r_{bf} = 0$.

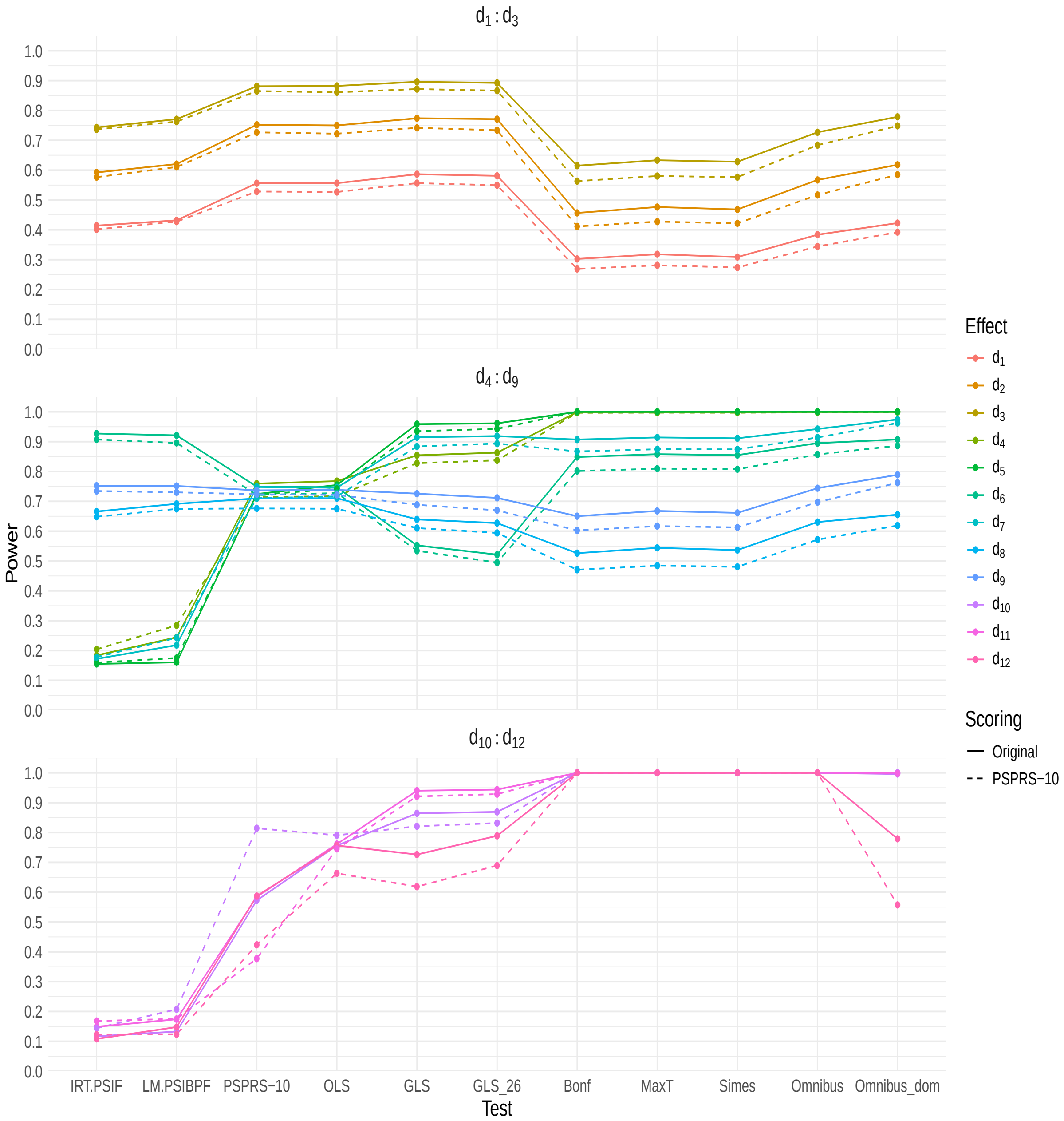

Supplementary Figure 21: Power of the considered testing procedures for the simulation based on discretised multivariate normal scores with the correlations $r_{wd} = 0.5$, $r_{bd} = 0.1$ and $r_{bf} = 0$.

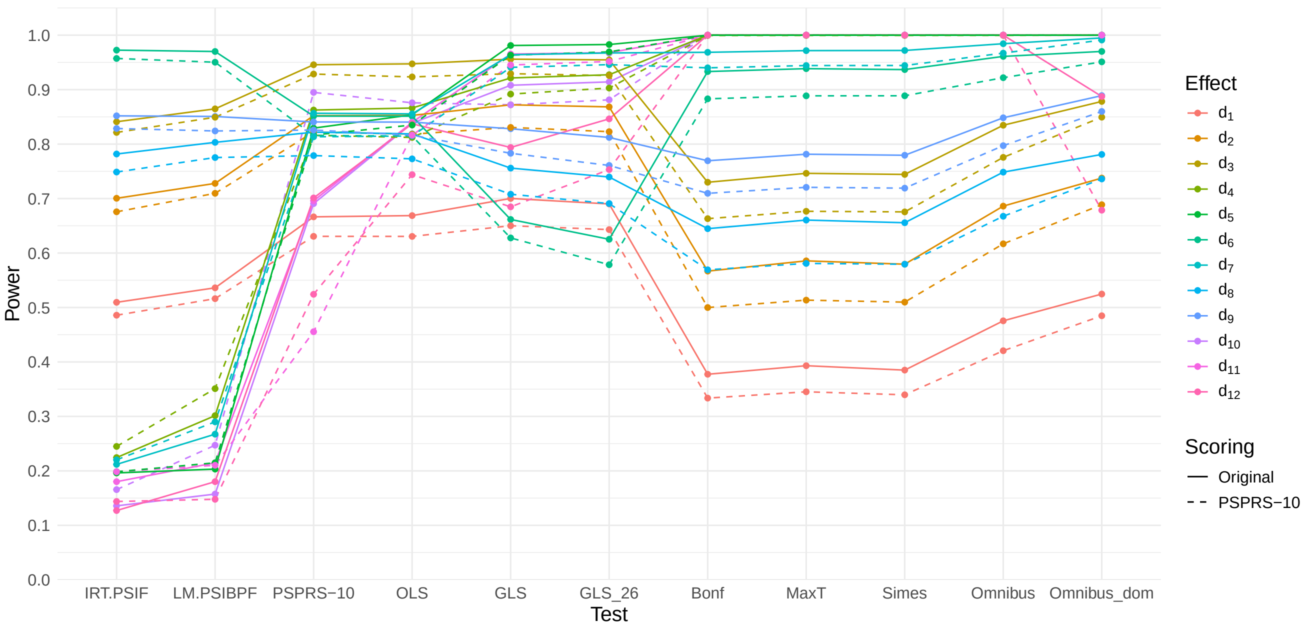

Supplementary Figure 22: Power of the considered testing procedures for the simulation based on discretised multivariate normal scores with the correlations $r_{wd} = 0.5$, $r_{bd} = 0.1$ and $r_{bf} = 0.5$.

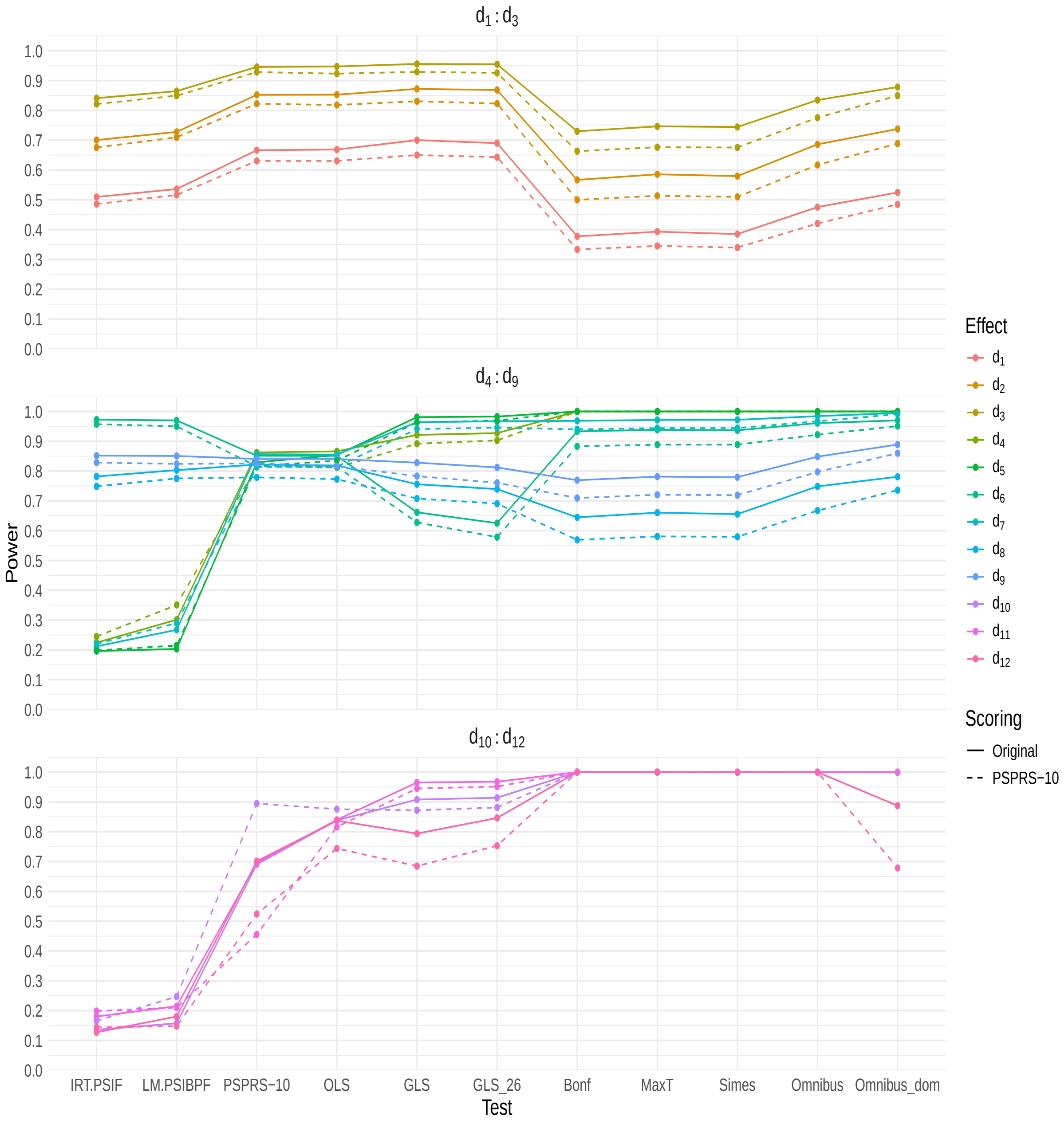

Supplementary Figure 23: Power of the considered testing procedures for the simulation based on discretised multivariate normal scores with the correlations $r_{wd} = 0.5$, $r_{bd} = 0.1$ and $r_{bf} = 0.5$.

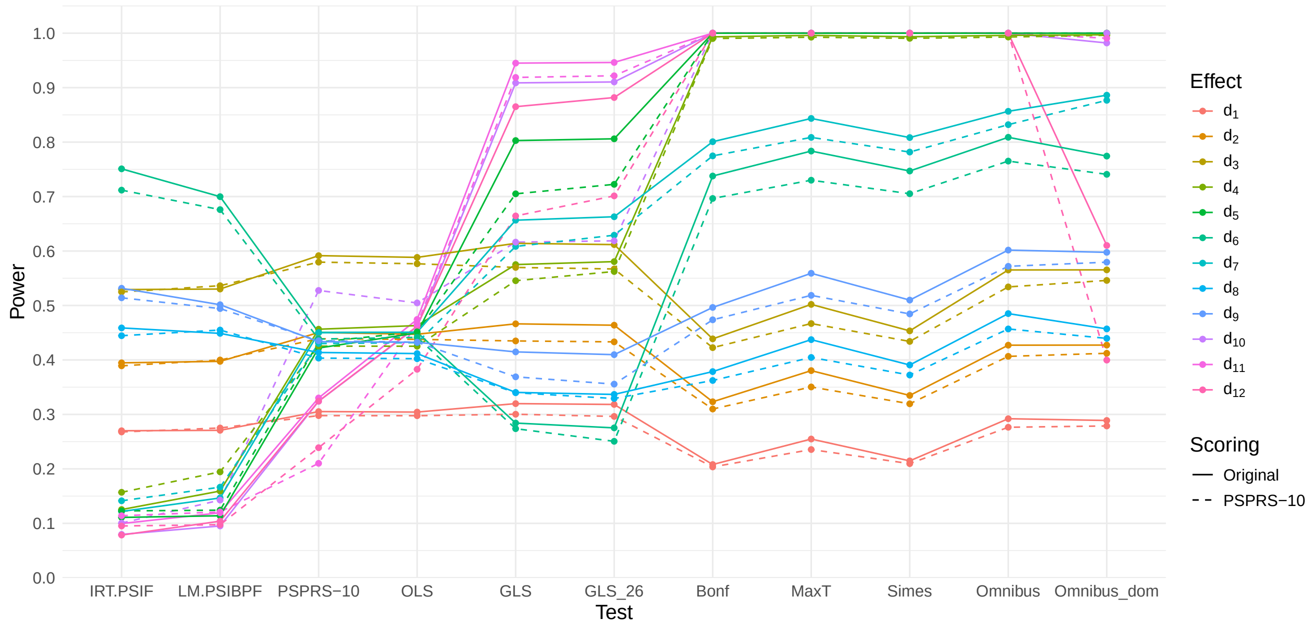

Supplementary Figure 24: Power of the considered testing procedures for the simulation based on discretised multivariate normal scores with the correlations $r_{wd} = 0.8$, $r_{bd} = 0.5$ and $r_{bf} = 0$.

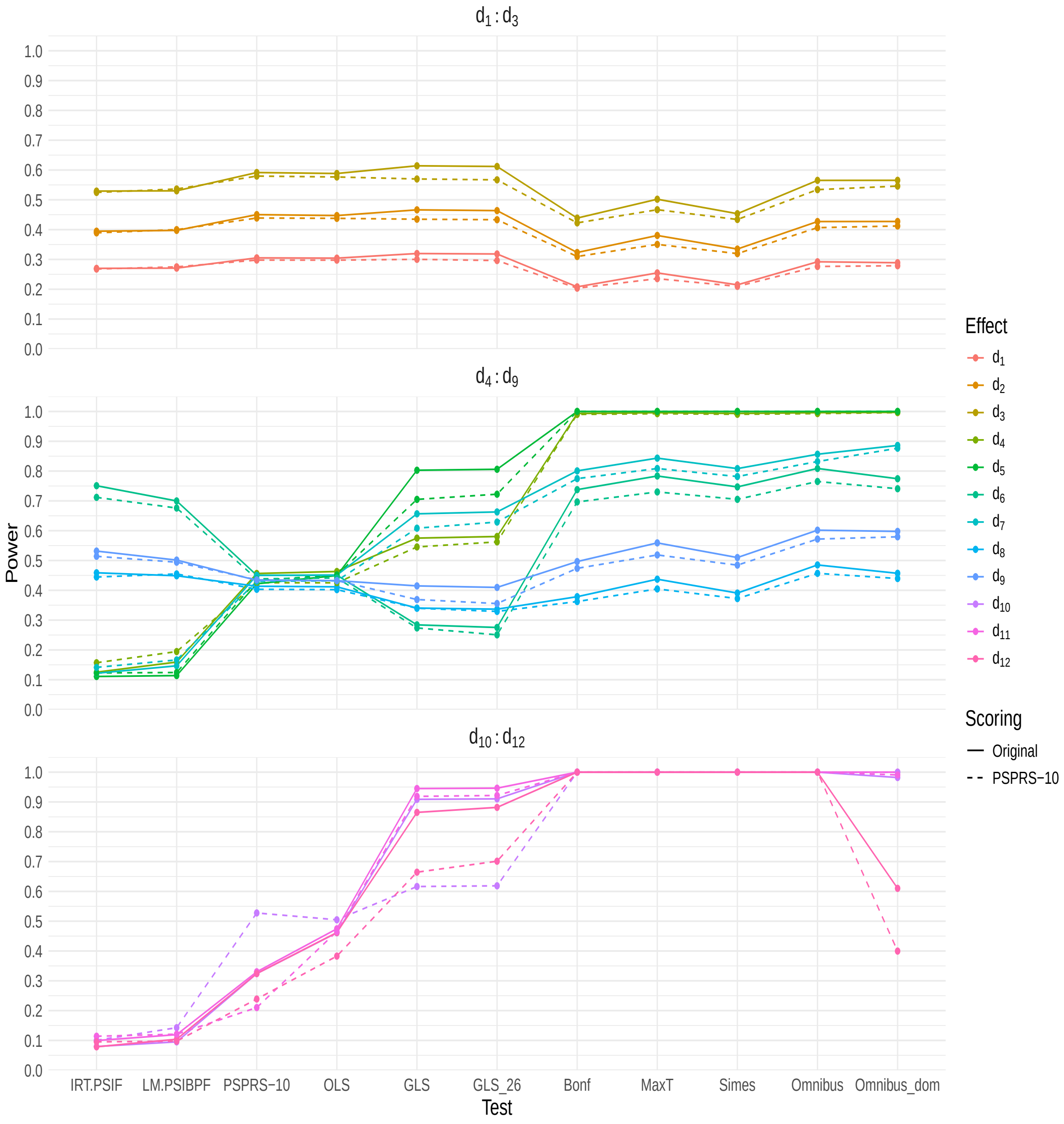

Supplementary Figure 25: Power of the considered testing procedures for the simulation based on discretised multivariate normal scores with the correlations $r_{wd} = 0.8$, $r_{bd} = 0.5$ and $r_{bf} = 0$.

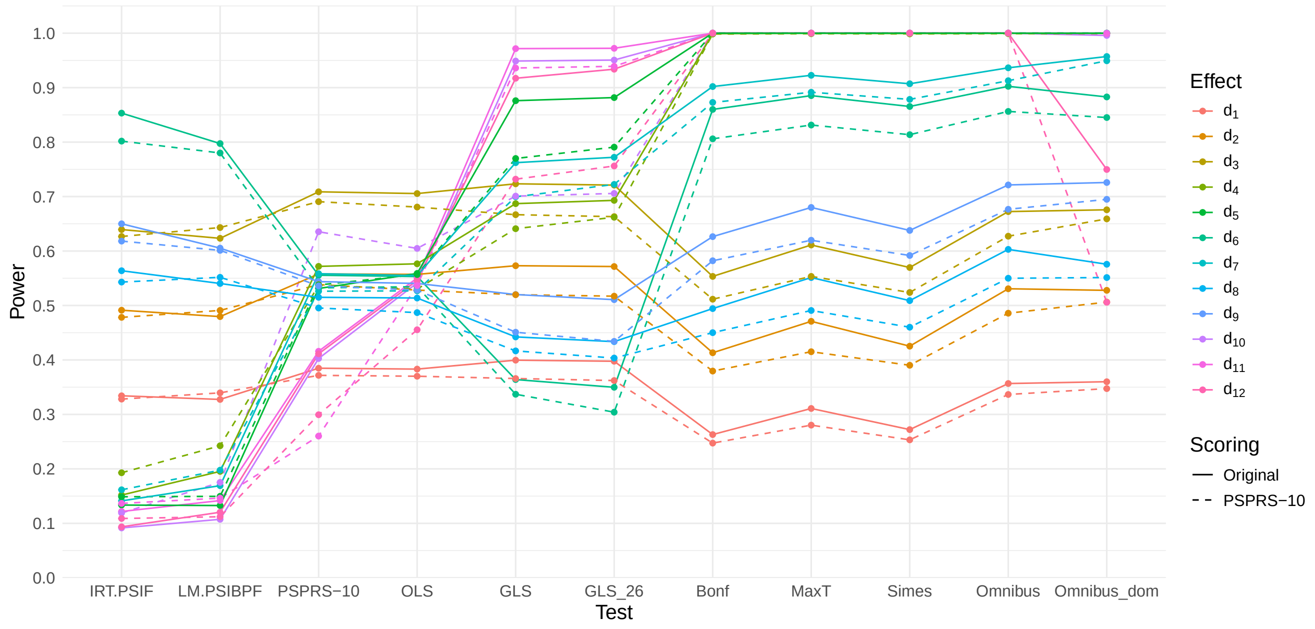

Supplementary Figure 26: Power of the considered testing procedures for the simulation based on discretised multivariate normal scores with the correlations $r_{wd} = 0.8$, $r_{bd} = 0.5$ and $r_{bf} = 0.5$.

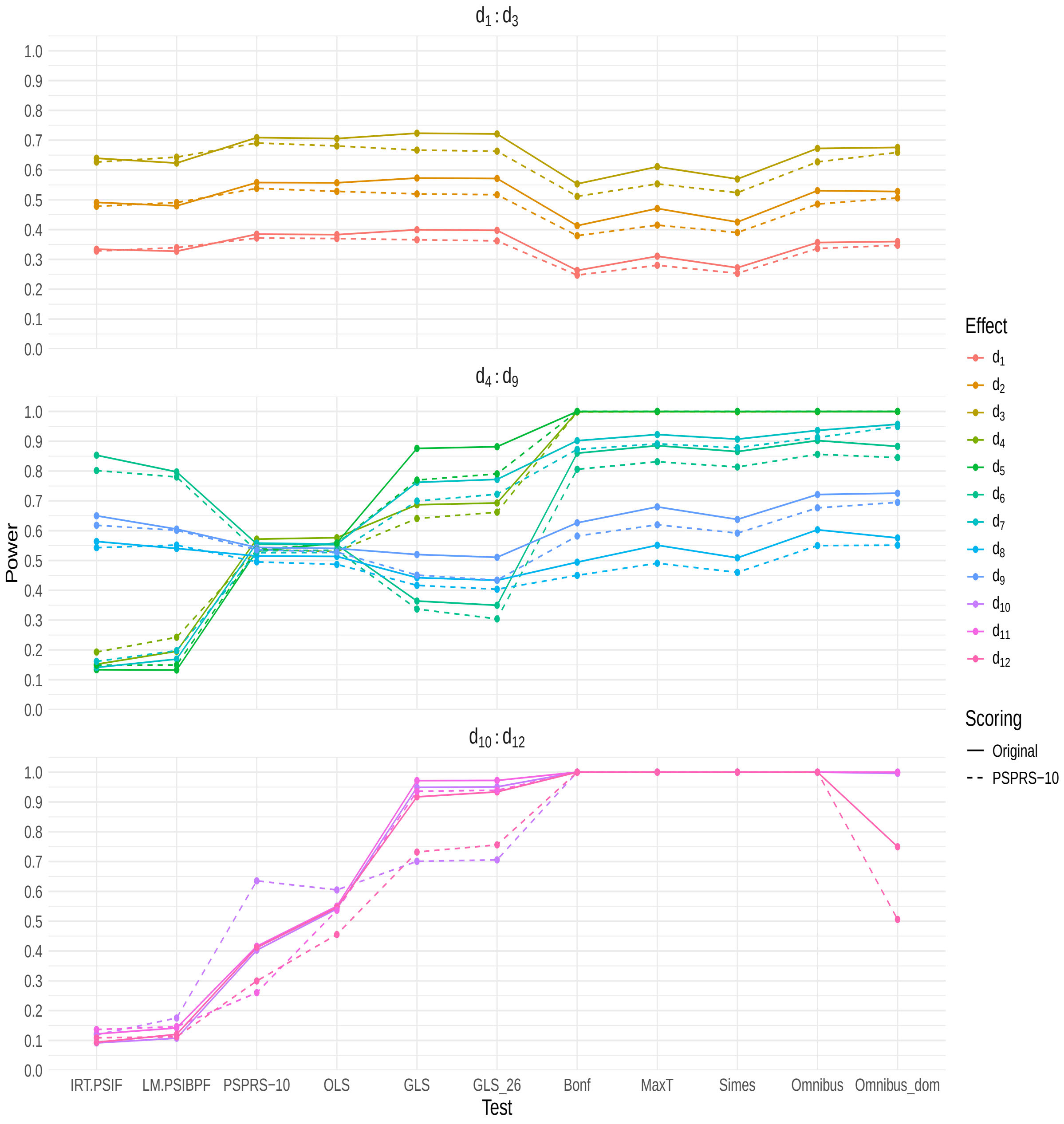

Supplementary Figure 27: Power of the considered testing procedures for the simulation based on discretised multivariate normal scores with the correlations $r_{wd} = 0.8$, $r_{bd} = 0.5$ and $r_{bf} = 0.5$.
